# Impulsivity is a heritable trait in rodents and associated with a novel quantitative trait locus on chromosome 1

**DOI:** 10.1038/s41598-020-63646-9

**Published:** 2020-04-21

**Authors:** Bianca Jupp, Silvia Pitzoi, Enrico Petretto, Adam C. Mar, Yolanda Pena Oliver, Emily R. Jordan, Stephanie Taylor, Santosh S. Atanur, Prashant K. Srivastava, Kathrin Saar, Norbert Hubner, Wolfgang H. Sommer, Oliver Staehlin, Rainer Spanagel, Emma S. Robinson, Gunter Schumann, Margarita Moreno, Barry J. Everitt, Trevor W. Robbins, Timothy J. Aitman, Jeffrey W. Dalley

**Affiliations:** 1 Department of Psychology and Behavioural and Clinical Neuroscience Institute, University of Cambridge, Cambridge, UK; 2 MRC Clinical Sciences Centre, Faculty of Medicine, Imperial College, London, UK; 3 Duke-NUS Medical School, Singapore, Singapore; 4 NYU School of Medicine, New York, USA; 5 School of Psychology, University of Sussex, Brighton, UK; 6 Max Delbruck Centre for Molecular Medicine, Berlin, Germany; 7 Institute of Psychopharmacology, Central Institute of Mental Health, Faculty of Medicine Mannheim, University of Heidelberg, Mannheim, Germany; 8 School of Physiology, Pharmacology and Neuroscience, University of Bristol, Bristol, UK; 9 Centre for Population Neuroscience and Stratified Medicine, Institute of Psychiatry, Psychology and Neuroscience, King¹s College, London, UK; 10 Department of Psychology & Health Research Centre (CEINSA), University of Almería, Almería, Spain; 11 Centre for Genomics and Experimental Medicine, MRC Institute of Genetics & Molecular Medicine, The University of Edinburgh, Edinburgh, UK; 12 Department of Psychiatry, University of Cambridge, Cambridge Biomedical Campus, Cambridge, UK

**Keywords:** Cognitive control, Genetics of the nervous system

## Abstract

Impulsivity describes the tendency to act prematurely without appropriate foresight and is symptomatic of a number of neuropsychiatric disorders. Although a number of genes for impulsivity have been identified, no study to date has carried out an unbiased, genome-wide approach to identify genetic markers associated with impulsivity in experimental animals. Herein we report a linkage study of a six-generational pedigree of adult rats phenotyped for one dimension of impulsivity, namely premature responding on the five-choice serial reaction time task, combined with genome wide sequencing and transcriptome analysis to identify candidate genes associated with the expression of the impulsivity trait. Premature responding was found to be heritable (h^2^ = 13–16%), with significant linkage (LOD 5.2) identified on chromosome 1. Fine mapping of this locus identified a number of polymorphic candidate genes, however only one, beta haemoglobin, was differentially expressed in both the founder strain and F6 generation. These findings provide novel insights into the genetic substrates and putative neurobiological mechanisms of impulsivity with broader translational relevance for impulsivity-related disorders in humans.

## Introduction

Impulsivity describes an individual’s predisposition towards premature, excessively risky, poorly planned and inappropriate actions and decisions^1^. Although acting impulsively can occasionally be advantageous, it more often results in negative outcomes, especially when co-expressed in disorders of personality^2^ and mood^3^, drug addiction^4^, and attention-deficit hyperactivity disorder (ADHD)^5^. As such, impulsivity has emerged as a key dimensional construct in psychiatry with utility as an endophenotype to inform gene discovery and etiological mechanisms for a variety of disorders^6,7^.

Impulsivity is a complex, multidimensional trait mediated by distinct psychological and neural mechanisms^8^. Several groupings have been proposed to capture the evident heterogeneity of impulsivity; for example, impulsive action that depends on self-restraint to avoid or stop a response, and impulsive choice involving preferences for small, immediate rewards over larger but delayed rewards^9–11^. Research into the neural mechanisms of impulsivity has been greatly accelerated by the development of objective, translatable measures of impulsivity in rodents, humans and other species^7^. Indeed, convergent studies in humans and experimental animals suggest that interactions of the ventral striatum, including the nucleus accumbens core and shell (NAcbC, NAcbS), with the prefrontal cortex (PFC) and hippocampus mediate different forms of impulsivity, with important contributions from the ascending dopaminergic, serotonergic and noradrenergic systems^8^.

The personality trait of impulsivity is prevalent in substance use disorders acting as both a determinant and consequence of drug abuse and addiction^12–15^. Findings in humans indicate that impulsivity may be a heritable risk factor for the development of harmful drug use^4^, consistent with a substantial genetic etiology of this disorder^16,17^ and the impulsivity trait^18^. To investigate further the genetic mechanisms of impulsivity we assessed the heritability of a single quantitative measure of impulsivity in male and female rats, premature responding on the 5-choice serial reaction time task (5CSRTT). Premature responding on this task reflects the failure of a subject to inhibit anticipatory responding to a reward-predictive cue – a form of waiting impulsivity^8^ – and has been shown to vary considerably in outbred strains of rats^19–21^. Persisently high levels of premature responding on the 5CSRTT predicts increased escalation of cocaine and nicotine self-administration^19,22^, an increased risk for relapse after punishment- induced drug abstinence^23^ and the subsequent emergence of compulsive cocaine self-administration that persists despite concurrent punishment^24^.

In the present study we established a six-generational pedigree of animals selectively bred for high and low levels of impulsivity on the 5CSRTT. We specifically investigated the heritability of this ‘trait-like’ characteristic prior to carrying out a non-parametric linkage analysis with next generation sequencing to identify and fine map significant quantitative trait loci. Microarray- based gene expression profiling of brain regions previously implicated in impulsive behavior on the 5CSRTT (i.e. NAcbC, NAcbS, PFC) was subsequently carried out to reveal differentially-expressed genes with underlying sequence variation within the linkage region.

## Results

### Impulsivity on the 5-choice serial reaction time task is a heritable trait

**Figure 1:**
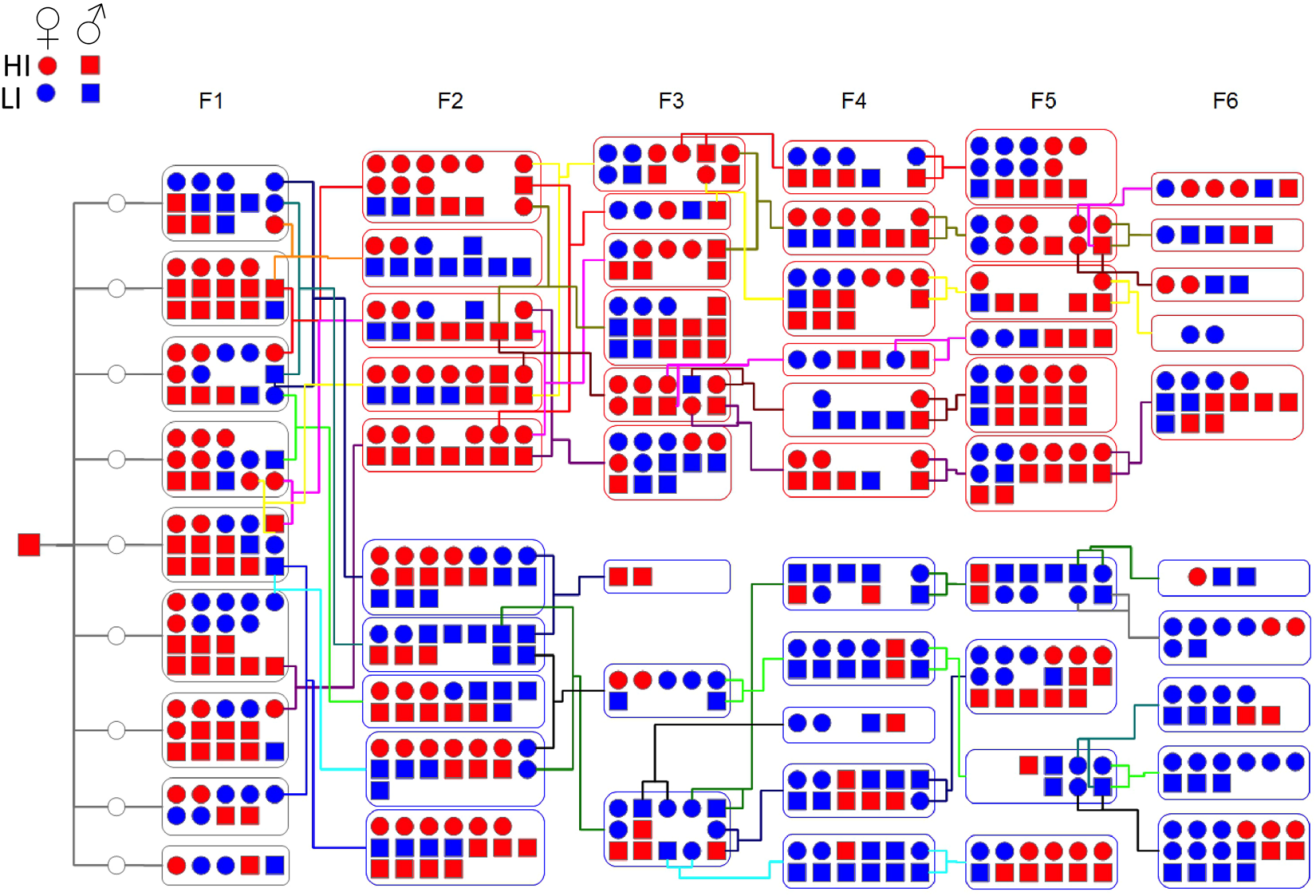
Summary of the multigenerational pedigree structure and breeding scheme used to enrich impulsivity in rat offspring. One HI male (far left) was crossed with 9 non-phenotyped females (represented by open circles). Following phenotyping for impulsivity on the 5CSRTT, high-impulsive male and female rats were crossed. A low-impulsive line was established by crossing low-impulsive male and female rats. Red denotes high-impulsivity (mean number of premature responses ≥ 49); blue denotes low-impulsivity (mean number of premature responses ≤ 48); square boxes denote males; circles denote females.

**Figure 2:**
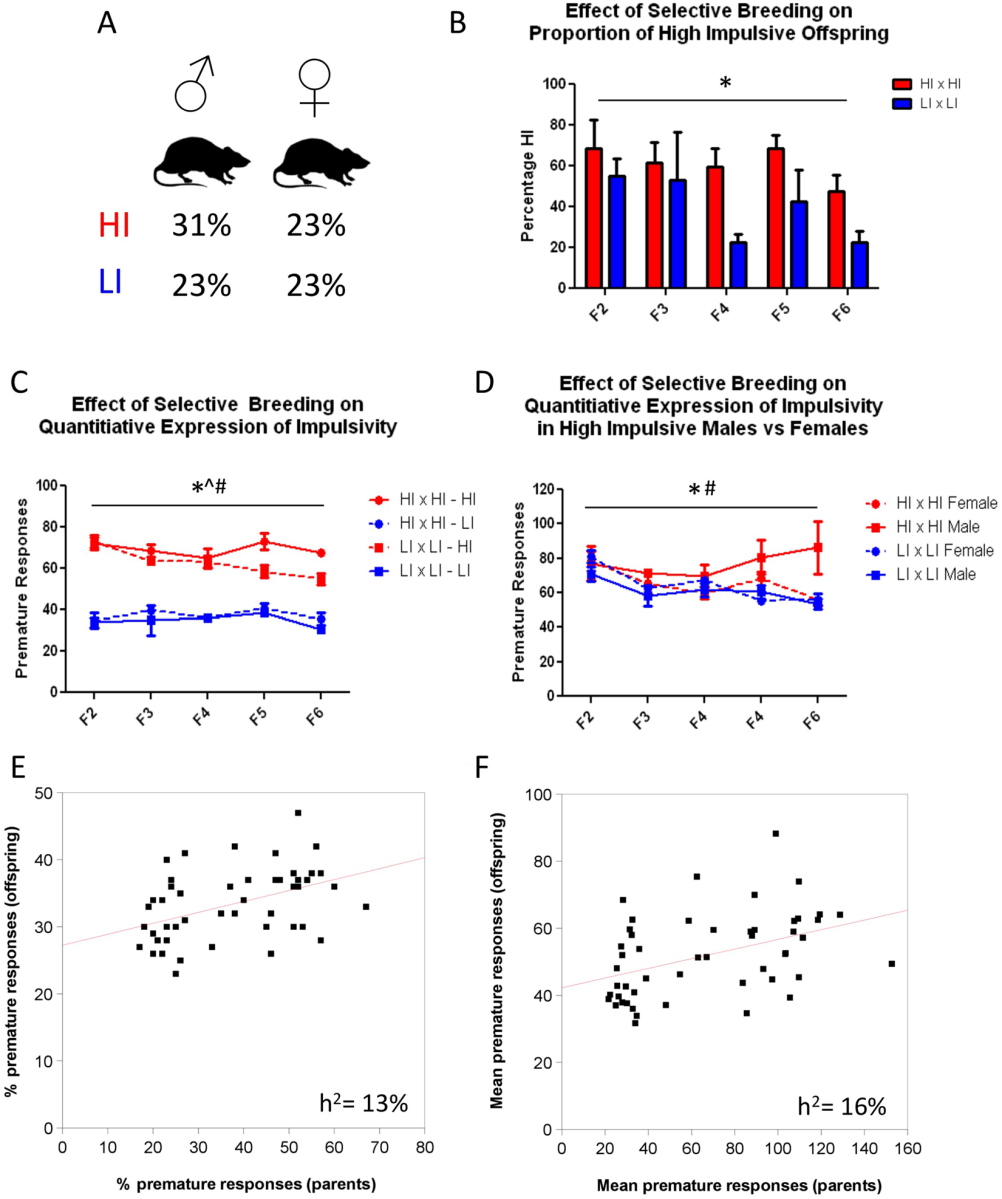
Heritability of impulsivity. (**A**) Percentage of rats expressing either high (HI) and low (LI) impulsivity across the entire pedigree. 54% of rats were HI, while 46% were LI. (**B**) The percentage of offspring in each litter expressing HI was significantly greater in those selectively bred for high (HI x HI) rather than low (LI x LI) impulsivity (*p < 0.01, main effect of breeding, two-way ANOVA). (**C**) The quantitative magnitude of the impulsivity phenotype (average premature responses) was found to be significantly greater in HI offspring of litters bred selectively for HI (HI x HI) compared to LI (LI x LI), while no difference was observed for LI offspring of either lineage (*p < 0.01, main effect of breeding, ^p < 0.05, main effect of phenotype, ^#^p < 0.05, interaction between breeding and phenotype). (**D**) This effect was moderated by gender, with the average premature responding of male HI rats from HIxHI pairings significantly greater than female rats within these litters, as well as male and female rats from LI x LI pairings (*p < 0.05 main effect of breeding, #p < 0.05 breeding x sex interaction). (**E,F**) Mid parental-offspring correlation heritability estimates (h^2^) confirmed a significant heritability of this trait. Shown are **(E)** percentage premature response means in parents (x-axis) and offspring (y-axis) and (**F**) absolute number of premature responses in parents (x-axis) and offspring (y-axis). Results indicate a 44% significant correlation (p = 0.00129 and h^2^ = 16% for data shown in (**E**) and a 43% significant correlation (p = 0.00182) and h^2^ = 13% for data shown in (**F**).

Figure 1 presents an overview of the breeding schema and behavioral phenotypes expressed in each generation of rat offspring. High impulsivity was present in 54% of the entire multi-generational pedigree (Fig. 2A). The proportion of HI rats produced in each litter was significantly greater in litters selectively bred for HI than LI (Fig. 2B; main effect of breeding F(1,39) = 11.26,p < 0.01) and while there was a trend for a change in this proportion over successive generations, this was not significant (F(4,39) = 2.466, p = 0.07). Further, the average quantitative magnitude of the impulsivity phenotype (average premature responses) was found to be significantly greater in HI offspring of parents bred selectively bred for HI compared to LI, while no difference was observed for LI offspring of either lineage (main effect of breeding F_(1,69)_ = 11.087, p<0.01, main effect of phenotype F_(1,69)_ = 401.885, p < 0.00001, breeding x phenotype interaction F_(4,69)_ = 2.879, p < 0.05 MANOVA, (Fig. 2C)). When investigating whether there was a divergence in the quantitative expression of the trait between HI male and females, a significant interaction was observed between gender and breeding (main effect of breeding F(1,51) = 6.090, p < 0.05; interaction F(4,51) = 4.637, p < 0.05, MANOVA), with male offspring of parents bred selectively for HI maintaining higher levels of premature responding than all other groups (Fig. 2D). Analysis by mid-parental-offspring correlation across the entire pedigree confirmed the heritability of this trait and yielded consistent estimates of 0.13 (p < 0.001) and 0.16 (p < 0.001) heritability of both the percentage (Fig. 2E) and absolute (Fig. 2F) number of premature responses, respectively. These findings were confirmed using the intercept model, with an estimated heritability of 0.13 (95% CI, 0.06, 0. 21) for percentage premature responding, and 0.10 (95% CI, 0.04, 0.17) for mean number of premature responses. No difference was observed for heritability estimates when including sex and breeding as random effects (Table 1).

**Table 1:** Heritability estimates for premature responding incorporating different random effects (sex, breeding, sex and breeding).

Random Effects	Heritability Estimate	DIC
Average PR	0.10	5711.77
Average PR + Sex	0.08	5762.312
Average PR + Breeding	0.09	5762.394
Average PR + Sex + Breeding	0.09	5762.285
Percentage PR	0.13	4589.328
Percentage PR + Sex	0.12	4589.924
Percentage PR + Breeding	0.13	4588.048
Percentage PR + Sex + Breeding	0.12	4588.574

No significant difference in heritability was observed when including these variance components (as calculated using deviance information criterion - DIC).

### Premature responding is associated with a region of significant linkage on chromosome 1

**Figure 3:**
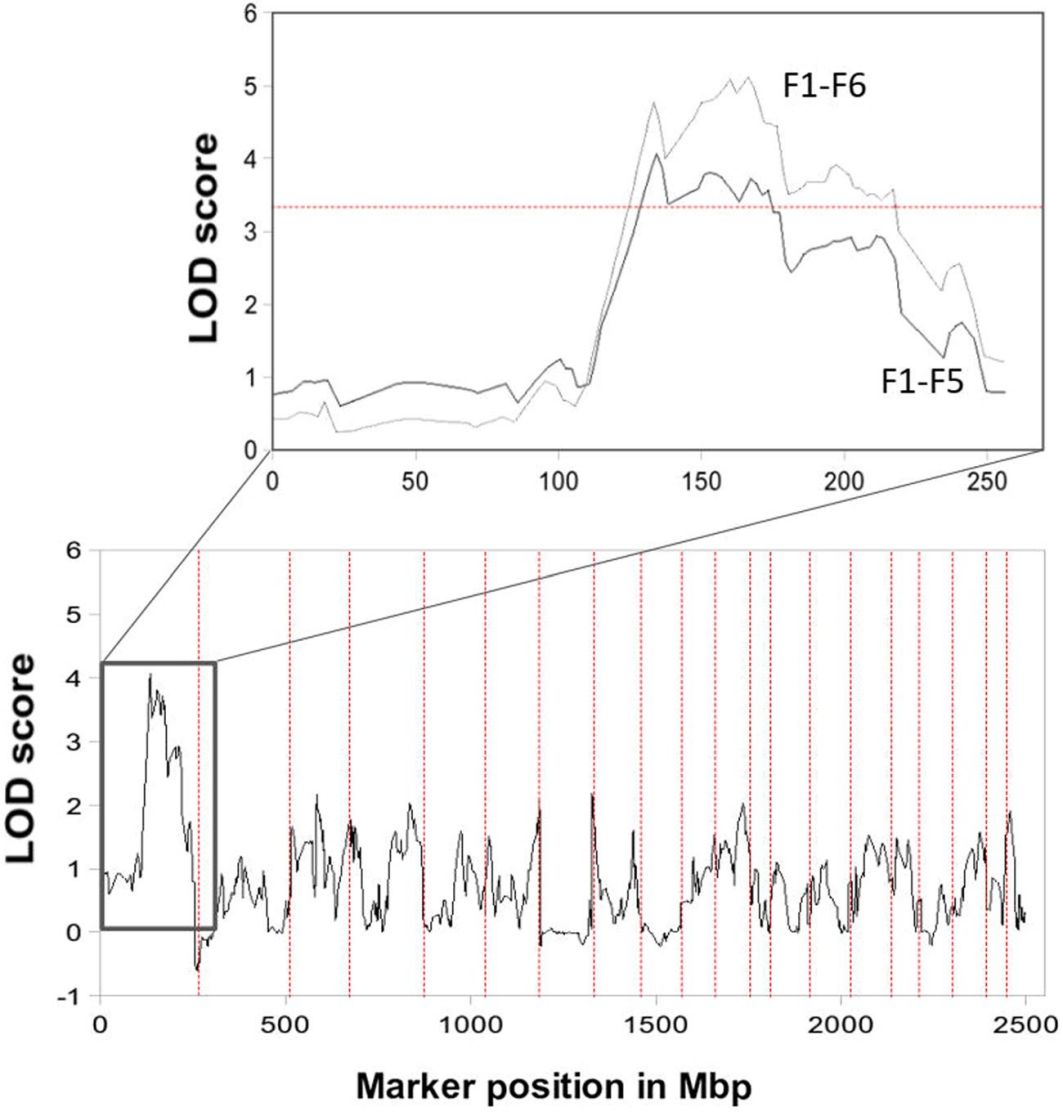
Genome-wide linkage for percentage premature responding. Genome-wide linkage results (F1-F5 generations) on 20 autosomal chromosomes. Dashed red lines indicate chromosomal limits. The impulsivity trait was defined by the percentage number of premature responses on the 5CSRTT (non-parametric linkage). (Inset) Significant linkage results were observed on chromosome 1 (F1- F5 black line; F1-F6 grey line). The horizontal red line depicts the genome-wide significant LOD score threshold of 3.3. Note LOD scores on chromosome 1 increased after the addition of data from the F6 generation.

Our study identified 1,536 single nucleotide polymorphisms (SNPs) in generations F1–5 of the established pedigree. Figure 3 presents an overview of the results of the subsequent genome-wide linkage analysis for percentage premature responding. Using non-parametric linkage analysis to avoid assumptions about the trait distribution and provide weighting to more extreme individuals, we identified a significant QTL for the percentage of premature responses on chromosome 1q31–1q34 with a maximum LOD score of 4.07 (p < 0.05, Fig. 3 inset). We subsequently genotyped the rats of generation six using an additional four heterozygous markers demonstrating the highest linkage signals in the QTL region while showing no linkage disequilibrium. Adding this to the linkage analysis increased the strength of the linkage on chromosome 1, giving a maximum LOD score of 5.2 (p < 0.05, Fig. 3 inset). We identified 307 genes that were located in the approximately 20 cM-spanning region (Supplementary Table S1), some of which have previously identified roles in impulsivity and disorders of impulse control (Supplementary Table S2).

We repeated the genome-wide linkage analysis of the first five generations using the average number of premature responses as the quantitative trait. This identified a second locus at chromosome 1q31–1q34, which resided at ~ 213 Mbp (see Supplementary Fig. S1 for linkage results). Importantly, for each quantitative trait (percentage and absolute premature responding), linkage results were confirmed using different sets of families (obtained by PedCut and Jenti), suggesting the identified QTL was robust and not dependent on the family structure used in the linkage analysis. In order to detect sequence variants potentially associated with impulsivity, in addition to the already analysed SNPs, we carried out whole genome sequencing (WGS) in four extremely-discordant sib-pairs chosen from the 5^th^ generation. The single nucleotide variants and indel sequence variants detected are shown in Supplementary Tables S3 and S4. A total of 58 SNPs and 132 indels were identified within the linkage region.

### Differential gene expression within the identified linkage region in highly-impulsive rats

**Figure 4:**
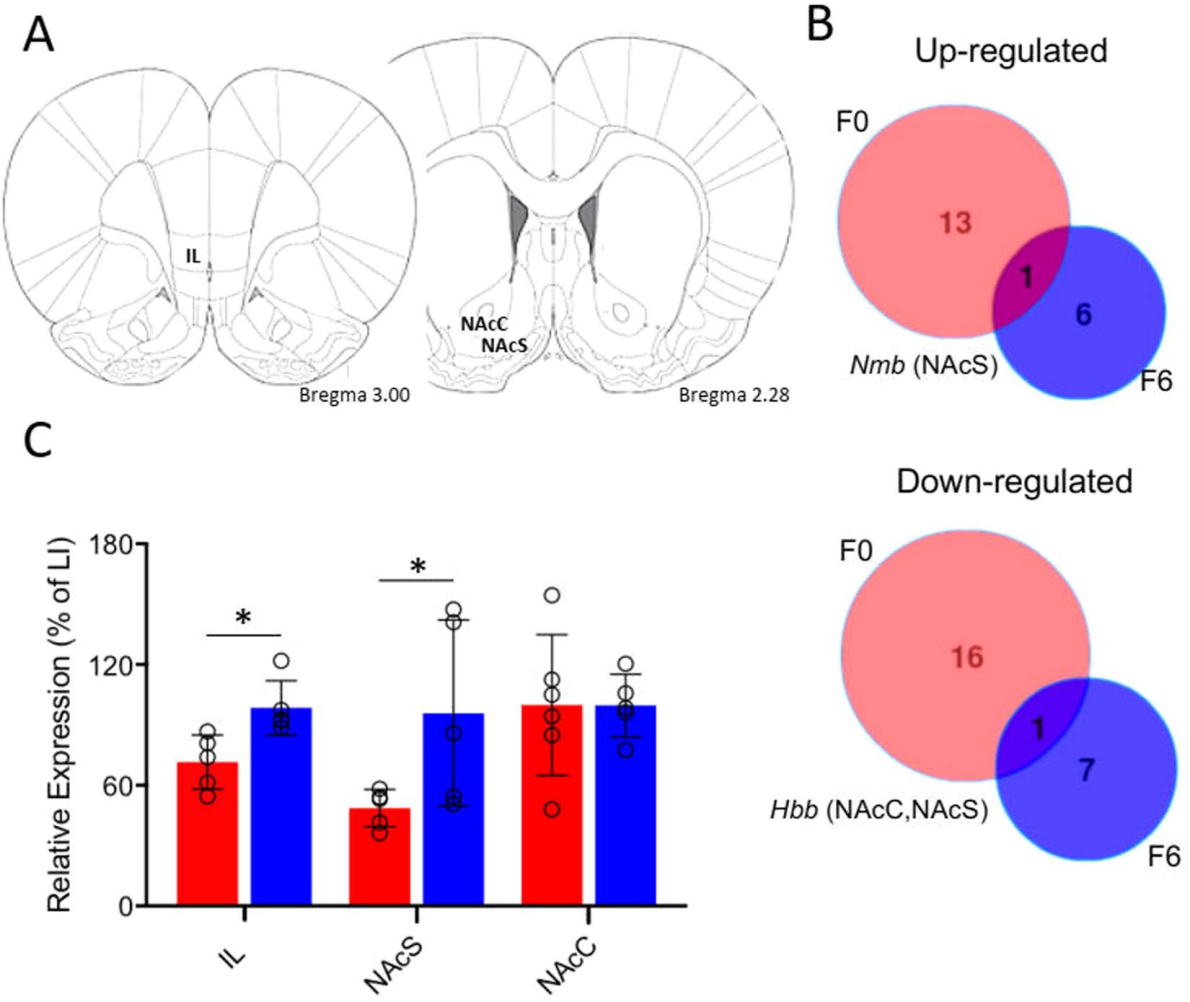
Transcript analysis of regions previously implicated in impulsivity demonstrate significant differential expression between HI and LI rats in both outbred and F6 generations. (**A**) Regions-of-interest for transcript analysis – infralimbic cortex (IL), nucleus accumbens core and shell (NAcC, NAcS) (adapted from^86^). (**B**) Intersection analysis of differentially-expressed genes between the outbred (F0, red) and F6 generations (blue). Commonly-differentiated genes and the brain region in which this occurs are listed. (**C**) Quantitative real time PCR confirmed a significant reduction in the transcript level of *Hbb* in the NAcS and IL cortex of HI rats (red bars) compared with LI rats (blue bars). *p < 0.05.

To identify potential candidate genes within the linkage region we conducted a microarray analysis to investigate differences in transcript levels between HI and LI from both the outbred base population and animals from the sixth generation of the pedigree to identify common alterations in transcriptome expression associated with impulsivity. We investigated transcript expression in the NAcC, NAcS and in the case of sixth generation rats, the ventromedial PFC, or infralimbic cortex (IL) (Fig. 4A). Of the 148 genes within the linkage region represented on the array (Supplementary Table S5), several of these genes were found to be differentially-regulated in HI and LI rats (false discovery rate 10%) based on a non-parametric ranking analysis of the microarray data. A total of 13 transcripts were found to be up-regulated and 16 down-regulated in HI rats of the outbred base population, while 6 transcripts were up-regulated and 7 down-regulated in the F6 generation of HI rats (Table 2). Of these differentially-expressed transcripts, two were found to be consistently differentially-regulated in the same direction, in the same brain region between the two generations examined, *Hbb* and *Nmb* (Fig. 4B).

**Table 2:** Differentially regulated transcripts in high impulsive rats from the outbred base and F6 inbred populations.

Gene symbol	Gene description	False Discovery Rate (associated fold change: high / low impulsivity)		
		NacC	NacS	ILC
**Up-regulated transcripts in high impulsive rats from the outbred base population**				
*Alg8*	Asparagine-linked glycosylation 8, alpha-1,3-glucosyltransferase homolog (S, cerevisiae)	85.4% (1.05)	**6.5% (1.20)**	—
*Atg16l2*	ATG16 autophagy related 16-like 2 (S. cerevisiae)	77.3% (1.03)	**1.0% (1.23)**	—
*Dkk3*	Dickkopf homolog 3 (Xenopus laevis)	25.0% (1.08)	**<0.1% (1.41)**	—
*Fam160a2*	Family with sequence similarity 160, member A2	79.6% (1.05)	**0.1% (1.31)**	—
*Inppl1*	Inositol polyphosphate phosphatase-like 1	92.4% (0.99)	**8.2% (1.18)**	—
*Mesdc1*	Mesoderm development candidate 1	18.7% (1.11)	**8.8% (1.17)**	—
*Mesp1*	Mesoderm posterior protein 1	33.2% (1.08)	**0.6% (1.24)**	—
*Nmb*	Neuromedin B	79.4% (0.69)	0.5% (1.10)*	—
*Nucb2*	Nucleobindin 2	36.4% (1.07)	**0.7% (1.23)**	—
*RGD1561459*	Similar to RIKEN cDNA 1810020D17	100.0% (1.00)	**9.6% (1.19)**	—
*Rlbp1*	Retinaldehyde binding protein 1	100.0% (1.03)	**1.2% (1.24)**	—
*Sv2b*	Synaptic vesicle glycoprotein 2B	**4.3% (1.12)**	82.6% (0.80)	—
*Wee1*	Wee 1 homolog (S. pombe)	100.0% (1.00)	**9.4% (1.18)**	—
**Down-regulated transcripts in high impulsive rats from the outbred base population**				
*Clpb*	ClpB caseinolytic peptidase B homolog (E, coli)	98.6% (0.97)	**0.4% (0.71)**	—
*Far1*	Fatty acyl CoA reductase 1	100.0% (0.97)	**<0.1% (0.58)**	—
*Folh1*	Folate hydrolase 1	—	**0.1% (0.71)**	—
*Grm5*	Metabotropic glutamate receptor 5	—	**0.1% (0.67)**	—
*Hbb*	Hemoglobin, beta	**8.9% (0.89)**	2.3% (0.78)*	—
*Mesdc2*	Mesoderm development candidate 2	84.6% (0.94)	**5.4% (0.80)**	—
*Nmb*	Neuromedin B	**<0.1% (0.69)**	26.3% (1.10)	—
*Olfml1*	Olfactomedin-like 1	100.0% (0.97)	**7.4% (0.82)**	—
*Pak1*	p21 protein (Cdc42/Rac)-activated kinase 1	100.0% (0.99)	**0.4% (0.72)**	—
*Pde3b*	Phosphodiesterase 3B, cGMP-inhibited	**2.6% (0.92)**	27.1% (0.96)	—
*Picalm*	Phosphatidylinositol binding clathrin assembly protein	**8.9% (0.90)**	**<0.1% (0.62)**	—
*Rnf141*	RING finger protein 141	—	**<0.1% (0.54)**	—
*Serpinh1*	Serine (or cysteine) peptidase inhibitor, clade H, member 1	11.6% (0.92)	**0.5% (0.74)**	—
*Spcs2*	Signal peptidase complex subunit 2 homolog (S, cerevisiae)	67.1% (0.94)	**1.6% (0.75)**	—
*Sv2b*	Synaptic vesicle glycoprotein 2b	100.0% (1.12)	**1.2% (0.80)**	—
*Usp47*	Ubiquitin specific peptidase 47	100.0% (0.98)	**3.5% (0.79)**	—
**Up-regulated transcripts in high impulsive rats from the inbred F6 population**				
*Nmb*	Neuromedin-B	**<0.1% (1.46)**	<0.1% (1.81)*	11.9% (1.39)
*Nrip3*	Nuclear receptor interacting protein 3	**8.0% (1.21)**	63.7% (1.09)	35.9% (1.10)
*Pde3b*	Phosphodiesterase 3B, cGMP-inhibited	**3.8% (1.25)**	33.5% (1.14)	15.9% (1.19)
*Sv2b*	Synaptic vesicle glycoprotein 2B	36.9% (1.01)	**1.9% (1.24)**	**5.1% (1.21)**
*Thrsp*	Thyroid hormone responsive	100.0% (0.84)	**8.2% (1.21)**	88.2% (1.03)
*Tm6sf1*	Transmembrane 6 superfamily member 1	**2.2% (1.24)**	100.0% (1.00)	100.0% (1.01)
**Down-regulated transcripts in high impulsive rats from the inbred F6 population**				
*Cpeb1*	cytoplasmic polyadenylation element binding protein 1	38.1% (0.85)	—	**6.0% (0.87)**
*Dkk3*	dickkopf homolog 3	**4.79% (0.85)**	100.0% (1.11)	99.6% (1.11)
*Hbb*	hemoglobin, beta	**1.0% (0.84)**	6.3% (0.85)*	**1.3% (0.95)**
*LOC689064*	beta-globin	**0.9% (0.86)**	11.8% (0.85)	**0.5% (0.95)**
*Ppfibp2*	Protein tyrosine phosphatase, receptor type, F interacting protein, binding protein 2	100.0% (1.04)	100.0% (0.97)	**5.8% (0.77)**
*Prc1*	protein regulator of cytokinesis 1	**5.9% (0.83)**	—	—
*Thrsp*	thyroid hormone responsive	**7.5% (0.84)**	100.0% (1.20)	100.0% (1.03)

False discovery rates and associated fold change for differentially-expressed genes in the identified linkage region in the nucleus accumbens core (NAcC), nucleus accumbens shell (NAcS) and infralimbic cortex (ILC). Bold denotes significantly-altered expression (10% FDR), - denotes not assessed, *differentially expressed in both base and F6 population.

After integration of the differential expression findings with the WGS results, we found that some of the differentially-expressed genes were also polymorphic (*Pde3b, Sv2b, and Hbb*; Supplementary Table S3) or had indels (*Sv2b, Rlbp1, Far1, Grm5, Pde3b, Usp47, Ppfibp2, Prc1*; Supplementary Table S4). Of these genes, only *Hbb* was found to be consistently differentially-expressed in both the base and F6 generations so we sought to confirm its differential expression independently. Quantitative real time PCR from tissue from a separate cohort of phenotyped rats confirmed *Hbb* expression was significantly reduced in the IL cortex (t = 2.062, p < 0.05) and NAcS (t = 1.977, p < 0.05) but not in the NAcC of HI rats (Fig. 4C).

## Discussion

We investigated the heritability and genetic underpinnings of impulsivity in the 5CSRTT, which assesses among other processes, the capacity of subjects to refrain from responding before the onset of a reward-predictive stimulus^8^. Animals that respond persistently before the target stimuli, deemed highly-impulsive, subsequently develop several hallmark features of stimulant addiction compared with low-impulsive subjects^19,22–24^. Thus the present findings carry added significance in informing the etiology of natural variation in premature responding as an endophenotype associated with individual risk for drug addiction^4,25^ and potentially aspects of shortened delay gradients in ADHD mediated by an intolerance for delayed rewards^26^. Using combined genome-wide linkage and transcriptome analysis we report that premature responding in the 5CSRTT is a heritable trait associated with an area of significant linkage on chromosome 1. A number of genes were differentially expressed in low- and high-impulsive rats with corresponding gene variants identified within the linkage region. Consistent with these findings, a recent linkage study of the addiction-relevant trait of novelty reactivity, additionally associated with impulsive responding on the 5CSRTT^27^, also identified areas of significant linkage on chromosome 1^28^, notably with overlap within the region identified in the current study, further implicating this region of the genome with addiction-relevant behaviors.

The heritability estimates we report (13–16%, mid-parental offspring correlate; 10–13% intercept model) for the impulsivity trait are comparable with estimates for other behavioral and physiological phenotypes in rodents^6,29^ and while lower than those reported for quantitative measures of impulsivity measured across inbred mouse^30^ and rat^31^ strains, as well as humans (i.e. delay discounting^32^, stop signal reaction time^33,34^) nevertheless support the conclusion that the impulsivity phenotype on the 5CSRTT is heritable and enriched by selective inbreeding.

The observed heritability of impulsivity is also consistent with other traits shown to confer susceptibility to addiction-like behaviors in rodents. Thus, anxiety^35,36^, novelty reactivity^31,37^, novelty preference^38^ and incentive salience attribution (sign- vs goal-tracking^39^) demonstrate moderate rates of heritability in rats and mice. While there is evidence to suggest the heritability of these susceptibility traits are primarily driven by an inter-relationship with novelty reactivity (e.g.^38,39^), rats impulsive on the 5CSRTT fail to show enhanced measures of novelty reactivity^19,40^. Further, impulsivity and novelty reactivity have been shown to predict distinct aspects of addiction-like behavior^24^ suggesting the addiction vulnerability phenotype in rats may involve multiple genetic contributions. Given there was no difference in the proportion of HI vs LI rats across each generation it is unlikely the heritability of impulsivity was driven by a relationship with an indirect variable such as maternal behaviour^41^.

The expression of the impulsivity phenotype, as measured by premature responding in the 5CSRTT, was moderated by sex, with HI male offspring, specifically bred for high impulsivity maintaining quantitative extremes of their phenotype across multiple generations, with females and males bred for low impulsivity showing lower levels of impulsivity. This observation aligns with clinical studies demonstrating the influence of gender on impulsivity, with greater heritability of this trait in males^42^, which in turn may underlie sexual dimorphism observed in the expression of related neuropsychiatric disorders such as addiction^43^ and ADHD^44^. However, no significant difference was observed when incorporating the effect of breeding and sex into trait heritability estimates. Further, no evidence of linkage was observed on either sex chromosome, suggesting that alternative mechanisms may underlie the observed sex-dependent breeding effects; for example as a result of sex-specific regulatory elements on autosomal gene expression^45^ and/or the impact of circulating sex hormones on 5CSRTT performance^46^.

Since more than 300 genes were present within the identified linkage region, including 126 polymorphic genes, we utilised a transcriptome analysis to investigate gene expression in key brain regions of the ‘waiting impulsivity’ network^8^. This network of corticostriatal structures encompasses parallel and interacting circuits involving the NAcbC and NAcbS with the infralimbic cortex^47^, cingulate cortex^48^, insula^49^ and ventral hippocampus^50^. Rats expressing the HI phenotype show a reduction in D2/3 receptor availability in the NAcb^19^, decreased grey matter density and γ-amino-butyric acid (GABA) levels and markers of GABA function and dendritic spine density in the NAcb^51,52^, and structural abnormalities in the insula^49^.

A number of transcripts located within the linkage region were differentially regulated between high and low impulsive rats, some of which were also sequence variant. Notably, some of these genes have previously described roles in impulsivity and associated disorders including: *Grm5* (metabotropic glutamate receptor 5) shown recently to modulate impulsivity^53^ and cocaine reinforcement^54,55^ and whose deletion is associated with ADHD^56^; *Sv2b* (Synaptic vesicle glycoprotein 2B) implicated in neurotransmitter release through regulation of the synaptic release vesicles and exocytosis^57,58^; *Rlbp1* (retinaldehyde binding protein 1) associated with alcohol preference in mice^59^.

Of these identified polymorphic candidates, only one gene, *Hbb*, demonstrated significantly altered gene expression in both the founder strain and generation six of the pedigree. This finding was additionally confirmed in an independent cohort of impulsive rats, which showed a significant reduction in the level of this transcript in the NAcS and IL cortex, potentially implicating a novel role for this gene in impulsivity. *Hbb* encodes for the beta chain subunit of the haemoglobin complex and is traditionally considered in the context of oxygen transport in vertebrate blood erythrocytes^60^. However, Hbb is also expressed in neurons of invertebrates, rats and humans^61–64^ where it is hypothesised to contribute to oxygen homeostasis and mitochondrial respiration^61^. It is therefore not clear whether the observed reduction in *Hbb* transcript in HI rats represents blood or brain hemoglobin. It is possible that differences in expression may reflect a regional perfusion deficit. However, transcript levels for other blood proteins present on the array (e.g. thrombin, alpha-2 macroglobulin) were unaltered between HI and LI rats, suggesting the reduction in Hbb was more likely a neuronal or glial-derived transcript.

Hbb expression is highest in midbrain dopamine neurons, striatal GABAergic neurons, cortical pyramidal neurons and glial cells^62–64^ with reductions in neuronal haemoglobin immunoreactivity reported in Alzheimer’s and Parkinson’s disease^65^. Thus, low expression may be one variable contributing to oxidative stress and neuronal abnormalities in corticostriatal networks related to impulsivity. Since GABAergic medium spiny neurons (MSNs) are highly susceptible to oxidative stress after transient global cerebral ischemia^66^ with prenatal ischemia leading to reduced dendritic branching and spine density in MSNs^67^, we hypothesise that impaired neuronal *Hbb* function may increase the vulnerability of MSNs to oxidative stress with consequent effects on dendritic spine density and function in MSNs of the NAcb, potentially as a manifestation of antenatal and developmental hypoxia. This conclusion could be tested by tissue-specific interference of *Hbb* in relevant brain regions.

We report a number of transcriptomic alterations associated with impulsive behavior in the 5CSRTT. However, genetic variation linked to impulsive behavior may also have other functional effects on the genome; for example, through epigenetic modification and variation in RNA splicing proteins. In addition, since our gene expression analysis was limited to adult tissue, we cannot discount the possibility that differential expression of these genes within the QTL affected key stages of neurodevelopment, which in turn affected the function of the mature ‘waiting impulsivity’ network in adults.

The endpoint of this study was to demonstrate the heritability of the impulsivity trait in rats and to identify linkage of discrete chromosomal regions with impulsivity, as indexed by premature responding in the 5CSRTT. Our findings indicate that impulsive responding in the 5CSRTT is a heritable trait and therefore a valid endophenotype to investigate the brain mechanisms of premature responding and more broadly waiting impulsivity. Our integrative approach enabled us to identify a target locus harboring candidate genes relevant to the etiology of impulsivity, with translational potential to inform our understanding of analogous forms of impulsivity present in ADHD, drug addiction, depression, and other impulsivity-related disorders^8^.

## Methods

### Experimental subjects

Founding male and female adult Lister-hooded rats were purchased from Charles River (Margate, Kent), aged 2–3 months. Behavioral training commenced 1 week after acclimatisation to the animal house (for the founding generation) or at 3 months of age for derived animals. Water was available *ad libitum* with sufficient food provided to maintain body weights at no less than 90% of free-feeding weights (20 g chow/day). Rats were housed four per cage, under temperature- and humidity-controlled conditions and a reversed 12-hour light/dark cycle (white lights off/red light on at 07:00 h). All regulated procedures conformed to the Animals (Scientific Procedures) Act of 1986 and approved by the animal welfare ethical review board at the University of Cambridge.

### Behavioral training

The founding cohort of 48 male adult rats was trained daily on the 5CSRTT, as described previously^68^. A PC using WhiskerServer software and FiveChoice client^69^ controlled the behavioral chambers (Med Associates Inc, VT, USA). Rats were trained to nose-poke into one of five apertures, for a food-predictive, light stimulus. Correct responses were rewarded, while incorrect, premature and omitted responses were punished with a 5 s time-out. Daily training sessions consisted of 100 discrete trials or 30 minutes, which ever was shorter. Each trial was initiated by the entry of the animal into the food magazine. Following an inter-trial interval (ITI) of 5 s, a brief light stimulus (0.7 s in duration) was presented randomly in one of the five apertures. A nose poke into the corresponding aperture was rewarded with delivery of one food pellet (45 mg Noyes dustless pellets, Sandown Scientific, UK). Failure of the animal to respond within 5 s (‘omission’) or a nose poke into the incorrect aperture (‘incorrect’ response) resulted in a 5 s time-out, during which time no new trials could be initiated and the house light was extinguished. A nose poke response prior to the onset of the light stimulus (‘premature’ response) also resulted in a 5 s time-out. Daily training sessions continued for 8–12 weeks (5 daily sessions/week) until performance on the task was stable (response accuracy ≥75%, omissions ≤20%).

### Impulsivity phenotyping

Screening for impulsivity involved challenging the animals with a series of long inter-trial interval (LITI) sessions where the waiting interval leading up to the presentation of the light stimulus increased from 5 s to 7 s. A single LITI challenge session was employed once a week, on three occasions, with two baseline sessions (i.e. ITI 5 s) on the days leading up to the LITI session and two baseline sessions after the LITI challenge. LITI sessions consisted of 100 trials and a session duration of 45 min. The LITI challenge ended when rats had completed 100 trials or when 45 min had elapsed. Rats were ranked for their level of impulsivity based on the number of premature responses across the three LITI challenge session. Animals making on average ≥ 49 premature responses across the three challenge sessions were deemed high impulsive (HI), while rats making on average ≤ 48 premature responses were deemed low impulsive (LI). Impulsivity phenotypes (HI, LI) were quantified using measures of both percentage premature (number of premature responses/correct + incorrect + omission trials) and absolute number of premature responses averaged across the three challenge sessions. Screening for impulsivity required approximately 5 months from the start of training on the 5CSRTT.

### Breeding schema

A six-generation experimental cross was generated (n = 629), derived from one male HI proband from the founder cohort and nine non-phenotyped females, followed by a selective breeding strategy from generation 1 to 6, according to impulsivity phenotype. The F2 and F3 generations were derived from pairs of rats of the same impulsivity phenotype but from different litters, to enrich for alelles involved in the expression of this trait, before selective breeding, where the most extreme male and female individuals from each litter were mated to establish HI and LI lines respectively and to reduce genetic variability, limiting epistatic effects and the impact of alelles unrelated to the expression of the impulsivity trait on transcriptome analysis. Individual pairings are shown in Fig. 1. Breeding-females were no older than 7 months so to lessen the impact of aging on fertility, while the age of males varied between 7–9 months. Occasionally the same male was crossed with more than one female. Females were palpated for pregnancy and once confirmed, housed individually. Offspring were weaned at post-natal day 60 and housed and phenotyped for impulsivity, as described above. Breeding and behavioral phenotyping of each generation took on average 10 months. Following the assessment of impulsivity status, animals that did not continue in the breeding program were terminally anaesthetized by CO_2_-induced asphyxiation and their brains and spleens excised and frozen over liquid nitrogen for storage at −80 °C for later analysis.

### Linkage analysis and heritability estimates

Genomic DNA was purified from spleen samples (Maxwell® 16 Instrument, Promega, Hampshire, UK) and diluted to a final concentration ≥40 ng/µl. To attribute the contribution of variability in impulsivity to genetic factors we estimated trait heritability using the correlation between the quantitative phenotype of parents and offspring using R/QTL^70^, in generations F2-F6, and confirmed using linear variance component models using MCMCglmm package^71^ in R to account for the in-breeding within our pedigree, with sex and breeding as random effects. All variance components models were run with a minimum of 1 × 10^6^ iterations and burn-in periods of 1 × 10^4^. Convergence of each model was tested using the Heidelberg stationary test. Posterior mean of heritability estimates are reported.

To evaluate the heterogeneity of sequence variation in the Lister-hooded strain genome and to select polymorphic markers to be genotyped in the linkage study, we carried out pilot genome-wide genotyping (1-million SNPs panel, Affymetrix, High Wycombe, UK) on six rats (three males and three females) randomly chosen from different families of the colony, from generations two, three and four of the experimental cross. We selected ~118,000 markers according to their Polymorphism Information Content (0.37 < PIC < 0.77) and NO CALL ratio (≤0.3) and submitted them to Illumina (San Deigo, USA) for assay development evaluation. We then used the final Illumina genotyping score ≥0.5, position and distance (at least 1.5 Mbp distance between consecutive markers), to define a final data set of 1,536 SNPs that were used to genotype generations 1–5 of the experimental cross using the custom Illumina GoldenGate Bead Array.

Genotype calling was performed using Alchemy^72^, an algorithm designed for SNP calling in highly homozygous populations. It produces posterior probabilities for AA, AB and BB genotypes: P(AA); P(AB); and P(BB), even if the assay-failure probability P(NC) is high. In that case the correct P(call) is the second highest posterior probability. To call reliable genotypes we applied a quality call score threshold of 0.8 and defined P(call) <0.8 as a “no call”.

Linkage analysis was carried out in MERLIN^73^ using variance component (VC) and non-parametric linkage (NPL) so to avoid confounds associated with the inbred nature of our pedigree (e.g.^74^). Further to this, and given MERLIN is not suitable for very large and complex pedigree structures, the original pedigree was divided into smaller families (max-bit-size = 24), using two different algorithms, PedCut^75^ and Jenti^76^, so to replicate our linkage findings. Mendelian inconsistencies were checked with Pedcheck^77^ and zeroed (5%). Hardy Weinberg Equilibrium (HWE) were tested with Pedstats^78^ and markers with HWE *p* < 0.0001 were removed (n = 56). A total of 894 autosomal polymorphic SNPs were selected for the final linkage analysis (Supplementary Table S6).

Following identification of a significant quantitative trait locus (QTL) from family data from generation 1 to 5, we generated an additional cross (F6) and carried out an additional linkage analysis adding data from this generation to the families (n = 76), to confirm segregation of the locus with impulsivity. An additional four heterozygous markers (Supplementary Table S7) were chosen from those giving the highest linkage signals in the QTL region and showing no linkage disequilibrium (LD). LD was calculated in R and markers were genotyped with custom TaqMan SNP genotyping assays.

### Whole genome sequencing

To fine map the QTL region of interest and analyze a denser polymorphic-loci map between HI and LI rats, we sequenced the genome of four extremely-discordant sib-pairs from generation 5, with high throughput sequencing. Illumina paired-end reads were mapped to the reference Brown Norway (BN) genome RGSC-3.4^79^ using the read alignment software Burrows-Wheeler Aligner (BWA-0.5.8c)^80^ with default parameters. Genomic variants, which included single nucleotide (SNV) and short insertion/deletions (indels) (1–15 bp), were detected using the Genome Analysis Toolkit (GATK version 1.0.6001)^81,82^ with default parameters. Before calling variants, data were pre-processed by masking of clonal reads using Picard tools (https://sourceforge.net/projects/picard/), realignment of reads around potential indels, and recalibration of base quality scores using GATK^81^. To exclude potential false positives, variant quality was recalibrated for both SNVs and indels. Polymorphic SNVs and indels where alleles in at least one rat differed from the reference BN rat genome were then extracted from the variant data.

### Gene expression analysis

Brains from HI and LI rats derived from sixth generation animals and an additional cohort of the original outbred base population (HI, n = 6; LI, n = 6 for each cohort) were sectioned into 120 µm coronal slices using a Leica CM 3000 Cryostat (Leica, Wetzlar, Germany) at −20 °C. Based upon previous studies implicating the infralimbic cortex (IL), nucleus accumbens core (NAcbC) and shell (NAcbS) in the expression of impulsivity on the 5CSRTT^83^, these regions were bilaterally microdissected using biopsy needles (Stoelting, Wood Dale, IL, USA) for RNA extraction and subsequent transcriptome analysis.

### RNA isolation and purification for transcriptome analysis

One-ml of TRIzol reagent (Life Technologies, Darmstadt, Germany) was added to each of the punched tissue samples. The resulting suspensions were homogenized by 20 passages through a 22 gauge needle. Chloroform was added and the RNA isolated by phase separation after centrifugation. The RNA-containing upper phases were carefully removed and purified with an RNeasy MinElute Cleanup Kit (Qiagen, Hilden, Germany) and Genomic DNA eliminated with a DNase step (Qiagen). RNA yield and purity were assessed with a NanoDrop 1000 spectrophotometer (Peqlab, Erlangen, Germany) and samples having optical density 260/280 measurements in the range of 1.8 to 2.2 were kept for further analysis. RNA integrity was determined using an Agilent 2100 Bioanalyzer (Agilent Technologies, Santa Clara, CA, USA); only samples with RNA integrity values above 8 were used.

### Affymetrix GeneChips

RNA samples were subjected to microarray gene expression profiling. One-hundred ng of total RNA was amplified and labelled using the GeneChip 3’IVT Express Kit (Affymetrix, High Wycombe, UK) according to the manufacturer’s protocol. Labelled RNA was hybridized on GeneChip Rat Genome 230 2.0 Arrays according to protocol and scanned using an Affymetrix GeneChip Scanner 3000 run with Affymetrix GeneChip Command Console Software (AGCC) at the microarray core facility at the University Hospital Mannheim. The obtained cell intensity (CEL) files were quality assessed using the array analysis Affymetrix quality control pipeline (www.arrayanalysis.org) in R 14.2 software environment. Probe sets were summarized according to updated Entrez Gene definitions, version 14, and data subsequently quantile-normalised using the robust multi-array average function of the Affymetrix package from the Bioconductor project^84^. Genes with expression values below 80 were excluded to avoid confounding array background noise.

### Statistical analysis and data mining of microarrays

The gene expression data were filtered to remove low-expression and poorly annotated genes, which resulted in a set of 8000 genes for subsequent statistical analyses. Differential expression analysis was carried out using the “RankProd” function in R, which is based on the calculation of non-parametric rank products and accounts for multiple testing by calculating the percentage of false predictions and is equivalent to the false discovery rate^85^.

### Quantitative PCR

We identified one gene within the linkage region that was polymorphic and showed consistent changes in gene expression in both the founder and F6 strains. To confirm the findings of the array, we assessed transcript levels for this gene, *Hbb*, using quantitative real time polymerase chain reaction (qRT-PCR) from a separate cohort of outbred Lister Hooded rats (n = 6 HI, n = 6 LI). Total RNA was prepared from tissue biopsies specifically from the NAcS, NAcC and IL cortex as described previously by use of an RNAeasy Total RNA kit (Qiagen, Manchester, UK) and reverse transcribed using the RT2 first strand kit (Qiagen) according to the manufacturer’s protocol. RT-PCR amplification was performed using a QFX96 PCR detection system (Biorad, Watford, UK) with the use of RT2 SYBER Green Master Mix (Qiagen) with RT2 qPCR primer assay for each gene of interest (Qiagen). *ACTB*, *HPRT*, and *TBP* were used as housekeeping genes and samples run in triplicate. Relative gene expression was calculated using the delta Ct method.

## Supplementary information

Supplementary Information.

## Data Availability

The datasets used and/or analysed during the current study are available from the corresponding author on request.
