# Supplementary material for: Impulsivity is a heritable trait in rodents and associated with a novel quantitative trait locus on chromosome 1
Source: Sci Rep. 2020 Apr 21;10:6684. doi: 10.1038/s41598-020-63646-9 (PMC7174407; doi:10.1038/s41598-020-63646-9)
Supplement: Supplementary file 1 — Supplementary Information. [file 41598_2020_63646_MOESM1_ESM.pdf]

Impulsivity is a heritable trait in rodents and associated with a novel quantitative trait locus on chromosome 1

Bianca Jupp[1+], Silvia Pitzoi[2+], Enrico Petretto[2,3+], Adam C. Mar[1,4], Yolanda Pena Oliver[5], Emily R. Jordan[1], Stephanie Taylor[1], Santosh S. Atanur[2], Prashant K. Srivastava[2], Kathrin Saar[6], Norbert Hubner[6], Wolfgang Sommer[7], Oliver Staehlin[7], Rainer Spanagel[7], Emma S. Robinson[8], Gunter Schumann[9], Margarita Moreno[10], Barry J. Everitt[1], Trevor W. Robbins[1], Timothy J. Aitman[11], Jeffrey W. Dalley[1,12,†]

Department of Psychology and Behavioural and Clinical Neuroscience Institute, University of Cambridge, Cambridge, UK[1] MRC Clinical Sciences Centre, Faculty of Medicine, Imperial College, London, UK[2], Duke-NUS Medical School, Singapore[3], NYU School of Medicine, New York, USA[4], School of Psychology, University of Sussex, Brighton, UK[5], Max Delbruck Centre for Molecular Medicine, Berlin, Germany[6]. Institute of Psychopharmacology, Central Institute of Mental Health, Faculty of Medicine Mannheim, University of Heidelberg, Heidelberg, Germany[7], School of Physiology and Pharmacology, University of Bristol, Bristol, UK[8], Centre for Population Neuroscience and Stratified Medicine (PONS), Institute of Psychiatry, Psychology and Neuroscience, King¹s College, London[9], Department of Psychology, University of Almería, Carretera de Sacramento, Almería, Spain[10], Centre for Genomics and Experimental Medicine, MRC Institute of Genetics & Molecular Medicine, The University of Edinburgh, Edinburgh, UK[11], Department of Psychiatry, University of Cambridge, Cambridge Biomedical Campus, Cambridge, UK[12]

+ joint first authors

*Author for correspondence: Professor Jeffrey W. Dalley, Department of Psychology, University of Cambridge, Downing St, Cambridge CB2 3EB, UK. Tel. +44 (0)1223 765 291. Fax. +44 (0)1223 333 564. Email. jwd20@cam.ac.uk

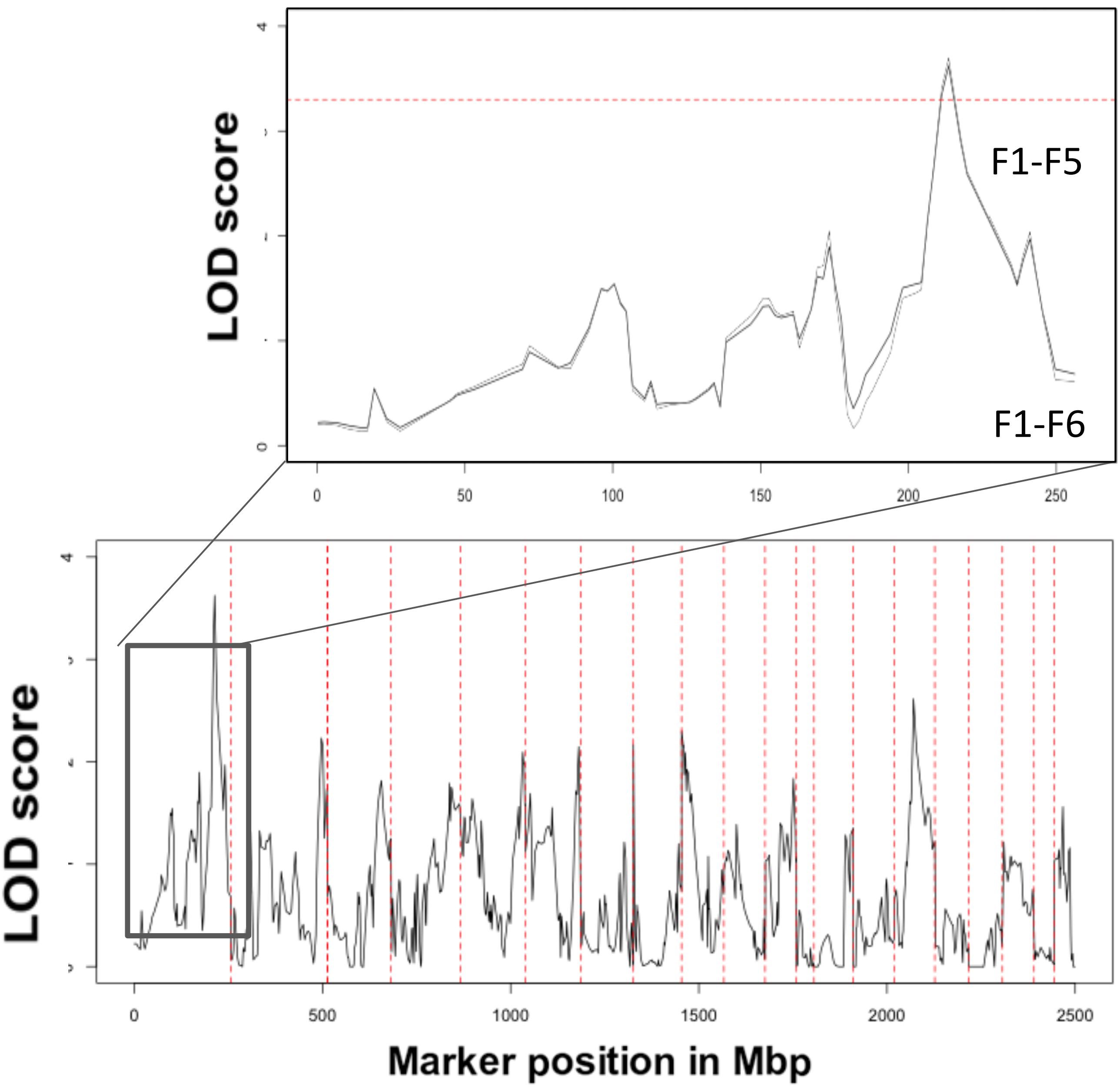

Supplementary Figure 1: Genome-wide linkage for average total premature responses. Genome-wide linkage results (F1-F5 generations) on 20 autosomal chromosomes. Dashed red lines indicate chromosomal limits. The impulsivity trait was defined by the average total number of premature responses on the 5CSRTT (non-parametric linkage). **(Inset)** Significant linkage results are observed on chromosome 1 (F1- F5 black line; F1-F6 grey line). The horizontal red line depicts the genome-wide significant LOD score threshold of 3.3. Note no significant difference in LOD scores on chromosome 1 after the addition of data from the F6 generation.

Gene symbol	Gene description	Gene location (Mbp)
Rgma	repulsive guidance molecule A	128.547399
Chd2	chromodomain-helicase-DNA-binding protein 2	128.609727
St8sia2	Alpha-2,8-sialyltransferase 8B	129.158714
snoU109	Small nucleolar RNA U109	129.245228
Sv2b	Synaptic vesicle glycoprotein 2B	130.683153
Akap13	A kinase (PRKA) anchor protein 13	131.224569
U3	Small nucleolar RNA U3	131.537652
Klhl25	Ectoderm-neural cortex protein 2	131.540949
Agbl1	Uncharacterized protein	131.756899
U1	U1 spliceosomal RNA	133.650066
Ntrk3	NT-3 growth factor receptor	133.92553
Mrpl46	39S ribosomal protein L46, mitochondrial	134.498792
Mrps11	28S ribosomal protein S11, mitochondrial	134.507836
Det1	DET1 homolog	134.549457
rno-mir-7a-2	rno-mir-7a-2	134.607264
Aen	Apoptosis-enhancing nuclease	134.615998
Isg20	interferon-stimulated gene 20 kDa protein	134.631524
Acan	Aggrecan core protein	134.816598
Hapln3	hyaluronan and proteoglycan link protein 3	134.851846
Mfge8	Lactadherin	134.87003
Abhd2	abhydrolase domain-containing protein 2	135.025144
Rlbp1	retinaldehyde-binding protein 1	135.122063
Polg	DNA polymerase subunit gamma-1	135.197016
rno-mir-9-3	rno-mir-9-3	135.254133
Rhcg	Ammonium transporter Rh type C	135.36142
Kif7	Uncharacterized protein	135.473063
Plin	Perilipin-1	135.497431
Pex11a	Peroxisomal membrane protein 11A	135.513735
Wdr93	WD repeat-containing protein 93	135.527818
Mesp1	mesoderm posterior protein 1	135.571777
Mesp2	mesoderm posterior protein 2	135.59002
Anpep	Aminopeptidase N	135.600751
LOC683402	similar to AP-3 complex subunit sigma-2 (Adapter-related p	135.653108
RGD1311021	RCG24599Uncharacterized protein	135.69698
Znf710	zinc finger protein 710	135.861678
Idh2	Isocitrate dehydrogenase	135.876058
Sema4b	semaphorin 4B	136.001235
Cib1	Calcium and integrin-binding protein 1	136.031865
NGRN_RAT	Neugrin	136.07546
Vps33b	Vacuolar protein sorting-associated protein 33B	136.085931
Prc1	protein regulator of cytokinesis 1	136.112674
Rccd1	Uncharacterized protein	136.134226
Unc45a	Protein unc-45 homolog A	136.143886
Hddc3	HD domain-containing protein 3	136.161425
Fes	tyrosine-protein kinase Fes/Fps	136.199523

Furin	Furin	136.209969
Iqgap1	ras GTPase-activating-like protein IQGAP1	136.552363
Crtc3	Uncharacterized protein	136.763502
Wdr73	WD repeat-containing protein 73	137.088348
Nmb	neuromedin-B	137.097463
Sec11a	Signal peptidase complex catalytic subunit SEC11A	137.109468
Znf592	zinc finger protein 592	137.188336
Alpk3	alpha-protein kinase 3	137.256805
Slc28a1	Sodium/nucleoside cotransporter 1	137.323995
Pde8a	high affinity cAMP-specific and IBMX-insensitive 3',5'-cyclic	137.415852
SNORA44	Small nucleolar RNA SNORA44	137.444624
LOC100365810	40S ribosomal protein S17	137.552232
Cpeb1	Cytoplasmic polyadenylation element-binding protein 1	137.556774
SCARNA15	Small Cajal body specific RNA 15	137.743986
Fsd2	fibronectin type III and SPRY domain-containing protein 2	137.749268
SNORA50	Small nucleolar RNA SNORA50	137.759109
Whamm	WASP homolog-associated protein with actin, membranes a	137.785814
Homer2	Homer protein homolog 2	137.827128
Fam103a1	family with sequence similarity 103, member A1 (Fam103a1	137.980239
RGD1305713	UPF0235 protein C15orf40 homolog	137.998479
Btbd1	BTB/POZ domain-containing protein 1	138.006567
Tm6sf1	transmembrane 6 superfamily member 1	138.058059
Hdgfrp3	Hepatoma-derived growth factor-related protein 3	138.098819
Sh3gl3	Endophilin-A3	138.398334
Fam154b	family with sequence similarity 154, member B (Fam154b),	138.905988
Eftud1	elongation factor Tu GTP binding domain containing 1	138.92062
SNORA15	Small nucleolar RNA SNORA15	138.942384
Tmc3	Uncharacterized protein	139.842574
Stard5	stAR-related lipid transfer protein 5	139.896272
Il16	pro-interleukin-16	139.908061
RGD1310371	similar to RIKEN cDNA 1700026D08 (RGD1310371), mRN	140.036328
Mesdc1	mesoderm development candidate 1	140.157974
Mesdc2	LDLR chaperone MESD	140.167851
RGD1305254	Uncharacterized protein	140.20304
Fam108c1	abhydrolase domain-containing protein FAM108C1	140.382431
Arnt2	Aryl hydrocarbon receptor nuclear translocator 2	140.535823
Zfand6	AN1-type zinc finger protein 6	140.885729
Vom2r40	vomeronasal 2 receptor, 40	141.602289
Vom2r41	vomeronasal 2 receptor 41	141.861806
Vom2r42	vomeronasal 2 receptor 42	142.010227
Vom2r43	vomeronasal 2 receptor 43	142.267794
Folh1	Glutamate carboxypeptidase 2	142.936379
Nox4	NADPH oxidase 4	143.415816
Tyr	tyrosinase	143.641257
U7	U7 small nuclear RNA	143.647155
Grm5	Metabotropic glutamate receptor 5	143.863506

Ctsc	Dipeptidyl peptidase 1Dipeptidyl peptidase 1 exclusion don	144.629802
Rab38	ras-related protein Rab-38	144.783909
Tmem135	Transmembrane protein 135	145.664797
Fzd4	Frizzled-4	145.953746
Prss23	Serine protease 23	146.081557
Eed	polycomb protein EED	146.563964
Picalm	Phosphatidylinositol-binding clathrin assembly protein	146.754045
Ccdc89	Coiled-coil domain-containing protein 89	147.091688
Crebzf	CREB/ATF bZIP transcription factor	147.124224
Tmem126a	Transmembrane protein 126A	147.129335
Tmem126b	transmembrane protein 126B	147.146187
Dlg2	Disks large homolog 2	148.051658
Ccdc90b	Coiled-coil domain-containing protein 90B, mitochondrial	149.410497
Ankrd42	ankyrin repeat domain 42	149.423846
Pcf11	Uncharacterized protein	149.475611
SNORA73	Small nucleolar RNA SNORA73 family	149.551354
Rab30	ras-related protein Rab-30	149.656373
RGD1559690	nitric oxide-inducible gene protein	149.685958
SNORA70	Small nucleolar RNA SNORA70	149.697952
Prcp	lysosomal Pro-X carboxypeptidase	149.711289
Fam181b	family with sequence similarity 181, member B (Fam181b), r	149.955641
U6	U6 spliceosomal RNA	152.647115
rno-mir-708	rno-mir-708	153.476669
Odz4	teneurin-4	153.808625
Nars2	probable asparaginyl-tRNA synthetase, mitochondrial	154.216134
Gab2	GRB2-associated-binding protein 2	154.348781
Kctd21	BTB/POZ domain-containing protein KCTD21	154.587686
Alg8	probable dolichyl pyrophosphate Glc1Man9GlcNAc2 alpha-	154.607442
Ndufc2	NADH dehydrogenase	154.635889
Thrsp	Thyroid hormone-inducible hepatic protein	154.647341
Kctd14	Uncharacterized protein	154.684785
RGD1561459	similar to RIKEN cDNA 1810020D17 (RGD1561459), mRN	154.786525
Clns1a	Methylosome subunit pICln	154.946673
Aqp11	Aquaporin-11	154.973798
Pak1	Serine/threonine-protein kinase PAK 1	155.057622
Capn5	Calpain-5	155.366703
B3gnt6	UDP-GlcNAc:betaGal beta-1,3-N-acetylglucosaminyltransfe	155.435459
Acer3	Uncharacterized protein	155.455466
Tsku	Tsukushin	155.61107
Lrrc32	leucine rich repeat containing 32	155.761334
Prkrir	52 kDa repressor of the inhibitor of the protein kinase	155.982782
Wnt11	wingless-type MMTV integration site family, member 11	156.126223
RGD1561870	similar to ribosomal protein L27 (RGD1561870), mRNA	156.142284
Dgat2	Diacylglycerol O-acyltransferase 2	156.447588
Mogat2	2-acylglycerol O-acyltransferase 2	156.516533
Map6	Microtubule-associated protein 6	156.592504

Serpinh1	Serpin H1	156.667042
B5DF39_RAT	Gdpd5 protein	156.703241
Klhl35	Uncharacterized protein	156.801077
Rps3	40S ribosomal protein S3	156.811473
SNORD15	Small nucleolar RNA SNORD15	156.812188
Arrb1	Beta-arrestin-1	156.871562
rno-mir-326	rno-mir-326	156.887513
Slco2b1	Solute carrier organic anion transporter family member 2B1	156.992493
Q5RJS1_RAT	Olr35 protein	157.074498
Neu3	Sialidase-3	157.172548
Spcs2	signal peptidase complex subunit 2 homolog	157.199447
SNORA20	Small nucleolar RNA SNORA20	157.319772
LOC100360934	chordin-like protein 2	157.391482
Pold3	DNA polymerase delta subunit 3	157.453059
Lipt2	lipoyl(octanoyl) transferase 2 (putative)	157.538704
Kcne3	Potassium voltage-gated channel subfamily E member 3	157.558044
Pgm2l1	glucose 1,6-bisphosphate synthase	157.605906
P4ha3	Prolyl 4-hydroxylase subunit alpha-3	157.663338
Ppme1	protein phosphatase methylesterase 1	157.725633
Ucp3	Mitochondrial uncoupling protein 3	157.896001
Ucp2	Mitochondrial uncoupling protein 2	157.922179
Dnajb13	dnaJ homolog subfamily B member 13	157.931501
Chchd8	coiled-coil-helix-coiled-coil-helix domain-containing protein	157.965119
Mrpl48	39S ribosomal protein L48, mitochondrial	157.979818
Rab6a	Ras-related protein Rab-6A	158.041088
SNORA4	Small nucleolar RNA SNORA4	158.048843
Plekhb1	Pleckstrin homology domain-containing family B member 1	158.08193
Fam168a	family with sequence similarity 168, member A (Fam168a),	158.140473
Relt	tumor necrosis factor receptor superfamily member 19L	158.293361
P2ry6	P2Y purinoceptor 6	158.381128
P2ry2	P2Y purinoceptor 2	158.440062
Fchsd2	FCH and double SH3 domains protein 2	158.54883
Q6QI52_RAT	LRRGT00156	158.590389
LOC100364491	ribosomal protein L7a (Rpl7a), mRNA	158.694172
Atg16l2	ATG16 autophagy related 16-like 2	158.787779
Stard10	PCTP-like protein	158.81997
Pde2a	cGMP-dependent 3',5'-cyclic phosphodiesterase	158.921607
rno-mir-139	rno-mir-139	158.976576
Art2	T-cell ecto-ADP-ribosyltransferase 1	159.055461
Clpb	Caseinolytic peptidase B protein homolog	159.126512
5S_rRNA	5S ribosomal RNA	159.2668
Phox2a	Paired mesoderm homeobox protein 2A	159.273524
Inppl1	Phosphatidylinositol-3,4,5-trisphosphate 5-phosphatase 2	159.27813
Folr2	folate receptor beta	159.295773
Folr1	folate receptor alpha	159.315192
RGD1311634	similar to RIKEN cDNA 3200002M19 (RGD1311634), mRN	159.35911

Lrtomt	Transmembrane O-methyltransferase	159.36201
MGC72560	Ragulator complex protein LAMTOR1	159.367471
Lrrc51	Leucine-rich repeat-containing protein 51	159.373973
Il18bp	interleukin-18-binding protein	159.471809
Rnf121	RING finger protein 121	159.475046
TRPC2_RAT	Short transient receptor potential channel 2	159.54282
Art5	Ecto-ADP-ribosyltransferase 5	159.576991
Art1	GPI-linked NAD(P)(+)--arginine ADP-ribosyltransferase 1	159.586246
Chrna10	Neuronal acetylcholine receptor subunit alpha-10	159.590904
Nup98	Nuclear pore complex protein Nup98-Nup96Nuclear pore c	159.598048
Pgap2	Post-GPI attachment to proteins factor 2	159.69692
Rhog	rho-related GTP-binding protein RhoG	159.724016
Stim1	Stromal interaction molecule 1	159.761567
Trim21	52 kDa Ro protein	160.175882
LOC293190	bcl-2-like protein 1 isoform 3	160.254067
SNORA2	Small nucleolar RNA SNORA2/SNORA34 family	161.283594
LOC689064	Hemoglobin subunit beta-2	161.578128
Hbb	Hemoglobin subunit beta-1	161.584858
Hbb-b1	hemoglobin, beta adult major chain	161.590671
Hbg1	hemoglobin, gamma A	161.64024
Hbe2	hemoglobin, epsilon 2	161.647651
Hbe1	hemoglobin, epsilon 1	161.651306
LOC499219	LRRG00121	161.788384
RGD1562433	similar to ubiquilin 1 isoform 2 (RGD1562433), mRNA	161.962308
Ubqln3	ubiquilin-3	161.974585
Ubqlnl	Ubiquilin-like protein	161.985144
RGD1310717	similar to RIKEN cDNA E030002O03 (RGD1310717), mRN	161.990223
Trim6	tripartite motif-containing 6	162.066157
Trim5	tripartite motif-containing protein 5	162.096454
Trim30	Uncharacterized protein	162.173307
U4	U4 spliceosomal RNA	162.457008
Fam160a2	FTS and Hook-interacting protein	163.110928
Cnga4	Cyclic nucleotide-gated cation channel alpha-4	163.137544
Cckbr	Gastrin/cholecystokinin type B receptor	163.156914
Prkcdbp	Protein kinase C delta-binding protein	163.212922
Smpd1	sphingomyelin phosphodiesterase	163.27897
Apbb1	Amyloid beta A4 precursor protein-binding family B membe	163.282918
Hpx	Hemopexin	163.319509
Trim3	Tripartite motif-containing protein 3	163.331603
Arfip2	Arfaptin-2	163.364861
TIM9B_RAT	Mitochondrial import inner membrane translocase subunit T	163.369752
Dnhd1	Uncharacterized protein	163.462348
Rrp8	Ribosomal RNA-processing protein 8	163.47703
Ilk	Integrin-linked protein kinase	163.481299
Taf10	TAF10 RNA polymerase II, TATA box binding protein (TBP)-	163.487518
Tpp1	Tripeptidyl-peptidase 1	163.490393

Dchs1	dachsous 1	163.49734
RM17_RAT	39S ribosomal protein L17, mitochondrial	163.554719
Syt9	Synaptotagmin-9	164.722168
Olfml1	Olfactomedin-like protein 1	164.953064
Ppfibp2	protein tyrosine phosphatase, receptor-type, F interacting pr	164.979709
NB5R2_RAT	NADH-cytochrome b5 reductase 2	165.131108
LOC499235	LRRGT00141	165.181656
Nlrp10	NACHT, LRR and PYD domains-containing protein 10	166.411647
Ric3	resistance to inhibitors of cholinesterase 3 homolog	166.494113
Lmo1	LIM domain only protein 3	166.706026
snoU13	Small nucleolar RNA U13	167.002996
Trim66	tripartite motif-containing protein 66	167.04954
Rpl27a	60S ribosomal protein L27a	167.115389
SNORA3	Small nucleolar RNA SNORA3/SNORA45 family	167.117372
St5	suppression of tumorigenicity 5 protein	167.119711
RGD1306959	similar to C11orf17 protein (RGD1306959), mRNA	167.324919
LOC499240	similar to predicted gene ICRFP703B1614Q5.5 (LOC49924	167.333129
Tmem9b	transmembrane protein 9B	167.355899
Nrip3	nuclear receptor-interacting protein 3	167.380328
Dennd5a	DENN/MADD domain containing 5A	167.519649
Tmem41b	Transmembrane protein 41B	167.608205
Ipo7	importin-7	167.658312
SNORA23	Small nucleolar RNA SNORA23	167.68657
Zfp143	Zinc finger protein 143	167.704313
Wee1	Wee1-like protein kinase	167.769797
Swap70	switch-associated protein 70	167.888783
RGD1559442	SET binding factor 2	167.974268
Adm	ADMAdrenomedullinProadrenomedullin N-20 terminal pept	168.380255
Ampd3	AMP deaminase 3	168.533491
rnf141	RING finger protein 141	168.572375
Lyve1	lymphatic vessel endothelial hyaluronic acid receptor 1	168.60146
Mrvi1	protein MRVI1 isoform b	168.627363
Ctr9	Ctr9, Paf1/RNA polymerase II complex component, homolog	168.784377
snoU97	Small nucleolar RNA U97	168.834306
E9PSI9_RAT	putative polypeptide N-acetylgalactosaminyltransferase-like	169.251525
Usp47	ubiquitin specific peptidase 47	169.836253
Dkk3	dickkopf-related protein 3	169.924618
Mical2	protein MICAL-2	170.076006
Micalcl	MICAL C-terminal-like protein	170.178141
Parva	Alpha-parvin	170.232441
Tead1	transcriptional enhancer factor TEF-1	170.557393
LOC687311	NADH dehydrogenase	170.580048
Arntl	Aryl hydrocarbon receptor nuclear translocator-like protein 1	171.062181
Btbd10	BTB/POZ domain-containing protein 10	171.163522
Pth	Parathyroid hormone	171.240596
Far1	Fatty acyl-CoA reductase 1	171.392301

Spon1	Spondin-1	171.70072
Rras2	ras-related protein R-Ras2	172.026263
Copb1	Coatomer subunit beta	172.199677
Psma1	Proteasome subunit alpha type-1	172.237763
Pde3b	cGMP-inhibited 3',5'-cyclic phosphodiesterase B	172.409282
B5DFA4_RAT	vitamin D 25-hydroxylase	172.593057
Calca	Calcitonin	172.68617
Calcb	Calcitonin gene-related peptide 2	172.777347
Insc	inscuteable homolog (Drosophila) (Insc), mRNA	172.804412
7SK	7SK RNA	173.002191
Sox6	transcription factor SOX-6	173.556967
RGD1311703	small acidic protein	174.173969
Plekha7	pleckstrin homology domain containing, family A member 7	174.204979
Rps13	40S ribosomal protein S13	174.425816
SNORD14	Small nucleolar RNA SNORD14	174.427277
Pik3c2a	phosphatidylinositol-4-phosphate 3-kinase C2 domain-cont	174.430831
Nucb2	Nucleobindin-2	174.605677
SNORA17	Small nucleolar RNA SNORA17	175.033994
YBOX1_RAT	Nuclease-sensitive element-binding protein 1	175.17944
Xylt1	Xylosyltransferase 1	175.68736
Rps15a	40S ribosomal protein S15a	176.321901
Arl6ip1	ADP-ribosylation factor-like protein 6-interacting protein 1	176.332758
SYT17_RAT	Synaptotagmin-17	176.599608
Itpripl2	inositol 1,4,5-triphosphate receptor-interacting protein-like 2	176.70669
Coq7	Ubiquinone biosynthesis protein COQ7 homolog	176.741638
Tmc7	transmembrane channel-like protein 7	176.761053
Tmc5	Transmembrane channel-like protein 5	176.84398
Cp110	centrosomal protein of 110 kDa	176.937287
LOC361635	UPF0505 protein C16orf62 homolog	176.965214
SNORA24	Small nucleolar RNA SNORA24	177.060142
RGD1565096	similar to TSG118.1 (RGD1565096), mRNA	177.084712
Gprc5b	G-protein coupled receptor family C group 5 member B	177.205905

Supplementary table 1. List of genes within the QTL region on chromosome 1

Supplementary Table 2: Candidate genes in the QTL region previously reported to be associated with psychiatric and behavioral disorders.

Gene symbol	Gene description	Gene location (Mbp)	Associated disorder
Rgma	Repulsive guidance molecule A	128.547399	Depression[1]
Chd2	Chromodomain-helicase-DNA-binding protein 2	128.609727	Autism[2]
St8sia2	Alpha-2,8-sialyltransferease 8B	129.158714	Schizophrenia[3]; Bipolar[3]; Autism[4]
Sv2b	Synaptic vesicle glycoprotein 2B	130.683153	Depression[1]
Homer2	Homer protein homolog 2	137.827128	Schizophrenia[5]
Folh1	Glutamate carboxypeptidase 2	142.936379	Depression[6]; Schizophrenia[7]
Tyr	Tyrosinase	143.641257	Bipolar[8]
Grm5	Metabotropic glutamate receptor 5	143.863506	Schizophrenia[9]; ADHD[10]; Addiction[11]; Autism[12]
Dlg2	Disks large homolog 2	148.051658	Schizophrenia[13]; Autism[14]
Odz4	Teneurin-4	153.808625	Bipolar[15]; Schizophrenia[16]
Arrb1	Beta-arrestin-1	156.871562	Depression[17]; Addiction[18]
Plekhb1	Pleckstrin homology domain-containing family B membrane 1	158.08193	ADHD[19]
Folr2	Folate receptor beta	159.295773	Autism[20]
Chrna10	Neuronal acetylcholine receptor subunit alpha-10	159.590904	Addiction[21]
Hbb	Hemoglobin subunit beta-1	161.584858	Depression[22]
Cckbr	Gastrin/cholecystokinin type B receptor	163.156914	Anxiety[23]; Suicide[24]

Ilk	Integrin-linked protein kinase	163.481299	Addiction [25]
Ric3	Resistance to inhibitors of cholinesterase 3 homolog	166.494113	Bipolar[26]; Schizophrenia[26]
Lmo1	LIM domain only protein 3	166.706026	Addiction[27]
Rps15a	40S ribosomal protein S15a	176.321901	Anxeity[28]

1. Verma R, Holmans P, Knowles JA, Grover D, Evgrafov OV, Crowe RR *et al.* Linkage disequilibrium mapping of a chromosome 15q25-26 major depression linkage region and sequencing of NTRK3. *Biological psychiatry* 2008; **63**(12)**:** 1185-1189.

2. Wilkinson B, Grepo N, Thompson BL, Kim J, Wang K, Evgrafov OV *et al.* The autism-associated gene chromodomain helicase DNA-binding protein 8 (CHD8) regulates noncoding RNAs and autism-related genes. *Translational psychiatry* 2015; **5:** e568.

3. Vazza G, Bertolin C, Scudellaro E, Vettori A, Boaretto F, Rampinelli S *et al.* Genome-wide scan supports the existence of a susceptibility locus for schizophrenia and bipolar disorder on chromosome 15q26. *Molecular psychiatry* 2007; **12**(1)**:** 87-93.

4. Anney R, Klei L, Pinto D, Regan R, Conroy J, Magalhaes TR *et al.* A genome-wide scan for common alleles affecting risk for autism. *Human molecular genetics* 2010; **19**(20)**:** 4072-4082.

5. Gilks WP, Allott EH, Donohoe G, Cummings E, International Schizophrenia C, Gill M *et al.* Replicated genetic evidence supports a role for HOMER2 in schizophrenia. *Neuroscience letters* 2010; **468**(3)**:** 229-233.

6. Ye X, Lai CQ, Crott JW, Troen AM, Ordovas JM, Tucker KL. The folate hydrolase 1561C>T polymorphism is associated with depressive symptoms in Puerto Rican adults. *Psychosomatic medicine* 2011; **73**(5)**:** 385-392.

7. Roffman JL, Brohawn DG, Nitenson AZ, Macklin EA, Smoller JW, Goff DC. Genetic variation throughout the folate metabolic pathway influences negative symptom severity in schizophrenia. *Schizophrenia bulletin* 2013; **39**(2)**:** 330-338.

8. De bruyn A, Mendelbaum K, Sandkuijl LA, Delvenne V, Hirsch D, Staner L *et al.* Nonlinkage of bipolar illness to tyrosine hydroxylase, tyrosinase, and D2 and D4 dopamine receptor genes on chromosome 11. *The American journal of psychiatry* 1994; **151**(1)**:** 102-106.

9. Shibata H, Joo A, Fujii Y, Tani A, Makino C, Hirata N *et al.* Association study of polymorphisms in the GluR5 kainate receptor gene (GRIK1) with schizophrenia. *Psychiatric genetics* 2001; **11**(3)**:** 139-144.

10. Elia J, Gai X, Xie HM, Perin JC, Geiger E, Glessner JT *et al.* Rare structural variants found in attention-deficit hyperactivity disorder are preferentially associated with neurodevelopmental genes. *Molecular psychiatry* 2010; **15**(6)**:** 637-646.

11. Meyers JL, Salling MC, Almli LM, Ratanatharathorn A, Uddin M, Galea S *et al.* Frequency of alcohol consumption in humans; the role of metabotropic glutamate receptors and downstream signaling pathways. *Translational psychiatry* 2015; **5:** e586.

12. Fatemi SH, Folsom TD. GABA receptor subunit distribution and FMRP-mGluR5 signaling abnormalities in the cerebellum of subjects with schizophrenia, mood disorders, and autism. *Schizophrenia research* 2015; **167**(1-3)**:** 42-56.

13. Ingason A, Giegling I, Hartmann AM, Genius J, Konte B, Friedl M *et al.* Expression analysis in a rat psychosis model identifies novel candidate genes validated in a large case-control sample of schizophrenia. *Translational psychiatry* 2015; **5:** e656.

14. Egger G, Roetzer KM, Noor A, Lionel AC, Mahmood H, Schwarzbraun T *et al.* Identification of risk genes for autism spectrum disorder through copy number variation analysis in Austrian families. *Neurogenetics* 2014; **15**(2)**:** 117-127.

15. Psychiatric GCBDWG. Large-scale genome-wide association analysis of bipolar disorder identifies a new susceptibility locus near ODZ4. *Nature genetics* 2011; **43**(10)**:** 977-983.

16. Ivorra JL, Rivero O, Costas J, Iniesta R, Arrojo M, Ramos-Rios R *et al.* Replication of previous genome-wide association studies of psychiatric diseases in a large schizophrenia case-control sample from Spain. *Schizophrenia research* 2014; **159**(1)**:** 107-113.

17. Chang HS, Won ES, Lee HY, Ham BJ, Kim YG, Lee MS. Association of ARRB1 polymorphisms with the risk of major depressive disorder and with treatment response to mirtazapine. *Journal of psychopharmacology* 2015; **29**(5)**:** 615-622.

18. Sun D, Ma JZ, Payne TJ, Li MD. Beta-arrestins 1 and 2 are associated with nicotine dependence in European American smokers. *Molecular psychiatry* 2008; **13**(4)**:** 398-406.

19. Lesch KP, Selch S, Renner TJ, Jacob C, Nguyen TT, Hahn T *et al.* Genome-wide copy number variation analysis in attention-deficit/hyperactivity disorder: association with neuropeptide Y gene dosage in an extended pedigree. *Molecular psychiatry* 2011; **16**(5)**:** 491-503.

20. Schmidt RJ, Hansen RL, Hartiala J, Allayee H, Schmidt LC, Tancredi DJ *et al.* Prenatal vitamins, one-carbon metabolism gene variants, and risk for autism. *Epidemiology* 2011; **22**(4)**:** 476-485.

21. Ehringer MA, McQueen MB, Hoft NR, Saccone NL, Stitzel JA, Wang JC *et al.* Association of CHRN genes with "dizziness" to tobacco. *American journal of medical genetics Part B, Neuropsychiatric genetics : the official publication of the International Society of Psychiatric Genetics* 2010; **153B**(2)**:** 600-609.

22. Yamamoto Y, Ueyama T, Ito T, Tsuruo Y. Downregulation of growth hormone 1 gene in the cerebellum and prefrontal cortex of rats with depressive-like behavior. *Physiological genomics* 2015; **47**(5)**:** 170-176.

23. Le-Niculescu H, Balaraman Y, Patel SD, Ayalew M, Gupta J, Kuczenski R *et al.* Convergent functional genomics of anxiety disorders: translational identification of genes, biomarkers, pathways and mechanisms. *Translational psychiatry* 2011; **1:** e9.

24. Sears C, Wilson J, Fitches A. Investigating the role of BDNF and CCK system genes in suicidality in a familial bipolar cohort. *Journal of affective disorders* 2013; **151**(2)**:** 611-617.

25. Chen Q, Xiong X, Lee TH, Liu Y, Wetsel WC, Zhang X. Neural plasticity and addiction: integrin-linked kinase and cocaine behavioral sensitization. *Journal of neurochemistry* 2008; **107**(3)**:** 679-689.

26. Severance EG, Yolken RH. Lack of RIC-3 congruence with beta2 subunit-containing nicotinic acetylcholine receptors in bipolar disorder. *Neuroscience* 2007; **148**(2)**:** 454-460.

27. Kapoor M, Wang JC, Wetherill L, Le N, Bertelsen S, Hinrichs AL *et al.* A meta-analysis of two genome-wide association studies to identify novel loci for maximum number of alcoholic drinks. *Human genetics* 2013; **132**(10)**:** 1141-1151.

28. Park CC, Gale GD, de Jong S, Ghazalpour A, Bennett BJ, Farber CR *et al.* Gene networks associated with conditional fear in mice identified using a systems genetics approach. *BMC systems biology* 2011; **5:** 43.

Gene Name	SNP ID	Chromosome	Position(Mbp)	Reference Allele	Alternative Allele	Location
Chd2	1_128649115_G/C	1	128.649115	G	C	INTRONIC
Sv2b	1_130835497_C/A	1	130.835497	C	A	INTRONIC
Sv2b	1_130837654_G/A	1	130.837654	G	A	INTRONIC
Akap13	1_131315604_G/A	1	131.315604	G	A	INTRONIC
Ntrk3	1_134268648_G/T	1	134.268648	G	T	INTRONIC
Acan	1_134840489_T/C	1	134.840489	T	C	INTRONIC
Abhd2	1_135088391_G/C	1	135.088391	G	C	INTRONIC
Iqgap1	1_136631739_C/T	1	136.631739	C	T	INTRONIC
Pde8a	1_137547830_C/T	1	137.54783	C	T	DOWNSTREAM
Cpeb1	1_137582309_A/G	1	137.582309	A	G	INTRONIC
Cpeb1	1_137609354_C/G	1	137.609354	C	G	INTRONIC
Homer2	1_137882265_A/G	1	137.882265	A	G	INTRONIC
Homer2	1_137882278_A/G	1	137.882278	A	G	INTRONIC
Homer2	1_137882369_A/G	1	137.882369	A	G	INTRONIC
Homer2	1_137882379_A/G	1	137.882379	A	G	INTRONIC
Tm6sf1	1_138079519_A/G	1	138.079519	A	G	INTRONIC
Sh3gl3	1_138430747_A/G	1	138.430747	A	G	INTRONIC
Sh3gl3	1_138451888_C/G	1	138.451888	C	G	INTRONIC
Sh3gl3	1_138512033_C/T	1	138.512033	C	T	INTRONIC
Arnt2	1_140531141_C/A	1	140.531141	C	A	DOWNSTREAM
Arnt2	1_140531145_C/A	1	140.531145	C	A	DOWNSTREAM
Arnt2	1_140651827_A/G	1	140.651827	A	G	INTRONIC
Olr11	1_141494096_C/A	1	141.494096	C	A	UPSTREAM
Vom2r40	1_141659141_T/A	1	141.659141	T	A	INTRONIC
Vom2r40	1_141659143_T/G	1	141.659143	T	G	INTRONIC
Folh1	1_142966763_G/T	1	142.966763	G	T	INTRONIC
Folh1	1_142966968_G/A	1	142.966968	G	A	INTRONIC
Grm5	1_143859713_A/T	1	143.859713	A	T	UPSTREAM
Grm5	1_144005117_T/C	1	144.005117	T	C	INTRONIC
Grm5	1_144032182_A/T	1	144.032182	A	T	INTRONIC
Dlg2	1_148421815_C/A	1	148.421815	C	A	INTRONIC
Dlg2	1_148443210_A/T	1	148.44321	A	T	INTRONIC
Dlg2	1_148476775_A/G	1	148.476775	A	G	INTRONIC
Dlg2	1_148502976_G/A	1	148.502976	G	A	INTRONIC
Dlg2	1_148562440_A/G	1	148.56244	A	G	INTRONIC
Dlg2	1_149203589_A/G	1	149.203589	A	G	INTRONIC
Dlg2	1_149203594_A/G	1	149.203594	A	G	INTRONIC
Odz4	1_153937181_G/A	1	153.937181	G	A	INTRONIC
Nars2	1_154235371_C/G	1	154.235371	C	G	INTRONIC
Aqp11	1_154978188_G/A	1	154.978188	G	A	INTRONIC
Fchsd2	1_158557777_G/A	1	158.557777	G	A	INTRONIC
Pde2a	1_158930569_A/G	1	158.930569	A	G	INTRONIC
Pde2a	1_158930585_T/C	1	158.930585	T	C	INTRONIC
Olr119	1_161435537_T/C	1	161.435537	T	C	UPSTREAM
Hbb	1_161590423_T/A	1	161.590423	T	A	UPSTREAM
Ppfibp2	1_165008099_C/G	1	165.008099	C	G	INTRONIC
Olr237	1_165247608_T/C	1	165.247608	T	C	SYNONYMOUS_CODING
LOC690767	1_166068612_C/T	1	166.068612	C	T	NON_SYNONYMOUS_CODING
Ric3	1_166523757_T/C	1	166.523757	T	C	INTRONIC
St5	1_167138083_A/G	1	167.138083	A	G	INTRONIC
Mrvi1	1_168714374_A/G	1	168.714374	A	G	INTRONIC
Spon1	1_171742726_T/C	1	171.742726	T	C	INTRONIC
Spon1	1_171777726_C/G	1	171.777726	C	G	INTRONIC
Spon1	1_171904938_G/T	1	171.904938	G	T	INTRONIC
Spon1	1_171904941_G/A	1	171.904941	G	A	INTRONIC
Spon1	1_171998337_T/C	1	171.998337	T	C	INTRONIC
Pde3b	1_172463519_T/C	1	172.463519	T	C	INTRONIC

Supplementary table 3. List of genes in the QTL region that present SNPs

Gene Name	INDEL ID	Chromosome	Start(Mbp)	End(Mbp)	Reference Allele	Alternative Allele	Location
F1M810_RAT	1_129608899_TG/-	1	129.6089	129.6089	TG	-	INTRONIC
Sv2b	1_130739698_-/CA	1	130.7397	130.7397	-	CA	INTRONIC
Sv2b	1_130742599_-/AC	1	130.7426	130.7426	-	AC	INTRONIC
Sv2b	1_130826672_-/GT	1	130.82667	130.82667	-	GT	INTRONIC
F1M811_RAT	1_131129015_-/T	1	131129015	131129016	-	T	INTRONIC
F1M811_RAT	1_131134284_T/-	1	131134284	131134284	T	-	INTRONIC
Klhl25	1_131540432_TG/-	1	131540432	131540433	TG	-	UPSTREAM
F1M814_RAT	1_132456191_-/CA	1	132.45619	132.45619	-	CA	INTRONIC
F1M814_RAT	1_132675878_-/TG	1	132.67588	132.67588	-	TG	INTRONIC
Ntrk3	1_134035794_-/AC	1	134.03579	134.0358	-	AC	INTRONIC
Mrps11	1_134518244_-/CCACACA	1	134518244	134518245	-	CCACACA	DOWNSTREAM
Isg20	1_134634753_G/-	1	134634753	134634753	G	-	INTRONIC
Mfge8	1_134871094_-/C	1	134.87109	134.8711	-	C	INTRONIC
Abhd2	1_135021727_-/G	1	135021727	135021728	-	G	UPSTREAM
Abhd2	1_135029169_-/A	1	135029169	135029170	-	A	INTRONIC
Rlbp1	1_135138240_-/TC	1	135.13824	135.13824	-	TC	UPSTREAM
Rhcg	1_135362995_GAGAGAGAGA/-	1	135362995	135363004	GAGAGAGAGA	-	SPLICE_SITE,INTRONIC
Wdr93	1_135549549_-/T	1	135549549	135549550	-	T	INTRONIC
Wdr93	1_135549838_-/CA	1	135549838	135549839	-	CA	INTRONIC
Anpep	1_135598576_-/A	1	135598576	135598577	-	A	DOWNSTREAM
Idh2	1_135899068_-/A	1	135899068	135899069	-	A	UPSTREAM
D4A0Q8_RAT	1_136069127_-/A	1	136069127	136069128	-	A	INTRONIC
Prc1	1_136135140_T/-	1	136135140	136135140	T	-	DOWNSTREAM
D4A4J3_RAT	1_136167005_A/-	1	136167005	136167005	A	-	DOWNSTREAM
Iqgap1	1_136558664_-/CC	1	136.55866	136.55867	-	CC	INTRONIC
Iqgap1	1_136641407_-/A	1	136641407	136641408	-	A	INTRONIC
Alpk3	1_137269970_GTGTGTGTGTGTGT/-	1	137.26997	137.26998	GTGTGTGTGTGTGT	-	INTRONIC
Slc28a1	1_137334547_-/TG	1	137.33455	137.33455	-	TG	INTRONIC
Homer2	1_137882093_-/C	1	137.88209	137.88209	-	C	INTRONIC
RGD1305713	1_138003252_-/GT	1	138.00325	138.00325	-	GT	INTRONIC
Sh3gl3	1_138533714_AGAGAG/-	1	138.53371	138.53372	AGAGAG	-	DOWNSTREAM
F1M767_RAT	1_138654884_TCTCTCTCTC/-	1	138.65488	138.65489	TCTCTCTCTC	-	INTRONIC
F1M767_RAT	1_138713216_-/AC	1	138.71322	138.71322	-	AC	INTRONIC
D3ZFL6_RAT	1_139143140_-/GT	1	139.14314	139.14314	-	GT	DOWNSTREAM
Tmc3	1_139861479_-/TC	1	139.86148	139.86148	-	TC	INTRONIC
Arnt2	1_140671403_AC/-	1	140.6714	140.6714	AC	-	INTRONIC
FAAA_RAT	1_140858554_TC/-	1	140.85855	140.85856	TC	-	INTRONIC
Nox4	1_143476690_T/-	1	143476690	143476690	T	-	INTRONIC
Nox4	1_143480318_-/TCTA	1	143.48032	143.48032	-	TCTA	INTRONIC
Nox4	1_143599429_-/AT	1	143.59943	143.59943	-	AT	INTRONIC
Grm5	1_143897429_-/A	1	143897429	143897430	-	A	INTRONIC
Grm5	1_144083448_-/TA	1	144.08345	144.08345	-	TA	INTRONIC
F1LUQ4_RAT	1_146362231_AC/-	1	146.36223	146.36223	AC	-	INTRONIC
LOC680270	1_146899406_-/CAATCA	1	146.89941	146.89941	-	CAATCA	INTRONIC
Dlg2	1_148190578_-/CA	1	148190578	148190579	-	CA	INTRONIC
Dlg2	1_148347335_-/A	1	148.34734	148.34734	-	A	INTRONIC
Dlg2	1_148476682_-/TA	1	148.47668	148.47668	-	TA	INTRONIC
Dlg2	1_148487662_AG/-	1	148.48766	148.48766	AG	-	INTRONIC
Dlg2	1_148487707_GT/-	1	148.48771	148.48771	GT	-	INTRONIC
Dlg2	1_148539160_-/T	1	148539160	148539161	-	T	INTRONIC
Dlg2	1_148883316_CT/-	1	148.88332	148.88332	CT	-	INTRONIC
Dlg2	1_148988102_ATATATATATATATAT/-	1	148.9881	148.98812	ATATATATATATATAT	-	INTRONIC
Rab30	1_149659105_-/TG	1	149.65911	149.65911	-	TG	INTRONIC
RGD1559690	1_149703024_A/-	1	149703024	149703024	A	-	INTRONIC
Odz4	1_153843731_GT/-	1	153.84373	153.84373	GT	-	INTRONIC
Nars2	1_154271062_-/ACAGAC	1	154.27106	154.27106	-	ACAGAC	INTRONIC
Nars2	1_154284900_T/-	1	154284900	154284900	T	-	INTRONIC
Gab2	1_154478186_-/CACACACAC	1	154478186	154478187	-	CACACACAC	INTRONIC
D3ZZQ6_RAT	1_154718553_-/G	1	154.71855	154.71855	-	G	INTRONIC
D3ZGQ8_RAT	1_154823313_-/A	1	154823313	154823314	-	A	INTRONIC
D3ZKE1_RAT	1_156295478_-/GT	1	156.29548	156.29548	-	GT	INTRONIC
D3ZKE1_RAT	1_156328047_-/TCTCTCTCTC	1	156.32805	156.32805	-	TCTCTCTCTC	INTRONIC
Map6	1_156638174_-/A	1	156638174	156638175	-	A	INTRONIC
B5DF39_RAT	1_156738188_-/GT	1	156.73819	156.73819	-	GT	INTRONIC
Lipt2	1_157537693_T/-	1	157537693	157537693	T	-	UPSTREAM
Fam168a	1_158235825_-/CT	1	158.23583	158.23583	-	CT	INTRONIC
D4ADZ1_RAT	1_158314584_A/-	1	158314584	158314584	A	-	DOWNSTREAM
Fchsd2	1_158582535_C/-	1	158582535	158582535	C	-	INTRONIC
Fchsd2	1_158755377_-/A	1	158755377	158755378	-	A	INTRONIC
F1LM60_RAT	1_158878464_TT/-	1	158878464	158878465	TT	-	INTRONIC
Il18bp	1_159477723_-/C	1	159477723	159477724	-	C	UPSTREAM
Rnf121	1_159492697_-/AC	1	159.4927	159.4927	-	AC	INTRONIC
Nup98	1_159629524_A/-	1	159629524	159629524	A	-	INTRONIC
Nup98	1_159631695_-/T	1	159631695	159631696	-	T	INTRONIC
Stim1	1_159764629_-/AGAGAGAGAG	1	159.76463	159.76463	-	AGAGAGAGAG	INTRONIC
Stim1	1_159884343_AC/-	1	159.88434	159.88434	AC	-	INTRONIC
Olr111	1_161323284_-/CG	1	161323284	161323285	-	CG	DOWNSTREAM
Olr135	1_161762982_T/-	1	161762982	161762982	T	-	DOWNSTREAM
Trim6	1_162061328_-/A	1	162061328	162061329	-	A	UPSTREAM
Olr154	1_162220333_-/GA	1	162220333	162220334	-	GA	UPSTREAM
Olr154	1_162223980_TG/-	1	162.22398	162.22398	TG	-	UPSTREAM
Olr168	1_162489797_-/G	1	162.4898	162.4898	-	G	DOWNSTREAM

Olr180	1_162735896_A/-	1	162735896	162735896	A	-	DOWNSTREAM
Olr180	1_162735898_A/-	1	162735898	162735898	A	-	DOWNSTREAM
LOC100362148	1_162868053_-/T	1	162868053	162868054	-	T	UPSTREAM
Olr193	1_162893376_-/A	1	162893376	162893377	-	A	DOWNSTREAM
Olr193	1_162901538_GT/-	1	162.90154	162.90154	GT	-	UPSTREAM
Olr194	1_162932668_C/-	1	162.93267	162.93267	C	-	UPSTREAM
Olr201	1_163075087_-/AC	1	163.07509	163.07509	-	AC	UPSTREAM
Cckbr	1_163157222_C/-	1	163157222	163157222	C	-	INTRONIC
Apbb1	1_163297794_TT/-	1	163297794	163297795	TT	-	INTRONIC
F1LZ18_RAT	1_163701435_TT/-	1	163.70144	163.70144	TT	-	UPSTREAM
Olr229	1_164358981_C/-	1	164358981	164358981	C	-	DOWNSTREAM
Syt9	1_164770760_T/-	1	164770760	164770760	T	-	INTRONIC
Ppfibp2	1_165047610_-/GTGT	1	165.04761	165.04761	-	GTGT	INTRONIC
Ppfibp2	1_165076240_TACACACACACA/-	1	165.07624	165.07625	TACACACACACA	-	INTRONIC
D4A7R5_RAT	1_165190282_-/AGGAAGGAAGGAAGG	1	165.19028	165.19028	-	AGGAAGGAAGGAAGG	DOWNSTREAM
Olr239	1_165268528_T/-	1	165268528	165268528	T	-	UPSTREAM
Olr245	1_165438665_-/GTGTGT	1	165438665	165438666	-	GTGTGT	DOWNSTREAM
Olr260	1_165809567_AAAGA/-	1	165.80957	165.80957	AAAGA	-	DOWNSTREAM
Olr262	1_165823727_ACATGC/-	1	165823727	165823732	ACATGC	-	UPSTREAM
Olr281	1_166472403_G/-	1	166472403	166472403	G	-	FRAMESHIFT_CODING
Olr282	1_166487497_GTGTGTGTGTGTGTGTGT/-	1	166487497	166487514	GTGTGTGTGTGTGTGTGT	-	DOWNSTREAM
Ric3	1_166648228_TCTCTCTCTC/-	1	166.64823	166.64824	TCTCTCTCTC	-	INTRONIC
Lmo1	1_166702862_T/-	1	166702862	166702862	T	-	DOWNSTREAM
Lmo1	1_166729026_-/CA	1	166.72903	166.72903	-	CA	INTRONIC
Tmem9b	1_167367043_-/TC	1	167367043	167367044	-	TC	INTRONIC
Swap70	1_167911659_-/G	1	167911659	167911660	-	G	INTRONIC
Swap70	1_167927999_C/-	1	167927999	167927999	C	-	INTRONIC
F1M683_RAT	1_168065753_T/-	1	168065753	168065753	T	-	INTRONIC
F1M683_RAT	1_168115964_T/-	1	168115964	168115964	T	-	INTRONIC
F1M683_RAT	1_168147706_-/A	1	168147706	168147707	-	A	INTRONIC
E9PSI9_RAT	1_169263659_A/-	1	169263659	169263659	A	-	INTRONIC
E9PSI9_RAT	1_169346363_-/AC	1	169.34636	169.34636	-	AC	INTRONIC
E9PSI9_RAT	1_169368882_-/C	1	169.36888	169.36888	-	C	INTRONIC
Usp47	1_169891406_-/A	1	169891406	169891407	-	A	INTRONIC
Mical2	1_170117853_-/CA	1	170.11785	170.11785	-	CA	INTRONIC
Parva	1_170305167_-/AC	1	170.30517	170.30517	-	AC	INTRONIC
Parva	1_170357189_-/TG	1	170.35719	170.35719	-	TG	INTRONIC
Far1	1_171389151_-/T	1	171389151	171389152	-	T	UPSTREAM
Spon1	1_171777715_-/ACAGACAGACAGAC	1	171.77772	171.77772	-	ACAGACAGACAGAC	INTRONIC
Spon1	1_171818052_GT/-	1	171.81805	171.81805	GT	-	INTRONIC
Spon1	1_171857492_-/A	1	171857492	171857493	-	A	INTRONIC
Spon1	1_171934624_-/CA	1	171.93462	171.93463	-	CA	INTRONIC
Spon1	1_171992012_A/-	1	171992012	171992012	A	-	INTRONIC
Spon1	1_172005216_CA/-	1	172005216	172005217	CA	-	INTRONIC
Rras2	1_172061298_AC/-	1	172.0613	172.0613	AC	-	INTRONIC
Rras2	1_172087391_-/CACA	1	172.08739	172.08739	-	CACA	INTRONIC
Pde3b	1_172488706_-/CT	1	172.48871	172.48871	-	CT	INTRONIC
Calcb	1_172783191_-/T	1	172783191	172783192	-	T	DOWNSTREAM
Insc	1_172823765_TATA/-	1	172.82377	172.82377	TATA	-	INTRONIC
Insc	1_172830913_-/TG	1	172.83091	172.83091	-	TG	INTRONIC

Supplementary table 4. List of genes in the QTL region that present INDEL

GeneSymbol	Description	GeneID	Chrom	Location (Mbp)
Akap13	A kinase (PRKA) anchor protein 13	293024	1	131.224569
Alg8	probable dolichyl pyrophosphate Glc1Man9GlcNAc2 alpha-1,3-glucosyltransferase	293129	1	154.607442
Ampd3	AMP deaminase 3	25095	1	168.533491
Ankrd42	ankyrin repeat domain 42	293117	1	149.423846
Apbb1	Amyloid beta A4 precursor protein-binding family B member 1	29722	1	163.282918
Aqp11	Aquaporin-11	286758	1	154.973798
Arfip2	Arfaptin-2	293344	1	163.364861
Arl6ip1	ADP-ribosylation factor-like protein 6-interacting protein 1	293551	1	176.332758
Arnt2	Aryl hydrocarbon receptor nuclear translocator 2	25243	1	140.535823
Arntl	Aryl hydrocarbon receptor nuclear translocator-like protein 1	29657	1	171.062181
Atg16l2	ATG16 autophagy related 16-like 2	308865	1	158.787779
Btbd1	BTB/POZ domain-containing protein 1	293060	1	138.006567
Btbd10	BTB/POZ domain-containing protein 10	308890	1	171.163522
Ccdc90b	Coiled-coil domain-containing protein 90B, mitochondrial	308820	1	149.410497
Cckbr	Gastrin/cholecystokinin type B receptor	25706	1	163.156914
Chchd8	coiled-coil-helix-coiled-coil-helix domain-containing protein 8	499214	1	157.965119
Chd2	chromodomain-helicase-DNA-binding protein 2	308738	1	128.609727
Cib1	Calcium and integrin-binding protein 1	81823	1	136.031865
Clns1a	Methylosome subunit pICln	65160	1	154.946673
Clpb	Caseinolytic peptidase B protein homolog	65041	1	159.126512
Copb1	Coatomer subunit beta	114023	1	172.199677
Coq7	Ubiquinone biosynthesis protein COQ7 homolog	25249	1	176.741638
Cp110	centrosomal protein of 110 kDa	361634	1	176.937287
Cpeb1	Cytoplasmic polyadenylation element-binding protein 1	293056	1	137.556774
Crebzf	CREB/ATF bZIP transcription factor	293112	1	147.124224
Ctr9	Ctr9, Paf1/RNA polymerase II complex component, homolog	293184	1	168.784377
Dchs1	dachsous 1	308912	1	163.49734
Dennd5a	DENN/MADD domain containing 5A	308942	1	167.519649
Dgat2	Diacylglycerol O-acyltransferase 2	252900	1	156.447588
Dkk3	dickkopf-related protein 3	171548	1	169.924618
Dlg2	Disks large homolog 2	64053	1	148.051658
Eed	polycomb protein EED	293104	1	146.563964
Eftud1	elongation factor Tu GTP binding domain containing 1	308789	1	138.92062
Fam103a1	family with sequence similarity 103, member A1 (Fam103a1), mRNA	293058	1	137.980239
Fam108c1	abhydrolase domain-containing protein FAM108C1	361601	1	140.382431
Fam160a2	FTS and Hook-interacting protein	293343	1	163.110928
Fam168a	family with sequence similarity 168, member A (Fam168a), mRNA	361614	1	158.140473
Far1	Fatty acyl-CoA reductase 1	293173	1	171.392301
Fchsd2	FCH and double SH3 domains protein 2	308864	1	158.54883
Folh1	Glutamate carboxypeptidase 2	85309	1	142.936379
Fsd2	fibronectin type III and SPRY domain-containing protein 2	308779	1	137.749268
Furin	Furin	54281	1	136.209969
Gprc5b	G-protein coupled receptor family C group 5 member B	293546	1	177.205905
Grm5	Metabotropic glutamate receptor 5	24418	1	143.863506
Hbb	Hemoglobin subunit beta-1	24440	1	161.584858
Hddc3	HD domain-containing protein 3	308758	1	136.161425
Hdgfrp3	Hepatoma-derived growth factor-related protein 3	252941	1	138.098819
Homer2	Homer protein homolog 2	29547	1	137.827128
Idh2	Isocitrate dehydrogenase	361596	1	135.876058
Il16	pro-interleukin-16	116996	1	139.908061
Il18bp	interleukin-18-binding protein	84388	1	159.471809
Ilk	Integrin-linked protein kinase	170922	1	163.481299
Inppl1	Phosphatidylinositol-3,4,5-trisphosphate 5-phosphatase 2	65038	1	159.27813

Ipo7	importin-7	308939	1	167.658312
Kif7	Uncharacterized protein	293047	1	135.473063
Klhl35	Uncharacterized protein	308850	1	156.801077
Lipt2	lipoyl(octanoyl) transferase 2 (putative)	365314	1	157.538704
Lmo1	LIM domain only protein 3	245979	1	166.706026
LOC361635	UPF0505 protein C16orf62 homolog	361635	1	176.965214
LOC683402	similar to AP-3 complex subunit sigma-2 (Adapter-related protein complex 3 sigma-2 subunit) (Sigma-adaptin 3b) (AP-3 complex sigma-3B subunit) (Sigma-3B-adaptin) (LOC683	683402	1	135.653108
LOC689064	Hemoglobin subunit beta-2	689064	1	161.578128
Map6	Microtubule-associated protein 6	29457	1	156.592504
Mesdc1	mesoderm development candidate 1	308795	1	140.157974
Mesdc2	LDLR chaperone MESD	308796	1	140.167851
Mesp1	mesoderm posterior protein 1	308766	1	135.571777
Mfge8	Lactadherin	25277	1	134.87003
Mogat2	2-acylglycerol O-acyltransferase 2	681211	1	156.516533
Mrpl46	39S ribosomal protein L46, mitochondrial	293054	1	134.498792
Mrpl48	39S ribosomal protein L48, mitochondrial	293149	1	157.979818
Mrps11	28S ribosomal protein S11, mitochondrial	499185	1	134.507836
Mrvi1	protein MRVI1 isoform b	308899	1	168.627363
Ndufc2	NADH dehydrogenase	293130	1	154.635889
Nmb	neuromedin-B	499194	1	137.097463
Nrip3	nuclear receptor-interacting protein 3	361625	1	167.380328
Ntrk3	NT-3 growth factor receptor	29613	1	133.92553
Nucb2	Nucleobindin-2	59295	1	174.605677
Nup98	Nuclear pore complex protein Nup98-Nup96Nuclear pore complex protein Nup98Nuclear pore complex protein Nup96	81738	1	159.598048
Olfml1	Olfactomedin-like protein 1	361621	1	164.953064
P2ry6	P2Y purinoceptor 6	117264	1	158.381128
Pak1	Serine/threonine-protein kinase PAK 1	29431	1	155.057622
Pcf11	Uncharacterized protein	361605	1	149.475611
Pde2a	cGMP-dependent 3',5'-cyclic phosphodiesterase	81743	1	158.921607
Pde3b	cGMP-inhibited 3',5'-cyclic phosphodiesterase B	29516	1	172.409282
Pex11a	Peroxisomal membrane protein 11A	85249	1	135.513735
Pgap2	Post-GPI attachment to proteins factor 2	116675	1	159.69692
Pgm2l1	glucose 1,6-bisphosphate synthase	685076	1	157.605906
Picalm	Phosphatidylinositol-binding clathrin assembly protein	89816	1	146.754045
Pik3c2a	phosphatidylinositol-4-phosphate 3-kinase C2 domain-containing subunit alpha	361632	1	174.430831
Plekhb1	Pleckstrin homology domain-containing family B member 1	64471	1	158.08193
Pold3	DNA polymerase delta subunit 3	293144	1	157.453059
Polg	DNA polymerase subunit gamma-1	85472	1	135.197016
Ppfibp2	protein tyrosine phosphatase, receptor-type, F interacting protein, binding protein 2	308918	1	164.979709
Ppme1	protein phosphatase methylesterase 1	361613	1	157.725633
Prc1	protein regulator of cytokinesis 1	308761	1	136.112674
Prkcdbp	Protein kinase C delta-binding protein	85332	1	163.212922
Prkrir	52 kDa repressor of the inhibitor of the protein kinase	308845	1	155.982782
Prss23	Serine protease 23	308807	1	146.081557
Psma1	Proteasome subunit alpha type-1	29668	1	172.237763
Rab30	ras-related protein Rab-30	308821	1	149.656373
Rab38	ras-related protein Rab-38	252916	1	144.783909
Rab6a	Ras-related protein Rab-6A	84379	1	158.041088
Relt	tumor necrosis factor receptor superfamily member 19L	361615	1	158.293361
RGD1306959	similar to C11orf17 protein (RGD1306959), mRNA	361624	1	167.324919
RGD1311021	RCG24599Uncharacterized protein	308765	1	135.69698
RGD1311634	similar to RIKEN cDNA 3200002M19 (RGD1311634), mRNA	293155	1	159.35911
RGD1311703	small acidic protein	293160	1	174.173969
RGD1561459	similar to RIKEN cDNA 1810020D17 (RGD1561459), mRNA	361606	1	154.786525

Rgma	repulsive guidance molecule A	308739	1	128.547399
Rlbp1	retinaldehyde-binding protein 1	293049	1	135.122063
Rnf121	RING finger protein 121	308871	1	159.475046
rnf141	RING finger protein 141	308900	1	168.572375
Rras2	ras-related protein R-Ras2	365355	1	172.026263
Rrp8	Ribosomal RNA-processing protein 8	308911	1	163.47703
Sec11a	Signal peptidase complex catalytic subunit SEC11A	65166	1	137.109468
Sema4b	semaphorin 4B	293042	1	136.001235
Serpinh1	Serpin H1	29345	1	156.667042
Sh3gl3	Endophilin-A3	81921	1	138.398334
Slco2b1	Solute carrier organic anion transporter family member 2B1	140860	1	156.992493
Smpd1	sphingomyelin phosphodiesterase	308909	1	163.27897
Spcs2	signal peptidase complex subunit 2 homolog	293142	1	157.199447
Spon1	Spondin-1	64456	1	171.70072
St5	suppression of tumorigenicity 5 protein	308944	1	167.119711
Stard10	PCTP-like protein	293150	1	158.81997
Stard5	stAR-related lipid transfer protein 5	502348	1	139.896272
Stim1	Stromal interaction molecule 1	361618	1	159.761567
Sv2b	Synaptic vesicle glycoprotein 2B	117556	1	130.683153
Swap70	switch-associated protein 70	293410	1	167.888783
Taf10	TAF10 RNA polymerase II, TATA box binding protein (TBP)-associated factor	293345	1	163.487518
Thrsp	Thyroid hormone-inducible hepatic protein	25357	1	154.647341
Tm6sf1	transmembrane 6 superfamily member 1	361600	1	138.058059
Tmem126a	Transmembrane protein 126A	293113	1	147.129335
Tmem126b	transmembrane protein 126B	293114	1	147.146187
Tmem41b	Transmembrane protein 41B	361626	1	167.608205
Tmem9b	transmembrane protein 9B	293415	1	167.355899
Tpp1	Tripeptidyl-peptidase 1	83534	1	163.490393
Trim3	Tripartite motif-containing protein 3	83616	1	163.331603
Trim5	tripartite motif-containing protein 5	308906	1	162.096454
Trim6	tripartite motif-containing 6	293294	1	162.066157
Ucp2	Mitochondrial uncoupling protein 2	54315	1	157.922179
Ucp3	Mitochondrial uncoupling protein 3	25708	1	157.896001
Unc45a	Protein unc-45 homolog A	308759	1	136.143886
Usp47	ubiquitin specific peptidase 47	308896	1	169.836253
Vps33b	Vacuolar protein sorting-associated protein 33B	64060	1	136.085931
Wdr73	WD repeat-containing protein 73	308751	1	137.088348
Wee1	Wee1-like protein kinase	308937	1	167.769797
Zfand6	AN1-type zinc finger protein 6	293067	1	140.885729
Zfp143	Zinc finger protein 143	361627	1	167.704313
Znf592	zinc finger protein 592	293038	1	137.188336

Supplementary Table 5: Genes within the linkage region represented on the array

Locus_Name	Sequence	Var	Chr	Coord	Final Score
1000316809	ATAATTTTTATTTTTATAGATGTCTAAATTTTAAAATAGGATACACTCAAGCAATATACA[T/G]GTGGTTAAGACAAATTAATACTTTTATAAATGACCATTCTCAGTTACAAGCACCAAAAT	T/G	1	316809	0.493
1002439384	TGGCGAGTCTGGGGATGATAGACAGTAGAGTCCATGATTGGAGATGGGGTTTTAAATATC[T/C]GTTTTATCTGTAAGTTTTTCTGCTGTTTTCTCAGAGCCGTCTGGGTATAAGATGATAGG	T/C	1	2439384	0.903
1006529087	GGCAAAAGGCTAAGCTCCATCTTTGTGCATCAGAGACAGACAGGTACCTTCTGTCAGTCA[A/G]AAGCCAGCTTTGGATGTCAAGAATGTGGCTGGGTTGCTTTTAGGAGTTTTCATCTCACA	A/G	1	6529087	0.981
1010631560	ACTAGGCGACATAGTCTCACAGAAAACTTCCTGATCCTCTGTCTCTTATAATCATTGTGCA[A/G]TTCCATTTTAACATAGTGTGCCTTCAATCATAATTCTGCTAAGAATTTTAGTATTGGGAAC	A/G	1	10631560	0.771
1015047131	TTCTTTAATGGTCTAATGCGGGGTTCCCAACCTGTGGGTCTCGAACCCTTTGGAGGTCAAA[T/C]GATACTTTCAGAAGGGTTGCATAGCAGACATCCTGCACATCAGATGTTTACATTACAATCC	T/C	1	15047131	0.625
1017126515	GAACTTGCAAACTTAATAGTTAGCCCAGGCTAACATCAACCTCAAAATTCTCCTGCGTCA[A/G]ATTGGTAAGTTCTAGGGCTACAGGGAAGCAATCCCATACTGGCCTAGGTTTGAATTTCA	A/G	1	17126515	0.984
1019324003	CAGTCAATACTCTTAACCCCCCCCCGCCCCCGAGCCATCTGTACAGCACCACTAATTGAGG[A/G]TTTTAACTTTTAAAAAATCTAATGGTTGTGGAGGAAAAGTTCTTTGACTAAGACTACTTCA	A/G	1	19324003	0.835
1023571501	ATTAGGGCTGAAGATATAACTCAGGCTAAAGAAGAACTAGCTTTAGCCACAGCAACTCACA[A/G]AATAGGTGTGCCTGTCCTTGCTAGCAATCCCAGCATGTGGAAGTAGAGCCACTGGTTCCTC	A/G	1	23571501	0.881
1028061199	AAGGCACATCCAAAGCCTGAAAAGGCAAGCTGGTAAAGGAATGGACTTTTGGTGGGTCAGA[T/C]AGACTTTGGTGCATATCTCTCATTTACTGGTTAGTTAAGAGCAGGCTCAGTGCTCTCCTTG	T/C	1	28061199	0.984
1043141868	ACAGAGAGAGACAGATACAGAGACCGAGACCACAAACATAGGCAAAGAGATGCCCCCAGGG[A/T]TCTGTTCCTCTCACCTGAACCTTGTCAACTAAAGGTTCCATTATTCCCAAAACAGTGCTGC	A/T	1	43141868	0.865
1045244987	CAGCATTTTGACTTTGCATGATGTACAAAGCAGGCAGGAAATATCAGTTTCCGTAGATGTG[A/T]ATAGCCCTACTTCTGTGTAAAAGAGGCATCACGCATTGAAAGAATTTGTGTCTTTGTTTCT	A/T	1	45244987	0.898
1047282515	TTCTGCTGCTGGTCCTATCTTCCTGGTTAGAGCAGGAAAAGTGAGGTCAAAGATTGGATCC[T/C]GAGGTACCATCCCACCAGGTCCCTTCAGCCTTTGTGGGCACTTCTTTCTCCTGGCTGTCAC	T/C	1	47282515	0.959
1049404004	TCTAGACTAGCCTTTCACTCAGCAAAGATAGATTACTGAGTGTTGAGAGGTGAGGGGTTTT[A/C]AAAGATATCTTGTTGTTGAGGTTGTGACATTAAAAGCTTAGTTCTATGCTGGAAATAGACT	A/C	1	49404004	0.982
1053099529	GCTCAGCCTGCTTTCTTATACAACCCAAGGATATCTGTGGAGAGTAGTACCACACACAGT[A/G]AACTGGACCCTCCCTAAAAATCATGAATCAAGGACATGCCCTACAGATGGCCTACAAGG	A/G	1	53099529	0.965
1055185526	GATTTATTCCTAAGTACCCAGATATATTTATTGTTTTGACATGAGAAATCTAAGTATGTT[A/G]TATAATGACTTGTTACTATGGAGGGTTATGTTTGTTATTTTGATATGGAATGTCCAACC	A/G	1	55185526	0.711
1065906423	CAATCGTGATGAGTTTATAAACCCTACTGTGCCTGGACCTCCACACAGCTGCCCTTGGTAT[T/C]GCTGCTTTCTGTAGAACGAGCAAAGATGGAGGTTCTCTTCCTACAAGCTTTTAACAATTAT	T/C	1	65906423	0.727
1069420737	GGTTGCTGTATGTGTAAGGCTTGTTTGCATAATAATGTACATATTTTACTTTGTATTCCTC[T/C]TTCAGAGAAAGTTCTCCCTGCCAAGGTTATTGGGGAATGTTCTCCTTGCTGAAGTTAATGA	T/C	1	69420737	0.579
1071824081	TAGGTCTTCCTTTGCAAAGAAGTTAACATGATACATTCTGATTTTCATAGCTTCTCTCTAG[A/G]GCCTTCTCCTTCCATTCTTTAATTTCCTCTTTATTTGTTAGAATTTGTTACTGTTTTCTCA	A/G	1	71824081	0.814
1081561140	AAGTCTTAGTAATGTTTCTTTTACAAATGTGTGTGCCAATTTGGAGCATAGTTGTTCAGAA[T/C]TGAGATCTCTTTTTTTCCTTTGATGTGTATGAAGTATCCTTCCTCATGTCTTTTGGTTACT	T/C	1	81561140	0.943
1085654787	GAGGTTGATGGTGTCCAAGGGCCGCAGAGAAGCAGGAGCTTTGGGCAGAGCTGCGTGCATC[A/T]GTGGTCCCCCTTCCTCCTCAGAGAATGGTGTAGAAATACATGGCTATCATCATCACAGGGA	A/T	1	85654787	0.637
1091980360	TAGAACCTGTACCCTGAAATTGCATCCCCCAACAGCCAGGTCCATGTAAGGCAGAAAGGC[A/G]TTTCTTGTGTAAACCTTTTATGCAGGAAGTTGGGATGCCAGGCAAAGGTGTAGCTTCTG	A/G	1	91980360	0.99
1096098605	TTCTAAACTGACAACTCCAAGTTGGAGTCGGGCAAAACAATGTCCAGAGTTAAGAGCCTTT[A/T]TTTCTCCACAGGCACAAACGCCCCATCTTGGCGGACCCAGAAGAATAGGTCTCGGGCAGCA	A/T	1	96098605	0.976
1098244859	CTAATTTCTTTCTCAGCCTGTTAATCATTTGTATAAAGGAAGGCTACTGATTTAGAGTTA[A/C]ATTTATATATGGCTGCTTTGCTGAAGTTGTTTATCAGCTGCAGAAATCCTCTGGTAAAA	A/C	1	98244859	0.49
1100506123	ATATCAGGCTTGACATGACTTATCTGTCCCCTAATTTTATATGAAGCTCTCCTACAATTTA[A/G]GAGGAAAATCAGGCAATGCCCTAGAATCTAGTCAAGGTCAGGAAAGCAATTTATATCCGTG	A/G	1	100506123	0.964
1102564198	CCACTGGCAGTTTATTACATTTACCTTCCCATATTACTGTCATCATTTTGCTTTGGTTTA[A/C]TTTTGCCACAGAATTGTATATATGTTAATGAGATTCTCTATCTGCTTAATTACAACTTT	A/C	1	102564198	0.8
1104596221	TGAGTGACTCTTTATGGAAAATGACAATGTATTAGTTTTCTTTTCAGTAGTTAAAACTAAC[A/G]CAGCAGATTTGTGCAATTTATCCTAAATTGCACCCTGAGAATCAGGTGTTCATAGCCGCTG	A/G	1	104596221	0.772
1106667964	TTCTGGGGAATCAGAAATACTGAGACTAAAACAACAGCATCACATCTCTTTATTTCTTTAA[T/C]TGTTACTTACATAGAAACGCATATAATGGAATAATCGGGCCTAATTTTAATCAAGGAATTT	T/C	1	106667964	0.71
1110776278	TATTTTTATCTTTTTTTTATCTGATAGCTATGACTGGATTTGTGCTTTACTATTTAGAGGT[A/G]GTGAGAGTAGATATTTCTCCTTCTCTTCCCCTTCCTCTTTTTAAAATTTATCTTATGTGTA	A/G	1	110776278	0.75
1112858894	ACTAATGACACAAGGCAGGCAGATGTGAGCTTTAAGCTGCCCTATTGCTAGTATGAAAGCA[C/G]GGAGTCTTCATAAACCCCTGTATAAATTGATCAGGGATAAGTTCAAGGTGCTTTCTGGGCA	C/G	1	112858894	0.505
1114894731	CTGCTTAGAGTTACAAAATATTTTAAGGGAGTGCAATATACTACTAGCAATTAGTCAAGAT[A/T]GGTTTATTTATGTATACCCTTGATCTGATCTCATGGAATACAGTGTAAGGCTAATTTCTGT	A/T	1	114894731	0.585
1119329729	CTACTCTGCAGAACAGACTTTACCAGCACATTCTCCCATCTACAGAAAGGGTCTTTCCCCA[T/C]GAGAAAAGAGCTAAGAAGCATTTGTGATGACCATAGCCAGGTACATGACCTGCAAAAGTCT	T/C	1	119329729	0.98
1124142467	CAGTGGTTAAAAGCACTGGATACTCTTGAAGAAGAGACAGGCTTGGTTCCCATTACCCACA[T/C]GATTGTATAAACCATCTTTAACTCCAGTTTTCTACATTTACCAAGCACCAACACGATACAC	T/C	1	124142467	0.867
1126194113	TCACCGAACTCTACATGAATGACATGAGGAAAAGTTGCATGGGCCCCAACACATAGGAGG[A/G]AGCAAGAGAGGTCCCACATGCCCAGCTATGATTCCTGAAGTAGGAATGAAATGCACTAA	A/G	1	126194113	0.75
1128220967	AGCCTGGAATTACGAGCCAACTTAACCCCTATTCTTCTAAGTTGATTTTGGTTAGGGTATT[A/T]AAGCATAGCAATAGAAATGAAACTTGTAAATGTCCACTAAGGCCTGGTCTTAGGAGACTCT	A/T	1	128220967	0.604
1132288249	GTGTGATGGTTTCAGCATTGTGTTCCCCTCCCCTGCATTTCAGAATTTCTTGGTTGCTTTG[C/G]GTCCCTTTGTGTGCACTTCCACATTGCTTTTCCTCTTTGATAGTATTTTTCAGAATCGCTT	C/G	1	132288249	0.948
1134312415	AGAAAGCAAACCAGTACCAGGAGCAAAAGAAGCAGTGAGGAAAGGCCATTAGGGTTGGGAA[A/G]CTTCATTTCCCTGTGCCAGGCTGAATTAGAAGCCGTGATTTTAATCCATTAACCAATACAA	A/G	1	134312415	0.725
1136332511	CTAGAAGGACAAGGTAGGAAGACTGCAAGCTTAAAGCGTATGTGGACAATTTGGTAAAAT[C/G]CTGTCACAAAGTAAACAGTAAATAAAAACCTGAAACTATAATTCAATGATAGTGTTTGT	C/G	1	136332511	0.856
1138338108	TGCGCATACTACTGCTATAGTTTGAATGTAGAAGGGTCTCTGCTGGCTCATGTGACTGAGC[A/G]CTTGGTCCTTAGCTGGTGGCCCTTCTTTGGCAGGAGGTGGAACATTGAAAAGCAGGAGTCA	A/G	1	138338108	0.719
1146593376	CTATGCAGTAAGAAAAAAAGAAAGAAGAAAATCCTATTTTTGTCGAAGGGGACTTTGAGAT[A/G]GAAGGTGTTCTTTTCAACTGTATCAAAGCCAGCACAATGTTTTAAGATGCTAAGACAAAAA	A/G	1	146593376	0.97
1148739042	CTGAGACCCAAGAAGCTAGTTGAGTTGAAAATTCTCCATTGCCTTGAAGAACAGTACTAAA[A/G]TATCTTCAGCTTTTCTAATGCTTTAGCCTGTGTTTTACTATATCTGCTTCTGAAAAACTGT	A/G	1	148739042	0.903
1150822909	TAATGGAGAATCTATTCCTGACATTTTTCTTAACCAATATAATTTACAACTGTACAGGTC[A/G]ACCCTCATCATAAAAACTTCTTTCAACCCTGAAGATTGTCACAGATATTGTAATCTAAT	A/G	1	150822909	0.809
1152856366	TTTTTATGGACTGGATTGGGCAGATGGCTGTTGACATTTAATGACTTTTAATGAAGCCTCC[A/G]AAGATGCAACCCCTCGATCCTCCAGAAAGTGAAATAAGTCGTTACCAATAAATAAAAATAT	A/G	1	152856366	0.989
1154943209	TGCCAAGCCATCTTTTTAAACCACAGCCCTAACAATTTAAGAGTCTTAGTTGAGCATGGG[A/G]CTAGAACATAGCTGTTAACTTTTGTCTTAGATTAAGAGACCTGAGGATTTGATGCACTA	A/G	1	154943209	0.825
1156997876	CTGCTTCTTTACTTGCAGGGGTAGCTGGGGTGGATGGACACCAAGGCTGGACACTGCTTT[T/C]TAGATTCCTCCTCAAGCTTCCCTCATAGGCATCAATGTCCATGGTGAGAAGTGCTCACA	T/C	1	156997876	0.881
1161067665	ACTCAAGTGAATAAAATACATTCTTTTTTGGTCATTATGAACAATCTGGTCTTTATTATCT[T/C]TCTGGACAGTGAAAGTTCCCTTATGACATCTACTTCATTCATTTCTTCTATTTTTATCTCA	T/C	1	161067665	0.809
1163084697	TTAATGGGGCAAAACTCGCAATTTGGAAACTGTTTACCTCTTAACGTACTGATGCAAATTC[A/G]GATTTAGTCCTCACTCCTGCCCACAGGCACTTAGACAGTGTGCCCTCCATCTTCACAATCT	A/G	1	163084697	0.991
1167133721	TTGCTCTTTGGGGAGAAAAAAAGTTTCTATTTTCAAAATTCTATCTTTCCACTAAGTTCAA[A/C]ATATTTCTCCAAGAACCCCAGAGGAGGTGTGCATCTCAGTCCCTGCTCGAATGGCAGCTCT	A/C	1	167133721	0.935

1169175540	TCATATTCTCTTATAGCCTGGTTATCTTGTTTGGTTATTGCAAGGATACTTTACTGTCAGG[C/G]TATAATAATTACCATTAGGATATGTCCCAGTTTTGATCATTTGGGGGTCAGTTTTCCCTAC	C/G	1	169175540	0.751
1171186665	ATTGTTTGGATATTATGTTACCAAGGACTAAACTCAGAGCCAGCCCCACATATACGTGAA[T/C]ATTTCACCTCTGAGCTATATGCTCAGCCCTACTGCAATATTTAGCAGAGGAAGCAGACA	T/C	1	171186665	0.824
1173205180	CCCATTGCTCATTAGAGGACATATTTGTATTAGATGAGGCTGGGTGGGGGCCTTGCTTGT[A/C]CTCCCACTACCTAAACCAGTAAGGAAGCTCAGGAGTCAAAGGAAGTAGCTGAGGCTGCC	A/C	1	173205180	0.919
1175234094	AGGTTTATGTTAAGCAAGTTACAGTCCATTCTAGAAATCAAGGCAGCAGGAACTTAAAGT[T/C]GCTAGTCACATTGCAACCATAGTCAGAAGTAAAGAGGGATAAACACATTCATGCTTTGA	T/C	1	175234094	0.791
1177281844	GCATATGCAAACCATCACAGCCCCTAAGAGTAGACTTAATGCCTTTATAGGAGGAGGCTTA[T/G]AACTTTCTCTGCTTCCCTGCTATGGAAGGATTCAGTAGGATGGTAGACATCTGTAAACAAG	T/G	1	177281844	0.88
1179326548	CCCCAGAGAACATGTGGGACCGTGACCTCTAGATGAGTGTTTTCTTCCTTGCCTGAAAGTT[T/C]CAGGTACAGTCTCCCTTGTCTTTGTCCTCATCCTCCTTGCAGTCACTGCCAGTCTTGAGAA	T/C	1	179326548	0.85
1181376481	CCCCTTCGCCAACACCTTCTCAGTGCCTTTCAGGGTCGGTGATAGACCTCCATGCATATGA[A/G]TCTAGACTCATGAAGCCCACTGAACCAGGACACCATCCTCGGAGGTCAAATTTACATTCAA	A/G	1	181376481	0.93
1183474096	GCAGGTCAGAGGGAGGTCAACTTATCCTGAGGCAGTGGCTAAACATTTGAAAGTACATTG[A/T]GTTCTGAACTAATTGCAAACCTATTGCCTCCCTTCCGTGGCTGCAGCAACATCCTTGTT	A/T	1	183474096	0.872
1185536653	AGGGGGAGGCCGCGCGCTGCAGGACTAAGGCCAGGCGTTCCAGGAGGTGGCCTCGAGTGG[A/G]GCCTGAGAAGACTCTTGAGGCACCAGAAGAAGACTACGGAAGACCGGGAGCGCTCCGAG	A/G	1	185536653	0.869
1187786202	ATCACCTGCCATTGTGCCTCTTAGCAGACTCAGGGGCTGAGTGGCTCCTTCCCCTCCTTT[T/G]CAGCAAGCTGCAGTCAGGGCTGGGAAGTGGTTTGGTGGGAGAGGATCAGGAGCACAGCC	T/G	1	187786202	0.668
1193936251	ACCGGGAAGTTTTATGAAGGGAACACAACTGGGGAGTCACTACGCTGATGAATAAAAGGG[A/G]GGTGAGACCTTCATGCCTGTCTGCTTTAGGCTCACTGCCCAGCTAAGCAATCCCACTTT	A/G	1	193936251	0.976
1196046075	CACCAGCTAGCAAATGAGGAAAAATTACTGTGCGGCACCTTCTGAAGAGTCTCATCATATA[C/G]TTTTGAATTCTGAACTCTGCACACTTTTTACATATTTAGACATAAAATGATCATAATTGAA	C/G	1	196046075	0.914
1198066256	AAAACTGTTTCTAGAATTTTCTTCCACGTAACAAATAGAGAAAGATTCTCTGTGCTTCTAA[A/C]CTGGGAAGGATAGATAAATTTTGAGTTGTAGTTTCCATTTTTAAAGGATTTAAAGAATGCT	A/C	1	198066256	0.841
1202376557	GGCAGGGAGGAAGGTAAGGAGTGAGGAAGGCAGGGAGGAAGTTAGGGAGGAAGTTAGAGAG[A/G]AAGGTAAGGAATGAGAAAGGCAGGAAGGAAGGTAAGGAATGAGGAAGTTAGAGAGGAAGGT	A/G	1	202376557	0.832
1204394931	TTCCAACTTTATTTTTATGAGAGTCTTTGCCACAGATAGATGCTAAATTTTGTTTAAAAAA[A/T]TTTTTCAGGATAACACCTTTTAAAAATGAACTTTTAATAACAAAATATTGAACCAGCCTTG	A/T	1	204394931	0.741
1206440830	AGCCTGAGGCTACCCAGAGCAACAGTCATACCAGAGGGTGAGCAGGTTGGAAGACTGTCTG[T/C]TCTCAGGCCAGTGCCAGCTGGGTTCAGAAGACTCTGAGGGAGGTTGGCCCTCTCTGCTGAA	T/C	1	206440830	0.872
1208614539	ACTGAAGAGTCCTCAAAGACACACTTGGTATTGAATTGCATCTAGTTCTTACCCTAGCTGC[A/G]TGGCCTTGAAAAATCATGGCCCCTCTCTGGGCTGTACTTCAGTTCCCCCTTTACAAAATGC	A/G	1	208614539	0.979
1211246657	TGAAAGGATTTACAAGCAAGAGGCTGGTGAGACTCTGCAATACCAGGCGTCTACCGTAAGA[A/G]GGAGCTGCGGGAGGCTTTTCGATTCAACAGGGACAATTAAACTTTGTTGGTTTGGAACTGC	A/G	1	211246657	0.863
1213607625	GGATAAGGGTAAGGGATTAATGTCAACTCTAGATAGAGAGAAGTGTGACATTTTTATGTA[A/C]CACCTCTGCCTCTTAACTGCTGAGCCACCCCTCTGTCTTTTGCTTTGGTAGATATTTTT	A/C	1	213607625	0.865
1217741264	TTGACACTTTTACTACATCTTTTCTAGTCATAAAGATAGACATTTAAGAAAAGTAGTTTAT[A/T]GGACCGAGGCAATGGTTCAGCCCTTAGGCCTGCTTGCTGCTCTTACAGGGAACCAGAGTTT	A/T	1	217741264	0.703
1219832931	TTTCAAACTGGCCTTGAACTCTGTGGTTTATATAATTGTCCACCTGCTTCAGTGTCCCTAG[T/C]AACTAGGATCACAGGTCTGGACCACCAACTCTTGCTGATACTGTTTAAATTGTGCTTGCAT	T/C	1	219832931	0.861
1234713288	TCTTTAGCCTGTGGCAAAGTAGCCCATGGTAGTTGGTGCTTGTAATTAGGCAGAACCTCAT[T/C]TTCTCAGCCAGGAAGCAAAGGGGAATGAAGAAAGGACTCCTTCAAGACCCTATTCCCAATA	T/C	1	234713288	0.976
1236768740	GGAATTTGGAAAGTTCCCCAACTAGGACCACACGTGACCTCCTCCTGAAAGTAGTTCCGAC[T/C]GCGGCTCATGTATCCTTCCACCTAGCCTTTGAGCCCTCCCAGGCCTGCTCGCCCCGCCCAC	T/C	1	236768740	0.891
1238809544	TTTTCTTGTTTGCTCCTTAAATACTTTTTAAAAAAAGTCTTTGTTTGAATTTTCAGCTTCA[A/G]TACTTTTTGTCCCTGACGATCTTTGGACCAAGGCATTCAACCACCAACTCCAAATGTAGGC	A/G	1	238809544	0.937
1241096569	ACAGACTCAGAAAAAGCATCCCATTTTCTGTCATATATGGGACCTAAGTTTATGCTTGCAC[A/G]TCACAAAACTAGAAAGCGGATGAGAAGAGGGGAAGAAGAGATCTTAAGAAAGATGATGGCA	A/G	1	241096569	0.909
1245376209	ACGTATTTACAAATCAAGCATCCTTTTTCAGTATTGCAAAAGACTTCCCTCCTGCCAATCA[T/C]ACAGCAATGGTCTTAGTCCAGTTTCAGATTTTATTAGCATGGGTTTTTTTTTCTCTTCAAG	T/C	1	245376209	0.704
1247601695	ATTTGCAAGCAAAGAAGTGTGGATAAATGGGTATACTGTGGTGCACAGTGTGACACATTAC[A/G]GGACGAGATGTTCATTTCTCTGTTGGGGTTGAGGCTGCAATGGTGAAAGTGGGTAGGAGGG	A/G	1	247601695	0.862
1249679196	GTCCTCTCTTGTCTTCTTACTGTCCTGAGCCTACTGAGGATTTCTGCTGACCTTTCAAGGC[T/C]CTGACTGGTTTTTCTTCCTCCAGAAAGCCTTCCCTAACTCCCTAAACCCACAGCGTATGAG	T/C	1	249679196	0.891
1254170679	TTATTAGCTTAGCAGTTCTTAACAGCATCCCATGCAGACATAAGTAATCAAATGGGCTGGG[T/C]TCACTAAGTCAGCTTATCAGTCCATCAGACTAAACTCCAAACACTAGGAACATAGATCATC	T/C	1	254170679	0.96
1256243539	CGTTATTCTTGCTGAGCACGGCATCTTCATGAAGAGAAGAGATGTGTGCTTCTGGCCTCT[A/G]AATGCTTGGTATGTGCCAAATCTACGCTGGGTGTAGTACCTGCAGCATCCTCAACCCTT	A/G	1	256243539	0.865
2000011883	AACAAAAGGGTTCAGACTGTTTCTGAGCTCCTCTTCCAAGAGATCATCCCCAATTTTCAT[A/C]CCGTCAGTGAGACAATGGTCCCGAATTTACTTCCCAAATTTCTGAAATTCTGTCTAAGG	A/C	2	11883	0.55
2002466457	TAGGAAGAGGGTCTAATGGACCACACCCATAGTGACACAACTAAACCAACAACGCCACACC[A/T]GTTTCAACACGACCACACTTTCTAATAGTGCCACTTCCTTAGCCAAGCATATTCAAACCAT	A/T	2	2466457	0.525
2005314110	GGTTTAAACTGGTGTTAAACTCCTCCTGTTGAGGTGTCAATGTTAGCTCAGGCAGAAAAT[A/G]TTATCAAAGTTCTGAACTCCATTCTTCCCTCTAGGTAGCCCCACAGTGTGTTCTTATGT	A/G	2	5314110	0.944
2007385642	GTTGCTTCTAAGGCTGCTGTGGAGTCAGTACAGTAAGGCAGGATGCTACTCAAACCCAGA[A/G]GCTAGGAGCCAAGGTTCAGGACACAGAGAAGTGCCCAGAGTAGGTTGGCGCTAGGAGCA	A/G	2	7385642	0.894
2009622034	CAGCTGAGGATGATGGTAAACTCCTACTCCTGTGGCTCTATCACCTGAGTGCTAGGATCA[A/G]ATGCAAATATTACCACCTCGGTTTACATGGTGCTAGGGTTTGTATCCAGGGCTTTGTGA	A/G	2	9622034	0.927
2011645767	TTCCCTAAAGCTTGAGACATAGCTTCCTGAGGTTGGTGCTAGGAACCAAATTTCATCACTG[A/C]TCTTAACTATGGAACCATTTCTCTAGCCCCAAATTTCTTTTATGTCCACCACATTATTTTA	A/C	2	11645767	0.596
2016098166	TGATTTGAGCATGCATTCACCACTCTGTGTTCTTCAATATCCAACTACAGAGTCTCAGTT[T/C]TACTGGGAGGTTAGTCCTGCTGACCATCTTTCTCTGTCCTTCAAGTCTGAAACTAGCAG	T/C	2	16098166	0.944
2018274737	GCATATACCCACATCAAACACACAAGTGATTAATTGAAAAATAAAAATGAATGTCAAAAGC[A/C]ATAAAGTATTGTTGTTTATTCACCTTGAGACATGAAGCTTTGGCCTTTTGATGATTTGTTT	A/C	2	18274737	0.698
2020856498	ACTCTTGCCCACCTAGAAATGAACAGCTCTGAGACTTGTCTAAATGCTTTAAATTTGATTC[T/C]ATAATTTTGTGTCTAGACAGACGTCAGGAAAGTTCTAGAAGATGGTAGCCAACTTCCCTGG	T/C	2	20856498	0.821
2022884169	CTTTGGGGCCCCGCTCTCTGGGGAAGAAGAGGAGGGGGGTGTAGGGTGTCGGAGTAGGGG[A/C]TCTACTGATAGGAACATGAAGAGTAAATTGGTCTACGTGAGTGGGCAGGAGGGACTCCA	A/C	2	22884169	0.94
2027169154	AACAACTGTTGGGAATCTGTTCTCTCCTTCTATCATGAGTACTCTGAGGATCAGACTCTGA[T/C]CATGAGGATTCATGGCCAGCATCTTGACTGGCTGAGCTATATCTTGCTGGACCATAAGCAT	T/C	2	27169154	0.977
2031259633	ACCTGTCATCTGAGTCTGAGGATCCTCCTAACCTTGCCTTGATCGTTTGGCAAAGCATTC[C/G]TGTGGTCTTAACTCTGCTGTTTACTCTGGCCACTGGTTCCACATCCCAAGACATCCCTG	C/G	2	31259633	0.894
2033357629	GGTCTGGGCGCCACTTACTGTCCTATCATTCTTAATAACAGCCGTGCTTTCGAATGGGAC[A/C]ATGTTTCTCCTGCCACAGATCAAAAGGACTGTGTCCCATCATCCTATCTGAGAAGCCAA	A/C	2	33357629	0.806
2035402978	CATACATTCCAAAGGCTTGGCTTACTGTGACCAACACATGACAACTGATGCTTGCCCAAGA[T/G]GGAGGCAGCCTCTGGAGAAGTATCTGGTGCCTAATTCTTCAGGGAAAAGTTTCCTTTGTCA	T/G	2	35402978	0.766
2037535509	GGCTAAGGTATGTGTTGGGATCCCATCCAACTATGCTCAGGTACTTTGCTGGCCTGGTAGT[T/C]TGTGGGCTCAGATATAAATACACAAAGTCTAGGTCGCATTATATTATATCAGTTGACCTTC	T/C	2	37535509	0.989
2039576817	GCATCAGTTTTTGAGTTGGCTGTTCTACTCCTTAGTTTGCTTCTCTCATTTGAGACATTGG[T/C]GCCTGTTGGCGTTTTTGAGTAAGGCCCACTTGAAGACAGCTTTACAAACGAAAGTCCTCAA	T/C	2	39576817	0.973
2041585226	AGCTAGCCAAGGAAGGCCCTATTACTCTGTAGTTTGCACAGTTATCCCATAGGCAGGCTT[C/G]TTTCTCTTTAAATACCAACATGTGCTTTTATAAAGAAATTGATTTCAAGATGTATCTAC	C/G	2	41585226	0.958
2043600678	GAGTCCTTCTCTGCCTTAATTTCTTGAAACTGCTGTAAGATTATCGCAAGCTTGGTGGCT[T/C]CCATAACCAACCAAAACTTACAGTCTAGGAACGAGAAAAATCAAGGTGTTGAGAAAGCC	T/C	2	43600678	0.997

2047930597	AGGAAAGAAGGGAGGGTGGGAAGAAACCAGGAAACCTTTCATTCACTCCAAGACAACAACC[A/G]GAAAACCAGCATAGTTCATTATTCTTGAGGGTAGAATTGTGCACAACATTGTACAATGGAG	A/G	2	47930597	0.956
2050000379	GTGCATTTGATTTCTAACAGATTTTAATACTCTTGACTTTCTATTTTAAGTGCAGAACTC[A/C]AACAGAACCCTAGTGTCCAAAAGATTCACAAACCTTTCTTGTCATATGATACATTCCCC	A/C	2	50000379	0.867
2052066591	CAAAGATATTGGCTTCACCCAGGATCGCAGGTTTCTAGGATTCAAGGGAAGGCCAGGAAGA[T/C]GGCAACCCTGATACAACCTGCAGTGGGAAGGCAGCTCTGGCTTCATGTTTCTAGCAGTTCA	T/C	2	52066591	0.674
2054131845	CCCCATCACCTGCAGCAGGCAAGAGACCTGGTCCTGGGGTTATGAGAACAGGAGGACTAGT[T/C]CTGCCCCTCAGCAGCACTCAAGAGAGCAGGCACCTCACCTAGGCAGCACGGTAGAGCTGAC	T/C	2	54131845	0.961
2056283433	ATATGGCCTTTTGTTAAGTGGTACCCAATCCCCTTTGAAACAAGATACTCTATCAGTACA[A/G]TGTGCATTCCCAGTCAGAGCAAAGGTAGGCCACACACAAGAAGTATGTTTTGAAATGCT	A/G	2	56283433	0.874
2060415706	CCATATCAGTACCTGTGTTTCCATTTCAGGGCTCATTTCTCTTTTCCCTCTGCTCATATT[A/C]TACTTCTTTTCACTGTCCAGGAGAAAGTTGGTTCCTGCCATTCCTATTATATATCCTTA	A/C	2	60415706	0.868
2066704298	AAGCATTAGTAAATTTCTTTTCCAAATGGGGTTGATATCTTACATGTAGTTACTAGCACCA[A/G]AGTGATTTCCTGTAGAACAGGTTCTTCTGCTGGAGTTTTGGGGCTTTTGTGTATATCTATC	A/G	2	66704298	0.72
2070774696	ATGTATTTGAAGAAATAAAAAATGCTTGTAGTAGAATGAAACTGCATCTTTCACATCTTG[T/G]ATTTTAATGGTATTGTTTTGCCTCTCCATTCATAAGAAAATTACCATCTTTAAATTATG	T/G	2	70774696	0.919
2074816527	AATGATAGTGGAGAGAATAAGTGAATGGCTGACCAAAGACTGCCCCAAACTGAGAGCCAT[T/C]CCACAAGAAAAATCAATCTATGACACTATTATGATACTTTGTTATGTTTGGAGGCAGGA	T/C	2	74816527	0.701
2076840315	CTCAAGGGACAAGTGGTTGCCATAACTGAGAAGACTAACAGAGGGATTTTAGTCCTCTAT[A/G]AGCAAGTATTTTAGATCAGTCTGGGGAATAAAATAAGAAATAAAAGCAACAATTCTGGT	A/G	2	76840315	0.906
2078881254	TAAAGTAACTAAGTTAATATGTAAGCACTCTACCAACTGAACTATGTTGCCAGGCCCGGTC[A/T]TCTGGTTTTCATGCTAATGGTCTCATCTGTGAAGTGCTCTACAAACACTCCATAATGCTCA	A/T	2	78881254	0.901
2081114629	AGGACATTCAGCAGAATCAACCAACCTCAAACCTTGGGGCTTTCAGAGTCCAAACCACCAC[T/C]CAAAGATCATACACAAGCTGAACCTAGGCCTTCCTGAACATATATAGCAGATGTGCAACTT	T/C	2	81114629	0.566
2087350375	AGCTAATATGAAAGAGCTGAAAGTGATCCAGCCTGACAAATCAGTTTCTGTTGATGCTGGA[A/G]GATCAGTCACTCTGAATTGCACGGTGACATCTCTCATCCCTGTGGGGCCCATTAGGTGGTT	A/G	2	87350375	0.744
2089384684	CTATGTTCTTCACACATATATAACATGTAGGCAAATGTTTGCTTGCAAACACATGTGCATA[C/G]CACTCCACACATAGACACAGATAGACAATATACATAATCCACATATATTTAAAAATATAAA	C/G	2	89384684	0.993
2091485272	CCCATTCAACAATTCCGTCTACGAGCATCCAAGTGAGTATGGCCTGCCTTGCTGATGGAA[A/G]CACAGTGAGCAGTGAGTCCGCATGGCAATGAGATGGAAGAGGTAAAGAGAAGGCAACTC	A/G	2	91485272	0.585
2095616472	AACAATGGGTTTCAATATGACATTTTCATATACATACATGTTCTACTTTGATCATCCATG[A/T]TCAGCCTCCATTACTCTTTCTCAAGGCTTTAAGTATTGAGTGTCACAATGTGAAGCATA	A/T	2	95616472	0.949
2097793508	GTGTTGACTATCACTTACATTATTATTTGATTCCATAAAAATTCCCGTTTGAAGTCTGGCT[T/G]GAAGATGAATCCATTTTAAGATACGAAAAATATGCTGTCTCTCCGGAAGCATCTACTTCTT	T/G	2	97793508	0.614
2102112098	TAAGTTCTTCATCTTCACTTAAGACAAGACTGCCATCGAGAAATTGAAACATCCATCAAA[T/C]TATTCAACAATTCAGATTGAAAAGGGCCTTTAAAAATTATTCTAAGCTGCACATCCTAA	T/C	2	102112098	0.963
2104290705	AAAAGAAAAAAGAATGAAAGAAGGAAGTAATTCAACCTGGTTTTCTTCTAGCTGTGTCTC[T/C]TTTTTAACAATTTAGGTTTTAAAAAAAAGGAATAACTGAATAACACTATTTGGGACCTT	T/C	2	104290705	0.849
2106347084	TATATGTATATATTATAGAAAGCTACTACTGTATTAGGTTTGCTTTTGATCCCCCAAGTG[A/G]CACTTAGTTTTAGTTGTCCCTCCTAGTATTCCCTCCATGTCCCTTCCTCTCTCCCTGCA	A/G	2	106347084	0.733
2108423278	CCACTTTGGCTAATTTACAAAGTTTTAAGAGGTTGATTTTGACTATATTTTTCTTTTGTT[T/C]GTTATGTGTAACTGCCTTTCTGAAAAGACTTATTTGGGGGGATTCTTATTCCATCATTC	T/C	2	108423278	0.779
2110513518	AAAGAGACCAGAAATTGATGGGTGGTGGGAAATTTAAGAATCAGAATCGTTCTGGAAAAC[A/T]CCTCAGGATTCTTTCTACCTAAGGAGTAGGTAGGAGACCCACCACTGTTTTTGGCCTGA	A/T	2	110513518	0.876
2112598040	GCCAGCTGGTGTTCATGGGCCATTCTGCCACCAGGGAAAATATTTAAATGAGTGGCTTCTG[T/C]TGCCACCTGAGGTCCAGGCTGCCCCTGAGAGCATTGTCTGGGTCCATGGTCTTACAGTAGC	T/C	2	112598040	0.926
2114705253	GCCACGGAGGCATGTGTGCCACTACTGCACCTGAGGAGTTACTGCCTCATCCTCACTGCC[A/G]TGACTCACAGCTGTCTTAGTTGGGTTGATTGTTGCTTGCTTCTCTTAGAAGCTTACATG	A/G	2	114705253	0.799
2116734555	AAAGGACACCGCTTTAATGTCCTGTAACATTTGATACAGAAATAACTAAATTCCTGTGCA[T/C]ATTAATAACCATGTCAGCTTTAATTTTACTTGTATTTATTTCTATTTATTTGAGACAGG	T/C	2	116734555	0.779
2120976129	GAGGGACCATTCGTTTTATGACCCCATCTGCTTGTCTATAGCGAGTCAAGTGGACACGGA[T/C]GTATAAAGATTTCGGCGCTTTGAACCACTTTTGCTTCTCTAGGCCCAGGTTCGGCAGGG	T/C	2	120976129	0.903
2123134658	GATTCCCCAGCGCATACAGACGGCTAAGCATGGATTGCTGGAAAGTCTTGATCCACACATG[T/C]CCTGCACTGGATGCACCCTGGCTGCTAGCCAAACACACGGCAAGCAGGGTGGACTCTGAGG	T/C	2	123134658	0.917
2125287256	AAAAAAAAAAAAAAAAAAAAAAAAAAAACCAGAGAGCCTGAGATCGTTTCACATTTGAACT[A/G]GAGACACAACTAACCTGACCAGCTTGCATCTGGCCACCATAACACAGTGTGGGAAGCATCT	A/G	2	125287256	0.902
2128187827	ATGGGAGGTGCTTTCTTCTCCCCAGCTACTTGTCTTGAGAGTCTCAACTTCCCCCTCCATT[A/T]CTTCATTCAGAGTCAGTGTCAGCCATAGGTGCCAACAAGAACATATGGTCTGTGACATGAG	A/T	2	128187827	0.93
2131121098	TAAGGCAAACTTGTTTGTCTTTGATAAGAAGTCTGGCCACCTTAACTAGTTAACAAATTGA[C/G]CAATGAACTGATACTGCGGTAGATTACATTGCAGTAAGGACCTAGAGGTCTGTTTTTGAGC	C/G	2	131121098	0.861
2133968266	CATCCAGAAGGCTAAATGTCCTCAAAGGAACACAAGCAAAAACTGCCAGGGCTATGTGACA[A/C]CTTCAGAACACAGCCACCCTCCTGTAGCAAGCACACCTAACAGTGTCAAAGCACAAGAAAA	A/C	2	133968266	0.778
2144715765	CCGAGTCATCTCCCCAGCCCCGACATTGCCATTTTTGTGAGCATCTACATCCTATTGTAT[A/G]AACACCCTCATTGTTTGATCAGCACATTTCCACTTGCCATTCCTTCATTGCTTCTAATG	A/G	2	144715765	0.978
2146756377	GATGTCCCTCTTTTTAATACATGATTTAAAAAAAAAAAAACCATAAGCCACAACTTACAC[T/C]GTGGCTGTCAATCTGCTGCCCTCTAGAGCTCACTGGACATTCCCGGGTACATTCTCACC	T/C	2	146756377	0.964
2157090038	CATTGTTTATGTCCTCCTAATAGAAATCCTATTTTATAAATGCATTGGCTCATTTTTTAA[T/C]GTTTCTTGACTGCCATTTGCAAGTATCTATTATATATTGGGAGATTTTCAGGGTGCCTA	T/C	2	157090038	0.828
2159122474	GTGTTAGAAGTTCATAAATGATACAAAATTCCAAGAATCTCTGTGCAGAAAGGAATAGTAA[T/G]ACCTCACCTATAGACTATGCTAGAAGTACATTAGGTTGAGAAAAATTAAAGATAGTAGGAT	T/G	2	159122474	0.793
2161342675	CCTTGTAAAGAATGAGAGGATTGAATGGTAGGCCAGTTTTTTGACCACCAATGGGAGAAGA[T/C]AATACTTTTATCTGCTCCTGTATCTAGAATGCCTGTGAATTGCTTTCCATCAATTAGCAAC	T/C	2	161342675	0.765
2163515761	AATGAGACATGGGTTTAGACAGGTTGGATAGAGACATTAATTTCCACAGGCTGTTTGGAAA[A/T]GACAATACAGAAATCCCGTTTTCAACTATAACTCAAGTACTCCTTGTCTATCTTCATATCT	A/T	2	163515761	0.944
2169717338	AGGGCAAAGAATACACAAGCTCATACAGTGCTCCTATTTAGTTTTTCACACCAAGCCCTG[A/C]AAGTCCTTTGTCAAGATCATGTAGAAAAGAAGACCTTTAAAAAATGAGAGGAGAGATAA	A/C	2	169717338	0.976
2171951621	TCCCTGTCTCTTAAGGAACACATTTTACAAAGTCTTGACACCAGCTTTGAAAGCATCTTC[A/G]CCTGGGCCTGAGCCCTGGGAATTCTCACCTGACAGAATTTCCTTACCTCTAATTTGTGA	A/G	2	171951621	0.813
2173966193	TGTGTTGTCTGTAGACAAGTTCTAAAAGAATATGACCTTTAGTTGCCAGCGAAAGCAACGA[A/G]AAAGGAAAAAAGCTACAGCTGTGGTAAACGTTACTCAGGGCCAAGCTTCTGATCTTCAGGG	A/G	2	173966193	0.932
2180060571	GTAGATGGGGATTCCTGGTCCTCAGGGGAAGACTAGAAACTGCCCCTCTGGCTCTGAGGA[T/C]GTACTGGTGGGGATGTCCCTCCCTGCTCTGGGTGACCACTCTTAGCTTTGATAACTTGA	T/C	2	180060571	0.682
2182080587	GACAATGGACACCCATCCTCTCTCTACTTCTGCTCTGCTTCTCTCCTGAAGTCCTGAGAT[T/C]GAAGAAAATAAAACCTCAAGTCAGCAGTGCCTAGGATTACTATTCACATTTAAAAACTG	T/C	2	182080587	0.944
2184309455	CCTTGTTTGGTGATCATCAGAGAAGGAATACAAGAAAGAAGAAACAGGTCAAAGAGGAAGA[A/G]AAGCCATTATGGGATAGATAAGACATTACAATATTGTTATGAGGCCGAGACCAATTAGAAT	A/G	2	184309455	0.652
2186441049	CTTACAGACTGCTTCCTACACAGGATAGAGGTAGTCAAGATGTATTAACCTGAGAGGATC[A/G]GTTAGCAAATGGTAGCAGAAGGAACTTGGGGGGAAATTCAGAAACCAAGAATGGGAGTC	A/G	2	186441049	0.908
2192611280	AGTTCTTACTGGTATACAAATTTTACATGTAGCGTATGTATGCCTCAAAAATCCCACTTC[A/G]TTGCAATCTCAACTTCATTCATTACCAGACTGTAATTCTGGACCCCTGTTGACTGAGGA	A/G	2	192611280	0.95
2196682421	ACAGGTGGTTAGGAGCCTGGGAAGCAAGCGGACTGCTGGGCCGGAAGTGGGCAAAGGTGCT[T/G]ACTGGCTTCAGAAGCGCAGCTTGGTGCCATGGAAGCACGACAGGGAGCATGCAAACAGAGG	T/G	2	196682421	0.638
2198706857	AAAGAACAGTAGCAAGTAAAGTTAAGAAGGTTGGAAAGGATTTCTTAGGAAAACTGATAA[T/C]TGATAACTTCAGCCCACAGGAGGGGATGCTCTGTGAGTCTGATGGTGGCTTTGGTCTTG	T/C	2	198706857	0.984
2205116571	GTCAAATCATGATAACAGGTTAAGAAGCTGGCTGATCACATTTTTATCTAGACACAGGAA[T/G]CAAAGATTGAGAACAGGAAGTAAGGTGAGGGTGAGTAACATTAAAGACTGTCCCCAGTG	T/G	2	205116571	0.709

2209434768	CCATGACTCCAGTAGAATCGCCCTGCTCTACCAGTGGACATGCTGGTATCGAGAGCCACC[A/G]GGCAGGTCCTAGGGCAGACCACAAATTTCCTCAGAGTCCCTTCAACCTGCCCCAACAGT	A/G	2	209434768	0.641
2211589987	GATAACTGTCCCAGGTACTCTCAGAGGCATGTCTCTTAGTTGATCCTGGATCTAATCAAGA[T/C]GACAACCATGATTAACCATCACATTGTGTATGACAGGGAATTTGAAGAAAAAATCTTCTAA	T/C	2	211589987	0.84
2220105538	AAAAGCACATAAATCCTGTAGTCTCTAAAATATTTTATGTTCTTTTCCTAATTAACTTAA[A/G]AATCCACCTGAGAAAGAATTCAGAACGAAACATAAACACCAACTTACTACTCTCCCAAG	A/G	2	220105538	0.774
2222168234	GATTTTAAACATTTTATAGACTTAAAAATAATGTATTCAGTCTACACAACCTGTGTGAGTC[A/G]GATGTTAAATACTTGGTCTACCCAACACAGGATTATCCCCCAGAATTGGGAAGATTTAGTA	A/G	2	222168234	0.898
2226297591	GAAGAAGGAAGAGGGAGGAAAGAATGGGGGATAGAAAGGGAGAGACAGGAGAAGATGGAGG[T/C]GGGAATGGAAATACAAAGCTAAGGGAAGTGACTGTGTCTGTTTAAAACCATGCAAGTATG	T/C	2	226297591	0.981
2228473263	TAAATGGGATGTCTCTTTTAAGCCCCTCCCCCAGAGCTCAAGTGACATGTGGAAGAAGAG[A/G]CGAAAGAGGTAAGAGCGGAGGTCATGGAGACTCTGAAGAAACAGTATCGTCCAGACGTG	A/G	2	228473263	0.952
2230682421	TCGGTCTTGCCCCGACACTGTGCTCAGGACGGATGTACTCAGGCCATGCTCAGATGCTGA[T/C]GGTGCCGAGATTGAGGGCAAAGTTCCTGTGTGTGCAGGGCATAGCGTCTGACTTCACCC	T/C	2	230682421	0.644
2234764832	GGAAAGTGGGGGAAGGAATAAAAAAGAAAAAGTAGGGATATAGGAATAATAGAGAAAAAA[A/G]GTAAATTATTGAATCTACATTTAGGCCAAAAAGTACTAGCCAGGAAAAATATTATATTG	A/G	2	234764832	0.573
2240905173	GAAGACCCGACAGAACATCCTCCGTGGGGCCTGGACTTGGGGAGTGCACACAGTCAGCAC[A/G]GTACCTTGGTCCCGGCTGCCCAGAGCCTGGCTGAGACTGTGAGTGGGCGCTGGCGCTCC	A/G	2	240905173	0.739
2245080775	GGATCCACTTCCGTCCTTCTTTCTTGGGTTATGACACTGGGGCTCCACTGAGGCAGTTGG[T/C]TCTAAAGGGTCTCTGGTAGGGGAAGGGAGACAAGGGGTATGACTACTATGGAGGACACA	T/C	2	245080775	0.894
2249118552	TGTAATACTGGAACTCTTAATGGAAACTTAATGGAAACAAAATAAAATCTAAAAACATTTG[A/G]CACATAGTTAAATATTCCAGAAAGAGACATTCATATGTAGTAAATGAATAAATTTAAGGAA	A/G	2	249118552	0.65
2251155448	GAATTCTCAGAGTAATCAAATTGTTTTCCAGGAAAACATCAGAAGCAGCAAAATATAAAAA[T/C]ACCGAGAGCAAGAATATTTTAAATGCCCCCAAGGAAGCAGCTAGAAAACCCGATGTGTGGT	T/C	2	251155448	0.913
2255556330	GAAGGAAGCCAGGTCAGTAGTCAACAGGGAAGCCTCACCTAGGCTTGCTGCAGTTAAATG[A/G]ACCATCTCTCTCAGTTTAGCAAAGCTCATTGTACTTGTCCTTGCAGAATCTCATGCTCC	A/G	2	255556330	0.955
2257673789	CCCATAGGCTGCAGTTTCTGGTTTCAGAGGAACTCAAACAGGCAGGTCAACTACTGAAGC[A/T]AAGTAATTTAGACTTAAAACAGGTAAACTTTTATTATGAAACAACAACTCTAAATATAT	A/T	2	257673789	0.623
3000090664	CTATTAGTAATAATAAAAGATTCTTCTATGAATAATTTAGACAGTTACATGTTGTCTCTGC[T/G]CATAGCTCCACATGTACTGTGGGGTCAGACTTCGAGAGATTCATTATGTGGGAGTGACGTT	T/G	3	90664	0.595
3004079793	CCTGTGTCCACATCCCTGACAGTGATCCCCACAAGAGACCCCACAGCTATGACCGTGGGGC[A/C]CATCAGAGTTCATAGACCTCACCACAGGACCCACAAGGGGCTACATGTCGATGACTAAGGG	A/C	3	4079793	0.82
3005824425	GGCTAGAAAGAAAGTGCTGGGATGTCCGCACAGCCTGTATGACCTAAAGCCAGGTAAAGAC[A/G]CCAGCAATGACTGCAGTGAAACTCACGTGTCCCAATCACCATCAAAGGGCAGTTCTGAAAG	A/G	3	5824425	0.975
3009068961	TCCGAGCCTGGACACCGACTTGCCCTGTGCTAGAAACTCGTGTTTGAGGCGGAGTGGGGGG[T/G]AGTCGCTTTCCAGTTTCTCGCTCTGAGCCTAAGCGCTCCTTATCTGAAGCGCTGCTGGCCG	T/G	3	9068961	0.674
3016151838	ATCACTTACTCCAATTCTTTGTTTATGCTTGTCTGAATTCTCAAGGGATTTATTAATTTC[T/C]TCATTAAAAATTCCAATCATCTGCAAAAAGCTGGTTTTATGGTCTTTTTCTTGTGTTTC	T/C	3	16151838	0.896
3017874135	ATTATGTCAATAAAGATGGCAAAAGTCAATTGCTAGGCGAAGAAGGTACAGAGGTCCAGG[A/T]CCCAGGAGGAAGAGGTGGAAAAGATATAGAGAAAGGGCTTTTTGGCCAGGTTTTGGAGA	A/T	3	17874135	0.804
3019581047	TAATATAGTAGTGGATTACCTTAGGGTATGAGCTAGGTCTGCTTGCTAGCTGTCAAGCTAC[A/G]GTTACCTTAGCTGTTAATGTAGTTAGAAATCTGGGAAGATAAATTATAGTCTAGGACTCAT	A/G	3	19581047	0.531
3021115954	CATCATTGCATAGACAACTGCAGATAATGCCTTCTCTTCAGGAGATATCAGTAGTCAATA[A/G]TTTGTCAGAGAGTGGTGGTCTCTATTAGCCTCTTATAAGTCCATGACTGACTACTAAAA	A/G	3	21115954	0.871
3022640856	GGGTGGTAGAAGTAGGGAGTGTGAGTAGGGCTGCAGAGCTCAGGTCAATCAAAGTTTGTC[A/C]CAACTATGAAGAAGGTGATGATTTACTTAACAAAATAAAGAAAAGAGAAAAGTTCTCAG	A/C	3	22640856	0.915
3032072437	CATTTTCTCATTTTGCTATTTATAGAGTGAAAATTATTACTGTCCGTGTAACTTCAGCGCA[T/C]TCTGTGCCAGTGCCTGTACTGGGCTCTGCTGATTTATAGCAGTTGTTTCCAGAGGGAAACT	T/C	3	32072437	0.821
3033793044	CTGAAGGGTTATCAGAAGACCAAAAAAAACCAGATCTAAATGTAACACAGGGCTGGCTTCA[A/G]CCTCAGTGAATTTCCAAGGATGGTCTTGAACTCCTGATCCTCTCGTTGCTGCCTCCTGAAG	A/G	3	33793044	0.935
3035446267	TTCAAATACTCACAGGGATGGTACTGGACGGGGAACCTCACAGTAGTTAAGAACTAATACC[A/G]TTGTCTAGCTACGTTGTGACTGAAAACACAGAGAGTGGTAAAAAGGAGACTTATTTACACA	A/G	3	35446267	0.731
3038942593	GAGGATGAAAATCGGGGCACGGAAATGAAGAAGCCAGGTATAATTCTCACGTGCAGACAC[A/T]GAGGATGTGGAGATGGCTCCTGGAAGGCAGTCATCTGCTTGCATTTCCCACTTCTCGGG	A/T	3	38942593	0.986
3040597508	GAGCTCTTAGCCATTAGCTGACATGCCTGCTGCTTGCTGTCATTCTGTCCCACCACAAAG[A/G]ACTCTTAGTCTCTGGGACCAAAGGGCAAAATAAACTATTTCTTAAGTTGTGTTGGTCAT	A/G	3	40597508	0.965
3045763581	AAGAAATTTATTGAGAAATGGCAAATAAATAATTATTCAGAGACCAAATGGAAGGTAGTA[T/C]TGACAACCATTAAAGCCCACATCCAACACAGAAAAACTAAGAGACAGGAGTATTGGTGG	T/C	3	45763581	0.948
3047346760	ACAAATGTCACGTGCACACCCCTCACTTTTCATCAAATTTAACATGAAAACAGTTAAATC[A/G]AAAGCCAAGCGGGGAACACGTGGAGTTCCTCCCTTGTTTATTTACTGTTTTCAAGTTAT	A/G	3	47346760	0.768
3048920359	TGGTTACAGAAGATGTGCAGTTCAGGCTCCATATGATTCATTATTAGGAGTGCTTACCAAG[A/G]TCATCCTCATAGACACCACAAAGTTTCTACTAGACTAGGTTTCCACATTGTCTGTATATGT	A/G	3	48920359	0.911
3050437769	AAAAGAGAAATCAAAAGTTGCTCTTTCCTTATAAAATTACAGAGGCCAAGGTCAGTGTCC[T/C]GCTTGCCCTGCTGTCCACTCTCACCGGAGGGTCCACAGAGAGCACGAGGGGCAAACAGA	T/C	3	50437769	0.799
3051998320	CCCCTGCCCCCCTTTACACTTTTACACATCTGTGGCCAGTTTTATTAAGCTGTAGGTTGGC[T/C]TCAGTGGCTGAGCTCTTGCCCAGTGGGTAGCTCTCTGTACTGTTAGAGCAAGGAGATCTTA	T/C	3	51998320	0.971
3053681912	GAACAGCTGATGGGTAGGAAGAAAATCAATACACAGACAGGGGAAATGCAGCCTTCCTCA[A/G]CAACGTGATAATTCTTTCCCTGAGAGCCCTGTGACTGCACAGTAGTGTGGATTTATGAC	A/G	3	53681912	0.942
3055246649	GGAGGCATTTTCTCAGCTGAGGTTTCTTCTTCCCAGACCACTCTGCCTTGTGTCGAGTTGA[T/C]ATAAAACAATCAGCACAGTGAAGCTGAACATCTTTCGCTTATCTACTGGCTCTCTGAGTAT	T/C	3	55246649	0.708
3056914987	CCTGGCTGCTGTCCTGGAATATGTGACCACAACCCCCACAAGAAGGTCATGACCACTAGTC[A/G]TGTAGCACGGAGTTCTCCATACCAATGTGGTATCTATACGGGCCCAGAGGGTTTAGCCAAT	A/G	3	56914987	0.822
3058625138	ACCAGACACAGGCAAACTAAAAACTGGCATTTTGATTGATCACTTACTGGCTTCCTATGAT[T/C]GCTAATTCTAGTAGTTTCTCCACTGTTTTAGAGAGTGGGATGGATTCTTACGCCATGGCTC	T/C	3	58625138	0.801
3060223303	CTTTTGGAGAACGATCCAATGATGTCTCCTAAAATCAGACAGTAGGCTCCCCTTTTCTCTT[T/C]TAAGCTAACTGTAGTTCCAATTAGGGGGCGTAATGCGGAGTTGATCATTACCAGGTAGATA	T/C	3	60223303	0.949
3061784861	TTTGTGGTTCTAAGTGAATATTCTGTGTGGTGGGAGAATACTGATTTAAAATTACACTTC[T/C]GTTTTTAAGAGTTCACCCCAGGGGTTGATATTAACAAGTACAACATGTGGGGAATTGGT	T/C	3	61784861	0.862
3063309021	TCCGCCCGTGGAATGAAAATACTAACACCTCCTCCTGGGGCTAGCATTAGGACTCAATAGA[T/G]TCATACACACAAGAAGCTTAAAACAGGGCCCACCATACAGCAAGAAAATACATTTATGTGC	T/G	3	63309021	0.943
3064922447	CTGGGAATTAGAGCTCTATAACTCACTCTTACCCAGTATATACTTTATTGATATATTGAA[T/C]GTTTTACAGGCACGGTGACAATCCTTTATGAAAGTGATTTTGTATTAGAGCCAGAAATA	T/C	3	64922447	0.666
3066438824	TTCTTCTGGTTTTTCAAACAGCTGAAACTCTCTGGCCTGGGTGAAAACCTAAAATTCCAG[A/G]CTACCCAAAATTAATTCAACTGGCTCAACCCATTGCCTGGTCAAGAAACTGCATCTCAG	A/G	3	66438824	0.96
3067974744	TTTGATCAAAATAGATCAGAACCCTAGGCTTTGCTTATGCTTCAATGCAGAACCTCTAAC[C/G]TCTAACCTCTAGTCCTGCTGTTAACAGGTACATAGGACTTTCTTATTTTAACATCTTCT	C/G	3	67974744	0.904
3069585117	CTCTGTATTTGAGCAGACCAAAGATAACAAAGGAAGACTGTCACAGTAGAAATGACTGAT[A/G]ACATTGGAGCCACAGAAAGACAAAGTGAAAATCTTAGTTGTGATTAGAAGAGCAACAAG	A/G	3	69585117	0.752
3071302240	GGACATTGCGATTGTATAGAGCAAGGGATTGCAGATAGCAACATAGCGACCATAGGCCAT[T/C]ACAGCTAGCATGAGGCATTCAACATCTCCAAAGGCTGCAAAAAAATACATTTGAACAGC	T/C	3	71302240	0.55
3073091270	ATCATATTATTAATTGGATGTACCCAAATTATGCAAAGGTGCTTCACCAATGTTTCTAAG[A/T]CTTTATGTTTAATTTCATACATCTTCAAAAGCATCTTCATCATCTCTACCTCTTCAGAC	A/T	3	73091270	0.847
3074637142	AGTTCTATGGAAAATCATAATTTATTTCCTACATTATTTTCATCTTAAAAATAAAAATCAG[T/C]GCCCATTTAAAAAAAGAAAATTAATCGCACGTGAGTAAGGGAAGAAGAGACGACTGTTGGA	T/C	3	74637142	0.666
3079526168	GTTTGTTTTTGTAGGTACATAGCCTAAGCTATTTGACACTATTGGCACAGGTGCAAGAAGC[C/G]ACAGTAAAAATGGAGAAAAGTATGTTGACTTCTGAGTTTCTATAAAAAGAAAATGCTTCAA	C/G	3	79526168	0.97

3081263952	GTTTTTGAATCACCTGAAATTAACAAAAAATTTCTCCTCTAGTATCTCATTAATTCAAGT[A/C]TTCTTATAAGATGTTGGGAAGAATGGGCATGATAACTTCAGTAAAAATCCAGCAAAGCC	A/C	3	81263952	0.83
3082915876	ATATGATGGTAGATAAAATATTCTGTAAATATGTATGCATATAATGGTTCTGGTAGAACTG[A/G]CGTGAGTCAGTTTGGAATTGAATCAGTTCATCCATCAGTTGGGAGTCAGTGAAATTAGCAC	A/G	3	82915876	0.966
3084726779	ATCATATTTGTTTTACTTACTAGTTTTGAAAGTTGAAAACTTGACATACAAGAATACGTG[A/G]AGATATGGAGACAGTTTGAAACTTGTTGAGATTTTAAATGATCCATTTGCACAAAATTG	A/G	3	84726779	0.817
3086289128	CATCCTTGCCAACATGTTCTGTCACTTGAGTTTTTGATCTTAGTCATTCAGTCATTCATTT[C/G]TATATGACTAAATGATCATGATCAACAAATTATATAGAAAAGTATGTCTGTGGTTTATGTA	C/G	3	86289128	0.748
3087797914	ACTCTTATTCTAAGAGCACAGTCTAGGTACAGGAGGTATTCCTGGCTTTTGGTTTGTCATT[A/G]TTAATTAGTGATTATTTAAAGTTATTTAACAGGGCCTGGGGATTTAGCTCAGTGGTAGAGC	A/G	3	87797914	0.689
3089363019	CGCTATAATGGACAAGGGGATGGTAGCGTGGAAGGGTGAGATAGGAAGTGTACCAGACCAC[A/G]TGAGACAGAAGCATTTCTGAATGAGAGTGTCCTGGCACCCGGTGAGTGTCTCTGCCTGGCA	A/G	3	89363019	0.929
3090974446	GCTAAGATATACTACCAATGAGTCATGGTTTTGTCCCATTTTTCAGTTTTTAAAGATTCTC[A/G]ACCAGCAAATGTCACATAATGGGGAGTTTCTGGGTTGATTTCCAATCTCTTTCTTGTTCTC	A/G	3	90974446	0.953
3092549463	ATCTGGGAAAATCTGAAACCTCCCTCTGGTCCTCAGCTTCTCTGCTGATTCTAAGGAAACA[C/G]TTCCCAGGATGATTTCCCCTCAGTGATGCTGGTCTCTTCTTAGCCACGGCTAATTTCTCAA	C/G	3	92549463	0.786
3094071447	GTTCACTTTGCCTCTTGTTTACTCTTTTGAAAATGGCAAATAAGAAAGCTCCATGTGGATT[T/C]TGGAGGCAGACAAACTTCTTCCAAGTGCAATCAAGAATCTCTATCCTCTCTTCAGTCAAAT	T/C	3	94071447	0.689
3105451747	ACTGTGTGTCGAAAACTTTACCCATTTTAGGATTAGGGTCAATGGTTTTCAGCTGGGGTCC[T/C]CTTAGGGGATTTGGAAATAATCAAAGGGGTAAATTTCAAAGAAAGCTACACAATTTTTATA	T/C	3	105451747	0.955
3112264059	TGATTCTGTTAGATATTTTGTCACAGCTATGCACTGAATAAATAATATAGTATATATCAG[T/C]GATGTTTTGATGTCCTAAAGCCCAATAACCCAGAATATGACTGTGGTTAGATTTGGCTA	T/C	3	112264059	0.648
3115483838	ACATGTACCACAGGGCTCATATCAGGGTCATGTAGCATCAGAGGACAGCTTGGAGCAGTC[A/G]ATTCTCTTCTTGCACTGTGTAGGCCCTGGGCACGGCGCTACAATGCTCACACTTGGTGC	A/G	3	115483838	0.864
3117024540	TGGGGATTGAACTGAGTCTTCTGTGTGCACGGTAAGTGCTAAGCTATGCCCTGATCCCCA[A/T]GGTCATAGGCTTTGTTTTTTACCACTGCTTAGATGAAGCCTGTCTTCATTTGGGGGTTC	A/T	3	117024540	0.98
3118842691	AGGGACTTCAAACTCTGGTATTGGTCTGCTCTTAGAAATAATCATGGGACAGGGTCTTAT[T/C]GGAGCCCTTGGCATCTCTAGGACTCCTCATTGGCCAAAAGACTAAAGAATGGACCTCGG	T/C	3	118842691	0.956
3120434693	CATTTCCTTGCATTTGCATCCACTAGTGACTGGTTCCTTAGTCACTTTGCTGCTTCCTTC[C/G]ATTCAAGATTCAAGAAATGCCTTCCATTTGCTCTTCGATGGATTTTCGATTTGGAGGCT	C/G	3	120434693	0.918
3123525182	GTATTTGATGCTCTCAAAATGTACATATTTGGGTTCTTCTATCTCAAATTATTTAAAATGC[A/G]TAATTCACATTTTTTTGTAATCATTCTATGCAATTTTGTGGCATGACGTTTCTTCCACTTG	A/G	3	123525182	0.595
3126820406	GAAATATTTAATCAACATCAGAGAATGGATCTCAGCAAAGAAGTCACCTGCCTGAGGACAC[A/T]TTTTCCAGCGCAGTTCTTCTGCTCCATATTCTGCTTTATGATAGCCTGGGAAGCACACAGC	A/T	3	126820406	0.741
3128386941	AATATTTAGTGGGAAGACATGAAAAACATTTCACCCTGGATAAGGCACCAACAACAGACCA[T/C]CCCAGTGTCCAGCTTGATGAGCCAATGAATTACTGGGCTTACTTAAAGGATAGTGGGTGAC	T/C	3	128386941	0.86
3133286498	CTGTAGAGATGTCTGTAGGACCAAACAGGGTCTGAATGACGTGGGGGCGTTCAGAGTTGG[A/C]CATACAAGAATATGTGCAAAGTGGATCACATACTTCTGAACAGCGTGTTGTTTTGTCTG	A/C	3	133286498	0.952
3136767098	CCTCCTTGGAGCTGGTCAGCGGTGTTTAGTTCTTTGGTGGCTTCCTCTCTCTCACTGGCG[A/T]GAGTGGATATCTTACGTTAAATTTTAAGTTCTTCTTAAGAGTTACATTTCCCCCCATTG	A/T	3	136767098	0.905
3142347974	AGGGGCCACCTCCTACCCCTCAGACCTCAGAGGAAGGACTCCTTCCTGATGGCATCCTTCG[A/C]ACACTGCTTAAGGTTACCCTGCTGACCTCATCTCCCTGCAAGAGTCGGAGCAGGATCCATC	A/C	3	142347974	0.897
3143920365	CGTGGCAGTAGCCTGCTCTGTGAACTAATCTGACCACCCCTGACAAACAGATGTAGGAGAC[T/C]GGAGGGCACTCGCCCTTGCTGCTTGGCTGTGTGACCCTAGGCAGAAGAATTGACATCTCTG	T/C	3	143920365	0.896
3147613796	GGGTTCAATGCAGTCTTTTCTCCATTCTGAATTCAACGAGCCTCTATTCACCCATGGAAAC[A/G]CACAACACGGGACCCCCTTCTCTAACGCTGCTTCCTATACAACAAACACATTAACTGCTCC	A/G	3	147613796	0.852
3149186732	AGTTAATCAGCAGAACTTACCATTTTCTTTGTTGTGTTTTGCTTTATTTTTGTTTTAAAAT[T/G]GAAAGAAACTTCATTTTGGGGAAATGCTTTAAAAGTACCTGTCTTATTTCAGTTAGAAGAG	T/G	3	149186732	0.689
3156964356	CATCCCCACCAGCTCTGTTCCGGCCCCGGTGCCCACCCTCAGCTCCTTCGGTGCCTCACTC[A/G]GCTCTGCACACCCATCTGTCCGGTCCACCTGCTAGCACATCCGGCCCCTCCTCTGGCCCTT	A/G	3	156964356	0.809
3161912878	TGCACCGGGCTTTTACATTTTTTTCTAGTTCGTATGTAGAAGTGTTTGCCTGCATGAATGT[A/T]TGTGCACTTGTGTGTGTCATGTCCACAAAGGTCAGAAGAGGGCACTACATCCTTGGAACCA	A/T	3	161912878	0.715
3163501339	AAGCTGCCTTCGAACGTGAAGCCCTCCTGTCTCCGATATGGGGTTACAGATATGAACTACC[A/G]GAGCAAGTACCTATACTCCCTGGGCCATCTGAGCTGCCCAGGTCTTTTAAAAAAAATTTCA	A/G	3	163501339	0.85
3165014088	CCTCTGACCACCTCCCAATGCCTGTTACTCCCTGTAGCTGGCTGATGACCGCATGGCGCT[C/G]GTGTCAGGCATCAGCCTAGATCCAGAAGCAGCAATTGGCGTGACAAAGCGGTCCCCTCC	C/G	3	165014088	0.776
3168152874	TCTGTTAAGATGCCGTTCTCTAATATTGATTCTCTGTGCACAGAGGTCACAGGTGACAACA[A/G]CTGTATTGTGAGGCTTTTTAAAAAAGATTGTGATTATATTTTATTTATAACTGTGTTGGGA	A/G	3	168152874	0.891
4001510132	TGGCATATCTTCTGAGGTCTTCCTCAAATGTCATTCTCAAGGGCATACCTTTGCCTGGTTT[A/G]AAGTTGGTACTTCCTCAAGGCTTTGGCTATCACTGTTTCAAGACTAATGAGTCAAGGCAAC	A/G	4	1510132	0.849
4003183259	CGTGAACGGCTTCCGGGGTCTGCCTCCTCTTCACAGGCTGCCATCTGCACTACTTCTGAG[T/C]GTTGGTGGTTCTGGAGAGCCGATGTAGACTCACTTCCGTCCTAGAACTCTGAGCTGACC	T/C	4	3183259	0.889
4004770797	ATCCCACGGGTGGCAGGAGAAAGGTGCCATTCAGGGCACCTCAACCACCAACAAAAGCCCT[A/G]GAAACCAAGGGCTTAATGAATCCAACCAACCAAGGAGGGCAGTGGTGGCTGTTCTGTGAAG	A/G	4	4770797	0.94
4007954708	TCTGTCCTAAAGGTTGTAAGTAAGCCTTATTGCCTAGTCACTATTAATGGTGTGTGCAGC[A/G]TGATCTTGGGTATGTAAGGTCATATTGCCTATTACCTTTTCCTCGGTATTCTCAGGCTC	A/G	4	7954708	0.911
4011087981	AGGCAGGGTGGTACTTGTAAAGTAGAGACATGTGATTTGTAATTAGGGCATTTGGGTACA[T/G]TGTGCTCAGGGCAGCTCAACGGAACACGTGTTCCACTCATGCAGGGCCCTGTTGCATGA	T/G	4	11087981	0.558
4014153602	ATATTTATAAATATTTTATATGAATACTAATAGCTGTTATGAGTATTCTACCCTCATGGCC[A/C]AAACTTACCCTAATGATTGTCAACTAGTGCTACCATCATCTTATGCATCATACATTTTTAA	A/C	4	14153602	0.91
4015706671	TCACGCACTCCGCGCACAGCATGCTGGAGACTTGAGTGTAGATTATCTAAAATCTCAGGC[T/C]GGTCCCTGTTGGGAAGAAAGGCAGCAGAGGGCAGAGAGCTCCATGCTCTGCTGCCTCCT	T/C	4	15706671	0.904
4017323924	ACCTAGCTGTATACTAAGAGCATATCTAAAGTCAACGCAGAGGGTTTAGAGAGTGTGCTAC[A/G]ATGGATAAACACCTCAACAACAAACAGTCTTTTTGGTAAACACACCATTTTGGGGGTTCAC	A/G	4	17323924	0.908
4018914851	CAAATAAATTATTAGTATAAAGGCCTCCATAAGGCTCTGTACTTCCCAGAGAAGGGGTCTG[T/C]TCACCATGTTGGATTCAGAAGGATGCTTACACAGAAAACAAAAGCACCTAATACCCCCTTG	T/C	4	18914851	0.896
4021988111	CTATACAAACAACTAGTTCAGCTGTAGCGTTTTGCGGTTGGCGGTCTTGTAGGAAGTGAAT[A/C]CTTGGTAACACGGTTTCAAAGAGAGTGTAAATTTCTGCCTGAACAATCGAACCAATAATTT	A/C	4	21988111	0.905
4023502835	CTTATTACATTAGGTTTTTCTCACCAATATTTATCAAAGTGATGCCTTTCTCCAACTTGT[A/T]TTTTTTTAGTAATTTTGTCAAAGATCGCTAGCAATTTATTTAACAACTTTATATTTTAT	A/T	4	23502835	0.882
4027941759	GGGATTAATTCCTCAACCAGACAGGATGACTTAAGTTATGGACTCTGAACACTGTTGAAA[T/C]TACCTAAGAGGAAGAATATTGGTCCAAAAAAATGCTGTGGCTGTTGGTCAGTGGGGCAC	T/C	4	27941759	0.686
4031072166	AGATAGAGCCCTTGAAGCTGCCTCGTGACTCTGAGGCGTTGGATCGTTGTGTCCTGGTCAC[C/G]TGTTCAGTAAAAAGCTGGAGAGCCCATGGCTCGTGGGAGACAACCTGTCAGTGCAATGTTC	C/G	4	31072166	0.959
4035890150	CTGTAATTGTGTTCATACCATGCATGTCCCATTTCGTCCGGGTTACCTCACTTGGGAAAAT[A/G]TTTTCCTGTTCCATTCCTTTGCCTGAAAGATTCATGATGTAAGAATTGTTTTAGCTATCAG	A/G	4	35890150	0.759
4037413372	CAGAGGTGTTCATATTATATTGTATTATTATAATGGTGTGACAACTATTCAAAAGCAAGG[A/G]GATGGGATGATTCTAGATCTTCACCAACTGATTATTAGATAAGAAAATATGATACATAC	A/G	4	37413372	0.942
4039060333	TATACTCCACTGACTGCTTACTGCTGCACTCATCTCAGACCAGTGCAGGAAGTAGAGAAG[T/C]GCTCCAGTGCATTCTTAGTCCTCTTAGAATCAGAGAATCTGTTTAGTCTGTGCTTTTCT	T/C	4	39060333	0.911
4040902321	TGTGGTACTGGAGAGTTCTACATCTAGATCTGAAGAGAGCAGGAAGAGAGAGCCACTGGGA[T/C]TAGTTTGTGTTTCCGAAAGCCCAAGGGCCACAGCAACACAGGTCCACCTACAAGGACATAC	T/C	4	40902321	0.502
4045685149	TTATCCCCCTTCCAATTTCCCCTCTGGAAACCCTCCATGTCATTCCATAGTTCCCTGCTCC[A/T]ATGAGGGTGCTTCCCCACCCACCGCTGATTCCATCCTGCCTCCTTGCCCTGGAATTCACCT	A/T	4	45685149	0.602
4047510440	CCTCCAGGTGCAACTGTGCAATATGGGTACGCCTGGCAGATGTTCACCTGGCTATGGACC[A/C]GGGGGCAATGAAGGGGCCAGAAGAAGACCAGGGCCAAGAGGTATAATGTGCATATTGTT	A/C	4	47510440	0.609

4049098430	CCACCTAGTGTTTGAAAAGCCATCAAATGCTCTATCTTTGTAGAATTCCTGAGCCAATTTA[T/C]GTCCAATCAGAGTGTTAGCAACAGTTGTAAATGTTCTGCCAAATAGATCTCTAATCTTGTC	T/C	4	49098430	0.876
4050715446	TGGAAACTTTTAAGAAGTGGATCCTAACGAGAAGTGCTCGAAAAGGGCAGAGTGGGATCA[A/C]CGGCTTCTATCTTGCTCCGTGACTCATAAGAGGTAAGGGTTTTTGCTTTTCCACATTCT	A/C	4	50715446	0.824
4052313040	GGGTTTTCCCACGGTGTCAAGTACCTTGACTGGTCTTTTACTGTGAGTCATCATTCTGTCA[A/C]TGTGAGAAATAGCCAAGACAATTGACTTATACCAAAACAAGGGGTTACATTGGTTTATGGT	A/C	4	52313040	0.838
4055570079	TCAGGAGGAAGTGACAGCAGTGATACAGATTGGTTCTAGTCAGGCATATGGATCCTGCGG[A/G]GAAGCACACTAAGGATAAACCTCTTACTTGGATGAGGAGAGTTAAAGGTATGGAAAGGA	A/G	4	55570079	0.976
4057174499	GGAGTTGGTTCTGAGTGGCTGTATTGGTGCTGGGATCTAACCCTGTCCTCTCCGGAAGAGC[A/C]GACAGTTCTTTTTGAATTGCTGAATCGTCTTTCTAGACCCCTTTATTTTCTTACCTTAATT	A/C	4	57174499	0.956
4059071691	ACATAGGCAGGCTAGAATGATTCAGCCTGGAGCAACAGACTCATACCAATATAAAGCTAA[T/C]GTTGGCTTGTTAAGAACAGATTCAATAGAACTTCCTTCAGTCGGTAAAGCCAGCTTCCC	T/C	4	59071691	0.823
4060670736	TAGTTACATGTATGCTACAGTATACCTATGGAGTTCTGATGTCAATTGAAGGAGTTAGTT[T/C]TCTCTTCCTACCATGTGGTGTCCCTTACTGAGACATCTTGCTTGCTTTAACGTTTGTTT	T/C	4	60670736	0.986
4062198189	AAAGATTAGTAAAGCACACCTCTGGGTATAACTGATAGGACATTTGAAGAGGATTAGATC[A/G]TGAGGGCTGTAACAGTTCATTCTATTGACAGATTCAAAACTTGGGGCTACTGGGAGATG	A/G	4	62198189	0.908
4066812933	CTAGGGTTGTAGGCATGCACTATCCACACTCACCCTGATGTAAGTTTGGCTAGAATAGACA[A/G]AAGAGCTCACAGCCAAGTGAAAGAATGGAGTCCTACCTTGCTGAGGGCAGGAACGCAGGGC	A/G	4	66812933	0.955
4068343084	TGCATTCCAGCACCATTACATTTACAGCTTCTCTAATTGTTAGAGCATTGCAGGCAGACTG[A/C]TGGTATTAGCCGTGGTAGTGAGGCATGGTAGTGGGGGGGAGCATGATGGTTGTGATAGGAA	A/C	4	68343084	0.943
4069863554	CACATCCAACATAGCTGATGCCAGTCTCTTCTGGAGTAGTGTTCTCAGAATCAGTCTGCC[T/G]GTCTACCCAGCTGGCCACTCAGTTTTACTGATTCAGAGCAACCCTGGCTTAAAAAGAAC	T/G	4	69863554	0.982
4073009704	CAAAATGGCTGAGTTTATATATGGAGGAGAAACTTGGAGAAGGGAAGTGAAGCTTCTGAA[T/C]TGGAGAGGTTGAGAGTAGCAGACAAGGCATGCTAGCTAGGTTGGCTCACTGTCGGTAGA	T/C	4	73009704	0.937
4077641301	TCTCTCAGCAAGCAAATATCAGTGAAAATGAGAGACCCACAAGCTTTACATAACTAGCTCT[A/T]TAAGCAAGCCAAACTCTGACACCATCACTGTCCCTTGAGTCCTTTTATACCTTCCAAACAT	A/T	4	77641301	0.879
4079183914	AATTTGAGATCTGGACAAAATTGGAGAACATATACTTTTTTTTGGATGAGGTCTTGAGATG[A/G]AAGTCCAAATTGTTTTAAAGGTACTGTTACCTGAGTCACCAGGCCCACCCACAGTCTCCTG	A/G	4	79183914	0.93
4080742511	TGTCCCATTTCACAGACAAGGAAAACTGAGGTTTTGCATGTTAAGTAACTAGCTCAAGAC[A/G]TGAAAACTGAGGTAGAGGGTATTAAACAATTACCCTGAGATAGAGTAGTCAATGAATAA	A/G	4	80742511	0.816
4082266250	TTTCCTCCCACATAAACATAGCAGAGAGAATGCCGGGACAGCTTGCTTTCTCCCACACAAC[A/T]CAAAATTGACATAGAAAGGATTTAGAAATATGGATACTTTCTGTTCCCAGTGACCCCATTT	A/T	4	82266250	0.961
4088644702	AAGATGCCAAAGTGAATTACTGAAATGGAGAGTTGAGAAAGTTACAGAATAAAAATAATGT[A/T]GATCATTAGAGAAACTACATTTCAGAGATTAATCTAGGAACCATGAACACTTTCAAAGTAG	A/T	4	88644702	0.567
4090207324	AACAATTAAAATGTACTGAAAATTTCTTAGTGATTTTGAATATTTGCAATAGAGCTAATC[A/G]TCTTTTCTATTTAATATGTTTCCAGGATCTGAAACATGAAACATGATCCATGAAAGAGA	A/G	4	90207324	0.631
4091870538	TGCTTGCTAGGCAAGTGCTCTACCACTGAGCTAAACTCCCAACCCCACTTCACTATCTTTT[A/G]GTCAGGAAATGAACTGATTTCAAATCAAATATCCTTGCACTTTCCCTTCCATTTCACAATT	A/G	4	91870538	0.943
4093383134	GATTATATATATCTAATATACTGAATCTACCTGAAATCTACACATTCTAACTTTAATTCC[T/C]CCATCTCTTCTCATGTACCAAACCCCTACTCTGGTTCAAATATATGTCCTCTTTTGTTT	T/C	4	93383134	0.808
4094931788	GGCATGTCTGACCAATTTTTTCTTTTCTTTTTCTTTTTTCATTTTTGGTTGTTTTATCTA[T/G]CTAGCTACAGAATCACTGTAAATTTTAAAATATCAGTTACTAGGACTACTCTAACACAT	T/G	4	94931788	0.539
4096920596	ATTGATAATACTATTATGTTGCAAAGTGAAAAGTATAAAATTAGTAAAGTGAGAACCCAC[A/G]TGTTCCATGAGAATTTACTTATATGAAATCATAAGTATGCTTGACGTGGTAGCACATAA	A/G	4	96920596	0.676
4098619123	TTCTGAAATGTTTTATATTGCTCATAGTATAAAAATAATAAGCTGACCTGAAGCCCCAAA[A/T]GTTTATAGTACATTGGACAATGAGGTCTCACTATATGATATTTTTTGTTACTGAGATAA	A/T	4	98619123	0.835
4103249461	TTTAAGTCTTACAAGGAAGAGGGATTTGTATTCTGGCCAACATGAATTCAGTTTGCTTTT[T/C]CCATAAGTGGAAAGTGAAGCTAAGGAACCATTTGATAATTGTTGATTGTTGAGTTGACC	T/C	4	103249461	0.872
4104814037	TATACATGTTAAGAATTAGTTCTGGGTAGAAATGAAAGAGATCACCACCACTCCTGAAGC[A/T]TGTCATGTCTGGGCAAGGCAAACTTCTATTATCAACCACGAAGGTATGCTCAGAAGAGT	A/T	4	104814037	0.839
4109637514	TAGGTGCTGTGTCTGATCTTTAATATTCTGGAAGGGCTCCACGTGCTTTTAACTGCTGAG[A/C]AATTTCTGTAAACCCCTTTTTCCTATAATCTATTAAGACAAATTAGAGTGTCTCTGTAT	A/C	4	109637514	0.8
4114563241	AAAAATAAGCAATAGAAAAATATTTGTATGAACAAATGTGAACAAAATGTGAAATGATTT[A/G]TTATTATGCAAGTTTGCATCCAGCCTCAATGCTATATTAATTCCAAGTAGTGAAAATGC	A/G	4	114563241	0.955
4116166995	GATGTACTCAAATACAGCTCTGTCGGGCTTGTAAACTGTTCATCTAATGTGTTGAAACAAC[A/G]GAAAGTCCATTTATCCTGTCCCCAGAACTCAGCTCTCAACTCTTCCCATTAGATTCTCTTT	A/G	4	116166995	0.974
4119360890	AGTCCCTCGGCCAAAACCTGCTGTTCTTCCAAAACCAAGCCCTACTGTAGGACCTGACGCA[C/G]CTTCCCAGGGTCCCACAGTCAAGTCTTGCACGATGTAAAAACGAAGGTCGTGAGGGGAGGG	C/G	4	119360890	0.782
4124297354	CTGGGACTGGACACTTCCAGTTTGTAGCTGGAGCCCCAACCTAGCTGATCCCAGCCCTCAG[T/C]TCACTACTCCCAAGTACCTTAGGAGAGAGAGTTCACCGCCCGGACAGGTGGGCACTCCTGA	T/C	4	124297354	0.549
4127749824	TTAAAAAAGCATACTTTTCGAGACTAGGAGACAAGTCTGCTAAATTGTCTTTTTGAAACCA[T/C]CCACGTGCATCCCATGCGACTTTTAAACACCTCTTCAAGTTTCAGTCCAGCCTGAAGACAG	T/C	4	127749824	0.88
4129411553	CACACACACACACACACACACACACACACACACACACACTGTCCTTTGTTCTTTGGTTTAC[A/T]ACTTGGGCTGCTTAGGCCTTTGCTTTCACCCTTTGAGTTCAGAAACAGAGAGGACCCACAG	A/T	4	129411553	0.982
4131221730	TCAGAGTCATAGAGACAACCCTTCAGAGGGCGGGTAAACATAACTAATAAATCAAGCAGAC[T/C]GGGTTGGGAGTTTCGAGGGGCTATTGGGAGACAGTGGCAAACTGTTCAGATCATGCCTGTC	T/C	4	131221730	0.898
4133375390	TATTTTTCATAATTATAAGCCCTCCCTAACTCCCACTCTCTAGCTGGATGTGTAGACAAG[T/C]TGACTATCCTATAGCTTCATGAATCTTTTAGTTTTAAAAATGACAGAAGTTCATTTACT	T/C	4	133375390	0.841
4134899865	GGGGTCAAGACCAAGAGCTCACTGAAGAGCGGGTCTCAGAGGAGACTGGTTACAGCCTGGG[T/C]GAGGGAAGAATGTGTATCAGCCTCAGTCATGAGGGCTATTCAAGTCTGTCAACCGTGGACC	T/C	4	134899865	0.905
4144487081	TGCTGAAGACATTGCATGCTTAGGTTGCAGGACGTGGAAAAATTGAGCTGGAAGCTCTCC[A/C]TGGCTCTTGTGTTCTTTAAGCCTCCTCTTCCTCAGGGTTCCCTGAGCTCTCAGGGGAAG	A/C	4	144487081	0.806
4146021397	TTCTAGAGTAGAATTAATAAGCATTTAAATTATTATCATTTTAATGATTTTCTTTAAGTAC[A/G]CTCTCCACCTGTCAACTGAACCAGCCATGTAGTGATATTTCTCCAACTTCAGTCATAAGCT	A/G	4	146021397	0.828
4147671667	ACCAGTGTTGGGGTTCAGAAAGTGACATCCCCCATATTTGCTCTTTGATGAGCTGACAAA[T/G]TTGAGAGGTGGCCTCAGACCAAGATCCCCAGGGTCTTCTTTTTTCACTTCAAGACAGTG	T/G	4	147671667	0.896
4152505867	ATGTGTGGAGCAGGTATTGCTGCACTCATGGAGGATGGAGGATGGGATGTCAGGGGCCTG[A/T]CCAGCTGTTGGCATGCTCCTACCACATGTGCTCTCACGCACTGGCCGCTCTCTAGCCCC	A/T	4	152505867	0.625
4154143910	AATACAACTATTCACAGACACCCCCACTCCCAACTCCAGAGAAAGGAGGGAAGCAGGGGA[T/G]CCTGCGGCAACATTGGATCCAAGAGATCCCAGAAGCCCACAAACTTTATGTTTAACCTG	T/G	4	154143910	0.7
4155805092	TTCACCTCCCAGTATGACACAACCACTCCCTGTTTGGTTTGGTTTGGTCTTACCTTTGTT[T/C]GAGACATGGTGGAACACCAATGATCTACGATGCCATTTAGATTTTTAATGCTCTCTTTT	T/C	4	155805092	0.762
4157312410	CCGCCCCCACTGTCCAAGTGCTGGGGCTACAGATGCCAACCACTGCGCCTGGCTTTTGCC[A/G]TGCTGGGGACTGAAGTCCTCTGCCATTCTACTGGCCTTCTGCCCCTTACTTGTGCTTCT	A/G	4	157312410	0.675
4160402821	ACAGCTTGGATGTGGCTCTGCACTTCAAACACAGTCTAAGATCTACTCTAAAACCAAAAC[T/C]GAGGCAACAAATACGTGGCTCTTTCCTGGCTCTTGCAGTGGAAATGAAACTTCTCTTTG	T/C	4	160402821	0.902
4162009988	AAAATTAACATATGAGAAATATCCCAGGGGAGTCAGATAGTCTTTCATAGTGCTGAACTCA[A/G]GGGCATGAGCAAAGACTGCTGGAGGTGACCTGAACTCCTTCTTTCGAAGCACCAAAGACTC	A/G	4	162009988	0.966
4163821233	AGTAACTCACCTGCTTCTCGAATGGACCCACTTGAGGGTCCAGGGCCACTGAAGGGTCTTC[A/G]TACATAACTGAACACTGTCAAAGCTAGACTATTCATGTTTTAATATTCAAAGGCTTAGCAG	A/G	4	163821233	0.991
4165454560	TGATGACCTGGGGGGAAGGGAAGAAGGTGAGTACCTAGGGATGTGGGCAGAAGAGCCCCA[A/G]CTGTCCATAAGCCCCACCAAAGGCACAAACTGGATGGCCTCTGTTGTACCCACTGCCCC	A/G	4	165454560	0.65
4168756464	TTGGCACTCTTAGCAAATAAAAAACTGTCAGCCACAGGATGTACCATAACAAAGAGTTAAA[A/C]TTCATCAGCGAAAGATATGCTTTCTGAGATATTCTCTTGTAAACTTATGAGAAAGGGATAA	A/C	4	168756464	0.742
4170447191	ACCACATTAGGTGTTGCTCCAATATTTAAAGCACATTGAAAATCTCCCACACTCCAGCCT[T/G]CTGGGCTACAGTCCCAGTGAGAAATAATGAAGCAAGGTTGCCCAGTTGATGCTCAAAGA	T/G	4	170447191	0.614

4172050247	TTGTTTAGCTGTAAAGGGCGCCAGCTGGTCATTGGCTCGCAGTCCTGTGTTCCCAGTTTC[T/C]GGCTTAGTCCTGTTATCGTGGACCCTGATTCAGCAGCAGAGGCATTCCCGGGTTGACTA	T/C	4	172050247	0.935
4181963967	GACTGCTATCTCTAATTAAATGTAGAATTAATTTTGGCAAACTAACCACAGATGCAGTTTA[T/C]GCTCAGTTTTATCAGACAGATTTATTTATAAGCTGTCAACTTCTTGAAAGACTTCATTTTG	T/C	4	181963967	0.872
4183540141	CTTCCTCTTGTCTACCTTATCTGTGGAGACCAACCTCCACAGTCTACCTGAGAAAAAAAT[T/C]ATGAAATCCTTGATGGAAATCACAGGTTCAACTTCCTGGAACACCTCTCCATGCAAATG	T/C	4	183540141	0.921
4185056116	TTCTAAAATGATGTAGTGGCCGTTCCGGAATGGAGAGGCAGGGAGGGAGGGAAACAAAGC[A/G]GAAGTCTTTTAAAACTGTAGCTGGGGAAATCAGGCGTTCTCAGGTCAGTCTGCAGCCAC	A/G	4	185056116	0.72
5005067054	GATCCCTCCTGTACTTCTGAAAACATGGTTAGAGTGCAGGGAAGATCGGTGGCTGTCTAC[A/G]CAGGAGGTGGGTAGAAAGCTTCCAGTTTACCCCAATCTTTTAGGAACCAGGTAATTATT	A/G	5	5067054	0.896
5006609907	AAAATCAGGAAAGCTGCAGTGAGAAAGAAGTATTTTAGAAAAGTGACATTGATTTATTTC[A/G]TATTTTTTAGAATTAATTTTACTATGGCATCCAATAGGAAAGATGATCACCTTAAAGAA	A/G	5	6609907	0.552
5009771971	AACAGTTGTGAGTGTGTAGCTCTATTAAAAATGAGAAAAAGGAGAAGCCCACTCAATGGA[A/G]GGACCAGCAACCAGGTGCATCTACAACCCTGACGTACTTTCCAGGTTAAGTTTTATGAT	A/G	5	9771971	0.745
5011423376	CATTCTATCCCCAGAATATTGCTAGATAAAATTAGTTTAAAATATTAGCATTCCTGGGAG[A/T]GTCCCTTTATTATTCTCATTTGCCAGAATAAGATGAAATAGGAGGAAATTATATAACTT	A/T	5	11423376	0.9
5014587097	TTTAGTGACACCCAAAGTTTGTCCATGTTCTGAGTTGATATAAAGCCACTCTGTAGGGCCC[A/G]CTGCTTTGTTCATGTTCTTCTTGCACAAGTTAGGAAAAAGTAGCAACATTTCAGAATCACA	A/G	5	14587097	0.934
5021092679	GTGTGTGTGTGTGTGTGTGTATGAAAGTAATCTAACTGAAATCACCAAATATTGTTTGAGT[T/C]AGAGCCCTAACAGGCCATCTTTTGTCACCCAATGACATTTCAAGTCCAGAATTAGGTTATG	T/C	5	21092679	0.987
5022847270	TCCCAGACACACTGCCTGGGAACAACAACAGGACGTCTGAGATCAAGACTGGTTCGTTCTC[A/C]GCATTAAGTTTCCTCGGAAGACTTCCACGGTCGGCGCTGCTGCAGGAGGCCACGCAGATGC	A/C	5	22847270	0.93
5024525099	TTGAATGAAAATGAAAGCAAAACACAATACATCCATGGAACACAGCAAAGGGAGTCCGAA[A/G]AGAAGCGTTTCTATCTCTAAGTGCCTACATTGAAAAACCAGAAATGTCTCAATATAGAT	A/G	5	24525099	0.991
5026080053	AGGCTGAGTCAATGCCACCTACGTCTGTCTTATACTTCATGAATGGATGTCACCATTACAG[T/C]GTACTGATGAGAGAATGATACAAGAGAAGGCCCAGAGGTCTTGAGTCTTTGAGAACAATAC	T/C	5	26080053	0.919
5027598108	AGTCCTAATTGAACTTCATCTTTGCTGTGACTGAGACTCAGATAGAAACAAATGCAAAGT[T/C]TTATTTGCTTAATCTGTTTACCAGTAACTTTTCATTCTTGCAGCGTGTGCTTTTGTAGT	T/C	5	27598108	0.807
5029291630	TAAACTACTCTTAAGGGTAAGCTAGTGTTAGATGGCTAACAGAACATTGATTCAATGACGT[A/G]GTTGGAGGGTCCTTGTCTTATAACATCCTGTTTGGGATTTTTTTCTCTTCTAAATTTTATT	A/G	5	29291630	0.877
5030844009	GGATCCTTGTAGTTAGTCCTTGAGATGTAGATCATAGAGCTGCCAGGGTAAAGTGAAAGAG[A/G]TAGTGTAGAGCTGAGAAAGGTAAAAGTGGAACACCTGAAATTTGAAGGCCCCAGTAGCCAT	A/G	5	30844009	0.837
5032389662	ATAAGAATAGTGAATGATACTGAGAAACGAATAATAAAGGAGGGAAAATTTTGATAAGAC[A/G]TATTGACAAGAAGGTGAACATAAAATTTCATGTTCCTCAATTTCACATGTTAGAATATA	A/G	5	32389662	0.81
5033983990	TTTTTAAAATGTCAACAAATTTCAAGGTGATAAATCCAATTATGTCTTGTTCCATGTATG[T/C]TCTTACAAGTAGTCTTACCCACAGATTAACTTCTATCATCTCTTCTAGCCAGTGACTCC	T/C	5	33983990	0.862
5039065030	TAAAGCTATTTAGTTTCCCAGAATACAAAATTTTGTTTTGCTTTGTTATTATATTCCTGA[T/C]GGCAGGAATCATGCAAACAACAGGAAATAATGAATTTGACTCTGTATCTTATAAGGTTG	T/C	5	39065030	0.64
5040663200	AATAATCTGCATTTTACAGTGACACACCATCTACTAAAGTTTTCTAAACCTTCTCAAATC[A/G]TACCAAAATCTATGTTTCTATTAATGGTTCAAATGGATGAGTCAGATAGGACATTTCAC	A/G	5	40663200	0.613
5046890247	ACAGACTGATGAATCCCACCATTCCTGCAAGCAGAAAGACCGGAAGAAAGCTATAGGCAT[A/G]TTTTTTACAGCATCTTTCCTGCGTTTGCCAATGGTTGACCTATCATTAATGGTCTGATG	A/G	5	46890247	0.904
5048449425	AAGTACAGAAGTTTAGATCATCTGGGGTGAATGTCTGGGCTATTTCACCAGGCAAGCAACC[T/C]AGACCAGTAAAACTGCCTCCTGAGGAGATGGACAGCAATCAGGGACCTGGAGCAGCATTGA	T/C	5	48449425	0.905
5050017626	ATGCTGAACCCCAACTCTTTGCAAGAGAAACAGTTTTGCATAGGTAGAGAAACCCAGTGAT[T/C]ATCCTCCCTACCTCTTTGCGGTATTTATGTGATATACATCTGGGGTCAGAGATTAATGAGA	T/C	5	50017626	0.986
5051833536	TAGATGTAGTCATGTCTTAAGCGTTGTGCACATGGGCTTGAGTGAATTGTCACCAGGAAAC[A/T]TTTTAAAATATGACTAAAATACACTGCCTCTAGATGTCAGCCTCTAGAAAAGTGAAGCAAC	A/T	5	51833536	0.765
5054983065	AAGCAAAACTATTTGCAGATAGTTGGTATGTTCAAATCTTTCCAGGTCTCCTTTCCCACA[T/C]ATTTTTCTTCAGACACAGATATATTCTATTTTAATAAATATTTCAATAAAAACATTTCA	T/C	5	54983065	0.846
5059722864	GGACAACTTCCCACCCCCTTGCTGTGAGTCAGCAGACTCCACCACTAGTGGAGAGAGAGAT[A/G]GGGACCTGGCCCAACTTGGTAGCTTTGAGCAGCAAGCCAGTAGCCAACCTTCTCTGGCCTG	A/G	5	59722864	0.915
5063069411	TCAGAGAAGGGATTGAGAGGAATTGACCAATGCCCTGAAGAGCAGGCAAGGATGGCTGGG[T/C]TTAGAGTGCTGGGGGAGGGGAGTGTGGGAAGAACCCTACCAGGAAGCTGCCTGGGCTGT	T/C	5	63069411	0.553
5066241199	ACGCTAATTATGTTACCTAAAAACCTGTTTGCATTGTCCTGCATGTGTGACTAAAGGAAG[C/G]CTGTCCAGAGTTGGTTTTTGACTGGTCAGTAAAGTTACCAGCTGCTAATGGCTGGACAG	C/G	5	66241199	0.648
5067878089	GGTCCCACTTAGGCCAGGCTTTAGGTTGGGGGTATCTCACTTGTGAGCGTTGTTGTGAAG[T/G]AGCTCCCAGGAATTAAGCTCTCTCTATGTGTGAGAGGGGATCTGGAACACCAGCTCTGA	T/G	5	67878089	0.873
5071110542	GTGTGTGAGAGGTTCTTGTTGATCACTTCCGTTTCACTAATGGCTGGATGGTCTGAATTT[T/C]TCCATTTTTATTTATGGTAGAGCATTGTGTATAGGTCAGAGGACAGCTTGCAGGAATAG	T/C	5	71110542	0.957
5072889821	CAAAGTGAGAAGTTGATGATGTAGCTTTTAATGGCTAGTAGCTATGGGAAGGAGACAGGAT[T/C]GGCTCAGAGAACATGGGGGAAGAGAAAGAGCTTATGGTAGGACTGAACTGTAAGACCCGTC	T/C	5	72889821	0.756
5074405238	TCTAGCCAATTTATGTGGACTTTATCCCAGTAAGATAGACTTCATGCCTTCATATGTGTA[T/G]CAGCTAGAACAAAGATCTCGATACCGTTCCAATTAATATCCATCAGTAAAACAGCTGTG	T/G	5	74405238	0.918
5076011393	GTTCTAATTTACCTGCTTCTCTTGAGCAGGGAGAGGCTCTAACAAGCAGAAGGAGCGATG[A/G]ATCTGGAGGATGGAAGGACTGGGGACAGTGAGGTTTTTTCACTCTCTCACTGCTGATGA	A/G	5	76011393	0.88
5077777345	CCAAGAATGGATAAGCAAGCCAGTGGGACACCATGAACAACAACCCAGACATCTGTGATCC[T/C]GTCAATGGATTGTGCTGAGCAGTTAGACATGGAAAAAATGAACACTAAGCATGAGCACAGC	T/C	5	77777345	0.923
5079291757	CTCCTGGTTGGCAGTTTTGCACATTCATACAAGACAAAATCTCTAATGAATTTGCATTCA[A/G]GATGGATGCAGGGTCTGTTGAGACTTGTCTTCCCTTCTCCAGGGAGGACGGACGCTGAT	A/G	5	79291757	0.993
5080882050	GTATGAACCTCCTCTTTGAGGACAAGAATTGATTTGTAATGAAGTTTTCTTGAACATTATC[A/G]TTGGTCTTCCTTCTAAACATGTCTGTAGACTGCTTTACTGATAACATATTACATGAATATA	A/G	5	80882050	0.829
5082405031	ATATTTTTACCATTATGCAAACTGAATATTTTTTCACATCCATTTATACTTTTCTTTAGC[A/C]TCAGGGCAAGCGAACAATATGCTACTCTTGTTTCTGAGCTTGGGACACTACTGTAGCTT	A/C	5	82405031	0.92
5084309584	ATATCCTATTGATGGACGGGATGGTTTTGTACAGTTCACTCAGACAAGGAAAAAAATTAT[A/G]TTTCTGAAGTATATACAAGACCCGCTCTCATGACTTATTTATCCACTTCAGTCACAAAA	A/G	5	84309584	0.875
5085968698	TTAAAGGTGAAGACACCATTCCAGGATCACCTTACAGGTGAGATTAAGTTTCAGGATAGTG[A/G]AAGTAGAAATCATGTGACCCTTCCAAAGCCCTGTCTTAGTATTTATGCATTTCCTCAACCA	A/G	5	85968698	0.923
5087557596	TTTCTCATCTGGACAAAAAGGGACTTGCTTGGGTCACTAGTTTCAAAGCATATCTCTTGGA[T/C]CCCTAGTGTTTCTCAAGGGGTCTCAGGGCAGGAAAGGAAGCCTATTGGACTCTCAAGTCAT	T/C	5	87557596	0.979
5089137795	CCATTTTTTTCCTGCTGCAAAATCCCTGAAACTGGGTAACTTATAAATATAAGAAATTAA[A/G]TTTCATAGTTGTAAAGCTTAAGGGGCCCATATCTGGTAAAGGCTTTCTTGCACTTTTAT	A/G	5	89137795	0.691
5090734317	TGGTGCTATTGATTTTTCTGAATTATGCTTTGATATTAACATAACCATTCTACCTTTATTT[A/G]AATAATGCTCAAATGCTAAGATAGCTTATAAGTTGAAATTTTCCATCATGGAGAGATATTA	A/G	5	90734317	0.652
5095772373	GAAACTGTAAATAAAACACATGGCGTTGACCATCTTTTATTCATAATTTAAGATTATTTTA[A/G]TTTCTTGTGTGTTCTATATTTTCAAAGCATTGCTTTTGCATTGGTTCCTATTCTGTTTTCT	A/G	5	95772373	0.562
5097348972	GTGGGGGTAAAATCACCGAATAGGTAGAATTTTAATGATTAACACAAAGTGGTGCAGAGTG[A/G]CAGGGAGATTAAGGCTCATATTTGGGTGCATGAATGATTCAGAGTTATACATCACTTGGGC	A/G	5	97348972	0.935
5099195367	CAGGTCTCTGCGACTTTCTAGAGTTTCCCCCTGTCCCCTAACTCCCACTGCTACATATTTC[A/G]TTCATTCTTCTGGCCCTATTGGTGTCCCTCCAGTCTTTTCCCATACATAATCCTGCCCTTC	A/G	5	99195367	0.651
5106168007	TTTTAAAGGGGGCAAAAGAAGACGACTTTAGGCACCTACACAACTTCAACAATTCACGTGC[A/G]CAGAACGTTTTGAGCAGCTCCCACACACAGCAGAAAACCCACGACTCGGACTCGCAGGCCC	A/G	5	106168007	0.954
5107857072	GTGGCCTTGTAACCAGACAGCATGTAGTTGAGAGCAATGGTATTAGCTGTAACTCAGAAAT[T/C]TTGGCAAAAAGCTCAGTGTAGGCATCTGGCAAATGCCTCTCTCTTCTTCTTCTTTGTGAGA	T/C	5	107857072	0.974
5109413667	CCCTATATATCCTTACAAAGGAATAGGTCAGGTTGTGGTTTCTGGCTTCTTCAGTGGCTG[T/C]AACAGTTTTTGGAATCAGTGTTCTAACCTTTGGAATACCCCTCCTATGTGGATTTCCAC	T/C	5	109413667	0.986

5111029312	GATTAGGGGCCTCCAACTTGGTTTCTGGCAATAGTACAGATCATGATCCTGATCTAAAGC[A/C]GGACCACAGATCTAGACAAAGCCCTCAGATGCTGCCTGGACCACAGACATCAACATGGC	A/C	5	111029312	0.988
5114178292	TCAAGGTTTTTGTGACTAGGTCAATGTCCAGATTTCTCTTTGCTTACCCTGCAGAGTACC[T/C]TCCTGTACCAGAGAATATAGTGTAGGAGTGAAGGATCCATGTAGATACCAGTTGACTTC	T/C	5	114178292	0.942
5115760857	ACTTGTTTAAGAAGAGTGCTTCATTCATTTTTCAAAGAAACACTAGGGCATATGTTTATTA[C/G]GTCCAGATACAGAGTGAGATTGGGGGAATGTGTCCCGAGTTATAAAGTGATGTTACTTAGC	C/G	5	115760857	0.965
5117431202	TAGTGAGCTAACAGGAATGAATACCTTCATGTAGTTGACTGCACAGACATCAGTTAAATCT[T/C]TTGTTTTTGCTGTATCTTTCTTGGTCATCTGTGATCAATATTAGATATTTTAATCTTAAGT	T/C	5	117431202	0.912
5118962726	CAGTGGGACTAAGAATTAAATATAAGATACTAAAGGTAGTATGAGAAAAATCAGTGCTTTG[T/C]GTGAGACCACATTAAGTAGAATGGTTCAGAGACTAAGATAGGATCCTCGGAACCCTGTGCT	T/C	5	118962726	0.838
5122070525	AGCCGGGAATAAAGGTGTGAATCACCACAGACTAGCAATACAGTGTAGTTTATGAAACTT[T/C]GAACTTTCTAGAGTTCAAATAATGGGAAACAATCATGGAATTGTGGTTCAAATGGTTAG	T/C	5	122070525	0.739
5123700718	TTCTGTTTTGTCTGTGTGGATCTTTGTTAGCATCAATCTCCTCTGGCTCTCTTGTCAGTC[T/C]CCTCTTCTACATAGTTTTCTGAGCACCCAGAGGAAGAGTTTGATGAAGAAATTCCATTT	T/C	5	123700718	0.971
5127022967	CCAAGATATATCAAGCTGAAGAGATTACTATGGAGACAATGAAATCAAAGCTATGGTCAG[A/G]GGACCACATGGGTTAGAAGAGAAGACCTTGAAGTAAGAATGGGGGAAGGGATCTCTCAT	A/G	5	127022967	0.95
5130181490	GGCCCTGGAGCACCGATTTCCCTACCCTAACAATTAGAAGATGTTTAAGGCAGTTTGGTTT[A/G]TAGAAATCTTAACGTGGACGGTACCCACTGAATAACTACAATGAATCTAATAGAAAATATC	A/G	5	130181490	0.922
5132074162	GGGGAGCATACAGCGTTGCCTCCCGCCCCCACCCCCTCCAAACCTTTCAACTTTTGGTAT[A/G]CTTAAGCATCTAAACATATTTGTTCACTTAAAACAAACTGAATTCTGAGTCAAATAGGC	A/G	5	132074162	0.911
5136722385	AAATACAGTAAGGGAAGTGAATATAGTAAAAGACCAGTTACTAGGCAAAGGACAGCACTCT[A/G]ATGTATGAGAACAGATTCAACTTGATTATCTCACTATAGGACAAAGTCACTTAGTGGCTTT	A/G	5	136722385	0.907
5138298219	GACCTGTTTCCCCTGAGAATTCATTCTGATTGGAAAAAACCAGAAACTCTAGTCCCTGTC[A/T]CCCAGAGACAACTTTTCACCATGGAGTGTCTCCCTCGAGCCTCTCTTCAAGACCTACTG	A/T	5	138298219	0.969
5141430321	CCTGGGCTCAGCACGTAGTCCAGGTGTGGCTCTCTGTGTTAACCACCGTTTACTGTAAGA[T/C]GCTTCTCTGCTGAGTTGAGCACTGACCTGAGTCCCAGCAAAGTGCATAGAACTAATTTC	T/C	5	141430321	0.889
5142998347	ACCCACAACACGCAAACCGTTATTGCCCTGGTCCAGACTTCCAACACTGATGTCCTAGAC[T/C]GAGTCCCCTCCTCAGTGAGGCTGCAGTCTAGGGTTGGCTGGTTTTATGTGCTTACTCCA	T/C	5	142998347	0.971
5147654034	TGGCAGCAGTTGCCCTGCAAGAAATAAAAAGCAACCACACAGGAGATGAGCCAGAGAGTC[T/C]GAGGCTGACAGGGAACACGTGACGGAGACAGAGTTCTGAAAGAAAAGTAGCCAAGCGTG	T/C	5	147654034	0.903
5150773470	GCCGACCAGGGCAGAAAGTGTGCAGGGACCCACACAGTACAGAATGGAGCGTAGGTCTGTC[C/G]TTAGGAGTGATTTTGCACCCCTAAACCGAGAGGACCTCGGGGTGCGAGTGCTGCAGCGCTC	C/G	5	150773470	0.879
5154056634	TCCCTCTCTTCATCTCTTGTAGCCCAGGCTGGCCTCAATTTTGCTACATAGCTGAGAACTT[T/C]AACCTTCTGATCCTAGATGATCAGAGGATCATGTGTGCCACCATCATACCTTTCTTTCTTT	T/C	5	154056634	0.748
5155616304	CAGGACCCAAATAGTGGAAGGAGAGACCAACTTCAGTAAAGTTAACCTCTGACCTCCAAA[A/C]ATGCTCCAAGGCATACCTGCATGGCTGTGTACGCGCACGTGTGTGCGTGACACAATGGT	A/C	5	155616304	0.885
5157190339	TCCGTCCACCAAATTACAGTCCAGTTCAAAGCAAAATTTCTGTTTGCAAAGCAAAGAACAG[A/T]GCTGTGTCAACAGGGGCTGGGATTCGGTTACAGGCTCATCTCAGGAATGCAGACCCCTGCA	A/T	5	157190339	0.837
5160400750	CAACAAAAACCAAAAAAAAAAAAAAAAAAACAACCCTCTAGCTTTAGTTTCTGACACTGGC[T/G]GCGTAAAATAAGAATTCTCTAAACCTTTACTTGAAACCTTCCTGAGTGCTCCAAAATACAC	T/G	5	160400750	0.86
5163432237	AGTCTAACTGGAAAAAAATGTGGAAACATTTAGTTAGAACAGTAGAGCAAGGTATTAAAGA[T/C]CCCTTTAACGGGTTAACTCTGAGGGGAATATGCAAATAATTAGGTCTTTAAACATGCTTTT	T/C	5	163432237	0.626
5165261734	TATCTCTGAATTATTTAAACTACAGGTCCCCATAACTCTTCCCCTAGATTGATGTCAGTTC[A/C]TACTAACCTAACATCCTGTCTCATCCGCCCCAGAACCTACTCTATACATGGAAGCTTCCCC	A/C	5	165261734	0.921
5167650243	GTTGCCTCCCCGTCCTGTTTTCAGAGAGAACCCAGGTAGGAGGCTGTTACCATGGTTTAC[T/G]AGGGAGCCAATCAGGATAAGCAGATTTTGTGCAGTGGAATTCTTACTCAAACAGGGAAT	T/G	5	167650243	0.91
5169200076	CTTGCTCTGGGGGAGGGCCCGAGTCCCAGTGCTCACACCCACAGAGCAGCCCACGATTGTC[A/G]CAAGCCCAGCTCCAGAGGACTTGGCAGGCATGTGGGACACATAGGTGAGTGTAAACAGAAC	A/G	5	169200076	0.674
5171011819	CCCTTCACAGACATGCCCACAGGCCGGTCAACTCTGACGGTCCTTCCTCGGGACTCTTCTC[A/G]GATAATTCTGGGTTGAGTCATATTGACAATTAAAGATAACTGTCCCAAGCCTCTTCCTGGC	A/G	5	171011819	0.985
5172536318	CAAGCGCTGGGATTTTGTTGTTTTTTTAATACTTAATTTTATTTTGAAGTGTTGAAGGACC[T/C]TAGAGACTTTTGCCAACCTTACACCCACCGCCTAAGGTTAAGATTAAGCCGAGTACCAGCT	T/C	5	172536318	0.727
6000013290	TTTCAGGGTCAAGTATGGAGTATTTTCATAACTCTGAAGCACAAACTGGGAAGTCTTAAT[A/C]AAAAAACAGCACAATACATCATTCAACTGCTTAGTCTATGTGCTCTGTTCTTCACTGTC	A/C	6	13290	0.936
6001561442	TTCTTATGGTACTTCCCTGATCTAGCACATTCTCTTTCTCTGAGGGACTACGTCTAAGTCC[T/C]TACCCGAAGCCCAGGGTCCTGCCTTCATCTGAACTGAGCCTGCAGTGTGCTGCTCTGCTCA	T/C	6	1561442	0.95
6008158299	AGGGAGAGGTGGGTGTTATCACCCACAGACCAGAGAAGATGCATATTGCCCCCTATGGTT[A/G]TCCATTACTATAATAATCATCAAAAACTACTGATAACTCAGAACACGTGCACTGATTCA	A/G	6	8158299	0.853
6009743133	TAATTAACTTCAGGATGGACTCTCAGACCCTGGGGTAGCCTAATGGGCCAGGGCACTTTTC[T/G]GTTGGAGTGAATAACTTATCTTGGGCTTCTTGTATCATTGGTGTCATTGGTGTCTTTCCTA	T/G	6	9743133	0.993
6011372152	CACCTTCATTTTCATCTTTCTGTCTCTTATTCTGATCTGCAATTTCTTGTATCTTCAGTTC[T/C]AGTGTTTCCTCTAGGCCACGTTGCCAAGTCTTAGGTTTCTCTTTCTGTTGTTCATGCTGCT	T/C	6	11372152	0.538
6012883623	ACACTGGAGGACACTGGTAGGGAGGAGAGTGTGGATTTGAGCCAAGAGAGGGCACAGGAC[A/C]GTGAGCAGTGATCCCTTTCAATCATATCATGTGGGAGGAGTTCTTTTCCTACATCTAGC	A/C	6	12883623	0.829
6014629683	TTCCCTCTGAATCACTTGGGCAAAAGGCACAGTCCCCTACCCCTCTACATAAAAGTAACC[A/G]GTGCCTTTAGAGCTCTTAAAAGGGAAGGAATCCGGCTTTATTTTATTGGACCAAACTTG	A/G	6	14629683	0.921
6016421220	CATTTCTAAAAAACCAACATCCTGTTAACATCAGCTGACCACACGCATATAGTGTCCTTG[T/G]CCTGTGTTAATGTTTCACAATCCCCCCTCCCTCTGTTACGCATGTTATGTTATAAATTC	T/G	6	16421220	0.706
6018033419	ACCCTCTGCCTCTCAGTGTTTTCTACCATCACTTCTGCAATGTTCCTTGGGTCTCATTGG[T/C]GGTGAGGAGATTAATGTAGGTGTCCTGTTGGGGCTGAGTACCCATGTCACGTTTGGGTT	T/C	6	18033419	0.953
6021142260	TGCCAGGACTGCCACTATGTAGGACTACAGTTGCTTCTTCAACTGTTTAAGCCTCTTCATC[A/G]GTCAGGCTTTCCGTCGTCAGCACCACTGCCATCTGTTGTTCCTGCTCACTGCTATTCCTGA	A/G	6	21142260	0.925
6025496450	GGGGCCAGGCAGAGTCTGTGTGGTATAACCAGGCTGCAGGAGGGAGTGGATGGAAGCCTC[A/G]GGGCAGTGCTGGGTTTCAGTGCTGAGGCAGGGGCCCTGGTTGGGGAGCCCAGTGCCTGG	A/G	6	25496450	0.645
6032978412	GTGCTCTTTAGCACTTGTGTAGTGTGCAGACAATTTTTGTTTTAATGATTTTGCTTTGTAG[A/G]CCTTCAGCTACTCTGTAGGTAGACTGCCTCTGGGCATAGAAAAGAATAAAAAAGAGCACAG	A/G	6	32978412	0.878
6036190841	GGGCCAGGTACAAAATTACTGTTTATCATTATTTCTTGTTCCACTTTGAGACCCGTTGCC[A/G]GGGAAACACCTGTTAGAGAAGCCTGCCCTGTACCCTCTCTGCTTCTCACAGAGTTTGTG	A/G	6	36190841	0.718
6037905008	TATAAAATCCTTACTTTATCTTGGATTGCAACATAGTTCTGCAATCATATGTGTGTTCTGT[T/C]TGTGGCTACTATGGAGAAGGGCACCTGAAAGACACCAACATACTTCTAAGAACCGTAAAGA	T/C	6	37905008	0.878
6039447989	TCAGCTACTCGGCTTCCTTACTATGAGCTACAGGTGGGCTGCAACACCCTCCAGGCTGTA[A/T]CATGGGCTCTGGGAATCCACATTCCATCCTCGCATTTAGCAGGGCAAGTGTTTTACATG	A/T	6	39447989	0.656
6041140217	TTTATTAGCAATTCCCCAGGAGGAGGAGAAAGCGGTAGAAAAGTTCTCATAACATAAACC[A/G]TACAATTATTGAAGGATTCCTTTACCTGTCTGAAGACAGTGATAAAATTGCATTATGAA	A/G	6	41140217	0.811
6044507052	TTGAAGGGAGAACAAGAGGGAGAAAGGATGAAGAGACCTGAGTCACCAGGCTAAGCAACTC[T/C]CCACTTCTGTTCCCTTCTCTCAGGAGTCAAGGCTTCTCACTATTTCTGGGTTGCATTTGGA	T/C	6	44507052	0.924
6046019027	AAGCCTACAAAAAAAAATCTCAGACCAAAACCCCAACTGCGAATGAGCCATATCTGAAGGG[A/G]AAACTCACAAAGAAAATACAAAATGTGAAGAATCACAAGGGGACATGTTTTAGGTCATTTT	A/G	6	46019027	0.973
6049571373	TTCCAGGTCTGTGACAAAGGAGCTTCAGAGCCAATTCAGAAATATTTTTGGGGACCAGTTA[A/T]TTCACCTTAACCCAAACTGTAGATCATGAGTGAGGAAGCTCTTGGATTTAGGTAGACTGGT	A/T	6	49571373	0.915
6051304734	TGCCTTTTACTCTTGAAATATTTAACATTTAGTCAATGAAGTTGCTTCTCCTAAGTTTAA[T/C]ATTGATATTGTGAAAATAAATGCAGTTCAGATTTCTTATTTCTTAAGATTCATTCTTCC	T/C	6	51304734	0.83
6052974072	TTCTATATACCACTAGTGTTTTTAGGGGAGAGGTTTTTAAATCCATCCAATTCATAATAG[T/C]CACACATACGAAGGCCCAGAACGAGGAATGAAGTCAAATATGTAAGGGGAGGACTTCTG	T/C	6	52974072	0.98
6054555767	AATTGAACTTGGTGTATTTTGAGTGGGGGAAGCAAAGGTGGGGGTGGAACTGGAAGGAAT[A/G]GGAAGTCAGTGAGATCATGGTGCATTAGAAGATTCCTAAATAATTAATAAAAATATTAT	A/G	6	54555767	0.857

6060907377	TAGCAACCAGAGCTATATATGTGACAGTCACGCTGAGCAAGTGAATAACAACTGTTTTCAG[A/G]GGTGACTAGTTGTAATTGATTCTGTAAAACTCCAAACCCCATGCATTCTTTTCTCAATACA	A/G	6	60907377	0.789
6064062891	ACCACAGTAATCTTAGGTTCTATTAGCTATGCTTTCAGTGATAAATGTTGGAAGAAATTC[A/C]GGGTCCAAATGAAACTGGTAACTATTAGATTTGTAAGGTTCACTTTTCTATTGTCCAGT	A/C	6	64062891	0.885
6067096063	TCTTAGGTTAATATGTCTGCATGTTTCACATGGATGTGGAGATTGTTTGAAGTGATATTT[A/G]TTAATTCTTGGCTGTTTTTACAACTTATTTACTTCAACTTTTGGATACTACTTGGTCAC	A/G	6	67096063	0.738
6068704582	GAAGTTTGGACAGGTACTATGTTATTGATTCTCTACCTGTTTAAATTTTAAAAGTTGATGA[A/G]ATATTAGCATCACATAGCAAGATAGATCTGATTGTGTGTGGGGGGGCAGGGTACATGGTGT	A/G	6	68704582	0.666
6070332650	ATCAGCCCTTTCCTGTATATCGGCTGAATGTTAGTCCATATGAATTTTGACTCAGTGTTTC[T/C]AAATAGTCACATAAGATTATAGTAGGAAGAGACAGTGAGGTTTTTTTTCATAATTTTCAGA	T/C	6	70332650	0.638
6073669372	CTTCATTTGATGTTTTGAACAAAAAGGAAAGAAGTATAGCGTGAGGGCTGACATTTCACAT[A/C]ATGGATGTTGTTTAACAAGTTTTCACAAGCTGGCCCTGACTGTCAGAAATGAAATGAAACT	A/C	6	73669372	0.899
6076890237	GAAAAAAGTCTGTCATAGCATGTCCACGATCATTATTGATCTTCAGTGTTACACACATAC[A/G]TACTTCTAGGTAGTGGGCAGTGGGCAGGAGATCTGTCTGCATCAACGGAACTACAAGCA	A/G	6	76890237	0.627
6078607389	CAGTGGATATTTGGGTCTGTTGAAATTACCCAGGAGCAGAAAAGGAAAGGTCAAATATAAT[A/C]TATCATACATAACAATATTCTCGAATATGTTTCAGTGAGGATATTTTTCTCTGTCGTCACC	A/C	6	78607389	0.755
6080367921	TTAGAGTTGTTGGCTTTGTTCTGAGGCAGTCAGCTAGCTACTAACTGAACCTGATTTAGC[T/C]GCCATTCTGTCTCCCCATGTGGCTCTCACTGCCTGCTTCTGGTTCCATCCATCTTCTTC	T/C	6	80367921	0.789
6085254123	TTAAAACATATAATCATATGAAAATATAATTCATAGTACTTAAAAGTCAGAGGTGATATTT[T/C]CTTTCAATGGGTACGCAGCTAACTTTCAACACACAAACACACACACACACACACACACA	T/C	6	85254123	0.853
6086850472	AATTCTAAATAATCTGTTTTTGACACACATTGTTCTTTAGTCCCACTCTGCGATGACTGG[C/G]TAATTTTCTCCCCTCCCCCATAGCTCACTTGGATGAAGTGGCACAGAAAACTATTTCAG	C/G	6	86850472	0.698
6088424825	CATCTTGGATAGTATTTTTAAGTCTCCGAAACAAGATTGGCTCTCTTCTAAGGCTCACTAC[A/T]TTAAACTTTATTTTATCCTTTAAATTCAGACCAAACCTCATTTTTCTTTTGTTTACACTAT	A/T	6	88424825	0.773
6091698875	CATGCTGCGGTGATTTAACAGAGATGAGGCAAATCAAGGCGTTAAGTTACCACTAAAATA[A/G]TTTAAAATAATACCCGTAGTATCCAGTTCACTGAATAAAATTAAAAGGATAGAAACTTG	A/G	6	91698875	0.796
6093557664	GGAGGCATCAAGTCTGGGACCTCTGCAGGGGCCCATGCCCCACCCCCACCCCCTGTTTAG[A/C]TTGAGTCGGAGACGAGGGTACTCAGGCTCGAAGATGGGAGCTGGTTCATCTTAGCTGGG	A/C	6	93557664	0.668
6095106508	TTAATCTATTTCTAACCTTAACTGTTTGCCCTTGAACAAATGGTGGTAAAATGTGCTTTT[A/T]ACCTTAAAATCCCAGCAATTATCTTTGCTTCATCATACCCATGTTATGTCAAGAAATGG	A/T	6	95106508	0.903
6096659693	CACATTTAACATTCTCTTCTAAAACCACTTGAACCAGGTCCCCAGAGGTCAAATGACACTC[A/C]GCACCATGGTCTTCCATGTTCCTGCTAGAATGGCCTATTAAGCTCTAATTAAAGGATTCAA	A/C	6	96659693	0.789
6099761123	CTCGGGGAGAAGGGCAGGCAGCCCCAGTTATGTGCCAGCAAAGTGCTATAGCACCACAGC[T/C]TAACCCCCGGCACCACAAGAAGTTGCGCCGGGGGCTCAAAGGTAGTGTGTAGTGCTTAT	T/C	6	99761123	0.766
6101338779	TCAGATGCTCCTAAGAGCAACCTGACTGAAGACTACATGGCAAGGAATTTCTAGAAAACTT[T/C]CCTTAGCCTGTAATTCAAAGACGAGTGTTGTAATTAGACCCACTCATCTTGTGTGAGGTGA	T/C	6	101338779	0.905
6102864807	CCATTAACCATCTACAACCACATTTAAGTCAAGATGAAATAATATGTAAAAATGTACTGC[T/C]GAGGCTCGCTCACCACGTGTCAAACACCTGATGGCGTAAGGGCTGGTGGCCCCTTCATT	T/C	6	102864807	0.785
6107754298	CAAGCACGTCATCATGCTCTTTGTTCCTGTGACCCTCTGCATGGTCGTTGTGGTGGCCAC[T/C]ATCAAGTCAGTCAGCTTCTACACCCGGAAGGATGGGCAGCTGTATGTATAAGTGTTTCA	T/C	6	107754298	0.951
6109613591	TAGAAAGTTTCATCCAAAGGAGAGAAAGTGGGAGAAGGATGAATCGGGAAGTGGAGGCGGA[T/G]ACAGGGGCTGGTGTCAAGCAGGGAAAAAGATGAAAAGTGCTAAGCAGAACAAATGATCATA	T/G	6	109613591	0.625
6114412200	TAGCTTAGGTGGGCTCAACTCAGATTTGAATAATGTTCTCTGTTCAAATTTAAAGTCTTAG[T/C]TGATCATCTCTGAAAGAGCATAACCTTTTGGCTATATGGCACCCTGGAAGACCAGACTCCT	T/C	6	114412200	0.842
6115919556	CTGAATCTACTTAAAATTTTACCCATGGGATGCGCACATCATTGCACATATTAGCTTCTC[T/G]ATCCAGCCATTTAAAAGCCTGTATGTTTCTCCCTTTATTCCTGTTATTAGTACTGTTGA	T/G	6	115919556	0.957
6120589080	TCAAAAGTATACGCGAATGGGTCCATGGCTCCTGGTATTTGTAGCAGAGAATTGCCTTAT[T/C]TAGCATCAATAGGAGGGGAGGGCTTGTTGCTCCAGCGAAGGAGGATGCTAGAGAGAGGA	T/C	6	120589080	0.679
6126772459	TGGAAACCTGTTCTTACTGCTTTTACACGAGAAGCCACACACGTGTCTGCTGTAAGTTAAC[A/G]GCACTGTGACCTTAAGATTCCACAGGGCTGGGGGGTGGCAATGTCAGAGACCCAACTCCTC	A/G	6	126772459	0.823
6130237987	TGAGAAACTGGAATCAGCTTCTCTTTCCTTCTCTGACACAGCATAGCAGTTCTTTTTGCCC[A/G]TCTAACTCTATTCATGACACTGATCTATGTGCTTAATAACTCACCTCCCTCATTGTGACTT	A/G	6	130237987	0.932
6131878479	GGACTGAAAACTTTAATTGCATTAAAACTAAGCATTTCAGAGTGGGGAGAGGGTGCTGGAG[T/G]GATTCTCAACCTTCCTAGTACCATGACTCTTTAATACAGTTCCTCCTGTTATAGTGACCCC	T/G	6	131878479	0.553
6133578383	CCTGAAGTCACTTAGAGCAGGTATGAAGTTCCCATAGCATTGTGGTAGACTGAGGCTGTCC[A/G]GGTTGGGCACTATACCAATTAAAGGAACTAGTAGGGAACACTGTGAATGGTCCTGGTACAG	A/G	6	133578383	0.879
6136673954	GTACTAGCGCTGATCCAGGGGGTCAGATCTACTGCCGAGCACTAGCCACACAGAATGTTT[T/C]GGCCTGGGGTAAGAGGTTTCCTTTTCTTTTCAAACAGCCATCTGTGGTAGTATATCCTT	T/C	6	136673954	0.914
6138214721	ACCAGCAGGGTAAAAAAAAAGGCTAAACAAAGCATTATGAGATAATAAATCTCCCCAAATA[A/T]CCCTGGGACACGGGGTCTGCCCTTAAGTGTGCCTTGAATATTCAGTGAGAATCCTTTGCAG	A/T	6	138214721	0.931
6141283496	AATGATGACAATGAAAGATGTCTTCAACACTGTGTAAAAGGATGGCATTGTCTGGCAATAA[A/G]AGGAGAGGCTCTTGCACCTGTGATGCCTTGATTCTCTACTGTAGGGGAATGCCAGGATGGT	A/G	6	141283496	0.74
6142802595	AGTCCCCTGCTGGCTCAGCCCCAGCAAACAAAGCATTAGGCAGCAGCTAAAGGGAGCAGAG[A/C]CTACAACTGGGGCAGTAGAAGCTGTTCCCACTCCCTGCCTGAGTTCTCTCCCCTTGCCCTA	A/C	6	142802595	0.656
6144550712	CCTAGGGATATTTGGATTTTTTTTAGCGCAATTTGAATTTTTCCTGAGTTCTTTCTTGG[T/C]AAATTCATTATTACTATATAGGAAAGTTACTGGGATTTGTATATTAATTTTGAAATCTGCT	T/C	6	144550712	0.614
6146192021	AACTGCTGAGCCATCTCTCACATTTTTAAACATATTCTTACTGTGCAGCCCAGATTGACTT[C/G]GGACTTCTAACCCTCCAAAATGATTGCTTTCAACAGGTGTTTTTATCCTTTAAAAATGTCA	C/G	6	146192021	0.956
7001590648	CAGAAATGACAGCTGAGTTTGAGGAACCTAAGACATCTTGCAGCCTGGATCAATTCCAAAG[T/C]AGTATAGCACACAGCCAATAACCCAGAATCTGTTAGACATGGTGACACACTCTCACAGGAG	T/C	7	1590648	0.984
7003226327	CACTGTGCTTCTGGGAATTTTCCACCTGGTGGACTTGGTGTGTGAACCTCAGTAAATCTT[T/C]GGCAATATTGTAGCACAAATTACTCAAGTCTCTATTTTCTTGTTATATCCCCCAAGTCT	T/C	7	3226327	0.812
7004873961	TTGGGTTAATACCTATGAATGGGTCTTGTGCTAGTTTTAATTTTAGCTGTTTAGAAAGGTC[T/C]TTATTGATTCTGGTAATAAATGTAACATGTTGTACTCTCACCAGGAGCAAATAAAGTTTTC	T/C	7	4873961	0.543
7008005145	AGTTTCTACCCTCTCTGAAAAGAAGGAGATAGGGAATGAAGGGAAGGACTGTGTGAGACA[A/G]AGGCCAGGAAGGGTGCAGTGAACAGGATGGTCGCTTGATTTTTGACAAAGGAGTAAAAA	A/G	7	8005145	0.667
7009511628	ACGGAAGTTTGCAAAGGCTTTACTGCATTGACTGCATTTATAGGGCTTCTCTTCCGTATT[C/G]ATTCTTTCATATGTCTGAAGAAAACTCTTATGTGCAAAGGTACATTGCTTCTTGCCATG	C/G	7	9511628	0.754
7012670072	GGTAGGGAAGGAATAATTGGAATCCTCTAGGGACAGCAGTCACAAATGCATCCTAACTCC[A/T]TCAATATTCCTAATACTAGACAAGGAGTGTCACCTCAGCTGCTCAAGGCTGCTTTCCAA	A/T	7	12670072	0.887
7024166242	CAAAGACCAATGGGCTTCACAGAAGGTAACAGGGCAGCAACATTAAGAGTAGATTTAGAAG[A/G]TGTTGAGCCATAAGTACTGGAGGGAAGGGCACGCTAGCAGTAGGAGGCTTGGAAGTGCCCA	A/G	7	24166242	0.915
7031949612	TATTATTCTTCCTCCTCTTCCCTTTTTCCTTGTTGGCTTATTTTCTGTCGTTTTCTCTGT[T/C]AGGCAAGCACTGTATGTTCAAATGTAAGTGTAAAAAGGGCAGTTACTTGCCAGTTCTAG	T/C	7	31949612	0.956
7033543143	TCCACTAAGCTTCCCTCTTCCCAGATGATCCTAGTTTGGGTCAAGTCGACAAAACCCTAA[C/G]CAGCACAAACGTCATTAGCCATCAGAAAAGTTTCAAATTAAAACCGTTCTGAGATCCCA	C/G	7	33543143	0.815
7035148996	AAATACTTAATTATCTGCTTCTAATACAAAATATAATCCCAACTCATAAAATTAACTTCA[A/T]GCCTACAAATATTTACAGTGTGATTGAAGCATGCATGATTGGATATCAAGTCACAGCAA	A/T	7	35148996	0.786
7038377962	CTCAGTCTCTATAATGTTATTTTTGAGCTTCTTTAGCAGCTGGATTAAGTTAGGCTTCTAC[A/G]CGTCCTTTCTTTTCTTTTTTCTCATTTTACAGTATCAGAATGAGTTATGCAAAATCTTATC	A/G	7	38377962	0.669
7043152083	GTTGAGTTTTGTGCAGGGTGACAAGTACTACTCTATTTTCTTTCTACTATACGCAGATATC[C/G]AGTTTGACCAGCAGTACTTTTCTTATTTATGTTTGTTTTAGCTATCCTGGATTTTGTGTGC	C/G	7	43152083	0.705
7044716533	ACAGCGCACAACTACATATTATTATTAGAAACTATTATTCTTACTTTTGTTCACATACAT[A/G]CATGAACTATGGCAAGTTCAAGTGAAATGAGAAGTGGGAAATGAATTTAATCCTGCCTG	A/G	7	44716533	0.854
7046228404	ACTTGTCCAAAGTTGTTTTATCTAAATTAATCAGTGATGTAAAAATTAAACCAAATGAAT[A/G]GTGCTTTTGAGTTTGGGGATCTGTGGACTATCTAGGTAGTAGACTCAGGAAGGCAGCTC	A/G	7	46228404	0.974

7047763375	CTTATTGAGCTTGCCTTTAATTGAAACTTTAACAATCTGTCTAAACATATGTATTTTCAT[A/G]TTTAGAATCAGTGGCTATTAACATACACATAATATTAAGGCTTAAGCATCTTATATGGT	A/G	7	47763375	0.659
7050867822	GGAAAGACAGAACATTTCCTTGTGGATATTTCTTTAAATGGCTACTGTTCTGTTTTTTTTT[A/T]AAAATAGATTCCAGTGGAAATTTCTCTTATTTGAAGTTTTCTCAGCTCAGAGGACTTCCTA	A/T	7	50867822	0.816
7052808733	ACATGCAACTGCCAACTGAAACACAAGGTCTCAATTTGTTCAATAGGCCTTCTCATGAAGT[A/C]AGGCTTCAGCTACGGATTAACTCCATAAGCCTGTGTAGCTTCAACTCATTGTATTATTATT	A/C	7	52808733	0.917
7054565301	TTTGTCTGCGGTGTCCACAGATGGGATAAATGGCAGTGCTGAGACAAGCAGGGATAGCTA[A/C]CTCCAAGGGACACTAAAACTAATGCAGATGGCTAAGGGAAACATAGATTGTCTTGAAAA	A/C	7	54565301	0.967
7061140277	TTACTCAGAACACATAACTCCAGGGTTATGTTTGTTCTCTTGCAGCCAATCACAGCACAGT[T/C]CAAAAGACCTTGATCTCTAAGAACATTGATCATCGTGATATCCCAGTAGCTAAAACAATAC	T/C	7	61140277	0.988
7064576568	ACTCAGATTCTCTCCTTAGCTTCCTAGCCTCTAAAAATTGACTTTGAAAATTGAAGCAAAA[A/C]GTTCAGATGTGGGTGTTATATCAGAGGGTTCTGATGCGTCTCAGTTCTGATTGGCTGTCTA	A/C	7	64576568	0.787
7066130885	AGTAATAGTAATAGTTTTTTATTATCTAGTAATAAAACCCTGCCTAGTGCATGGCCTCTG[T/C]GCTGATCCAGTTACCTCATCTTGGGAGATCTCCCACACCAAATACTCAAGAGTTCTGGA	T/C	7	66130885	0.965
7067766886	CTGGTTGGTGAGGTTTAGATTGCAGGGGTAAGGGGAATGTCCTGAGAAGGGTGTGCATAGG[T/C]CAGCAGGAATTTCCTTGCCCGTCTCAGCCCTTCTAGCCACTGAGGTGGGAATAACTGCTGT	T/C	7	67766886	0.837
7075273694	TGCAGTTAAAGGACTCAGCAGCCTGTATTTTATGAACAAAATGGAGGAGATTTCAGTTTGC[T/C]CTGAGGGTTACAGACAGAAGAACTACTGCTTACTCTCTGTTCTGTTGTTGCAAAGAGATGT	T/C	7	75273694	0.955
7078441808	TAGAATTCTATGTGAAGTGAATATGGTAGATTCCAATACTAGAAAATCATCGACCAATCAC[A/G]GAATCCCAAGTACAGACAAACATTAATGATGTGCCAACAGATATGCAAAATAAGTGATACA	A/G	7	78441808	0.83
7086661510	TCCTTAAATAAATAAGACTAGTAATAGAATATCTAACAAGAGCCCTCATACAGATATTTT[T/C]TTCAGGAAGTGTAACTTATCTTTATAGCTGAAATTAGATGAAACCCACTGATCAAATTT	T/C	7	86661510	0.621
7088227484	GTGTACTTACAATGTAATAGACTTGAAAGAGAGGTATATCTGTGCTTGGATCCAAATCTTA[A/G]ATATGATGTTGATCAGAAAAATGACTGTAGCATCAAGTAACAAACCTTATAGTTCAAGAGA	A/G	7	88227484	0.912
7091402105	CCACTGAAGTGCCAAAATGGGGAATTTTGGTAAGAGATGAAATGTAGTATCTGGTGGTTG[A/G]TAATTGATAAGGAAACTAGAATAGAGTACATTCAATTAGACAGTGACAAATGTCTCTAA	A/G	7	91402105	0.728
7092964847	CTATGCCAGCAATGCTATAGGAATCGAACCCAGGGCATTTTGCATGCCAGGAAAGCACTCT[T/G]CCACTAAGCTAATCCCCATCCCTTCCATGTATGCCTCCCAAATAGAAATATAAGATACAAA	T/G	7	92964847	0.904
7095066786	GCCAGAAGCTTTGTAAACTGCCTAGGGTTTGGCCCTAATTTGAGTTTTCCTGATTTCTCCA[A/G]TTTGTATTATTTCTTTGATTCCATGCACCTTCTGTGTTTCCAGTATGGACTATGTTTTGAA	A/G	7	95066786	0.9
7101196778	ATGGAAAGCAGCACCTTCCTTTTTTGTCCTTAAAAGTTCAACCTAAAGCTACTTCCATTT[T/C]CAGTAGGCCTTGCCCTTGAACTCCAACCACCAGAGAACTGAAATCTCTGCAATTCCCAA	T/C	7	101196778	0.993
7102913634	CACGTAAGTAATGTATATGTTTGACATGCAGGATAACTGATATATGACACCCCCAAAGGA[A/G]TAGCAAACCACAGGTTTCCTTAGATCTATCCAATCAAGAGTTAGATTAAGAAGGACAGG	A/G	7	102913634	0.844
7104438040	TGAAGAAAGACTTTCTTCCAAGCTTACTTGTGGACTCTGGGGGTTGAGAGGATCTCATTCC[T/C]GTCTCTGAAGGAGCAAGGACATCTTTAATATTTAGTTATCTGGAAGCTAAAGCTATCCCAG	T/C	7	104438040	0.875
7105956466	GCATGGCACAGACATGGAGATAGAAAGGCCAGCTCTCACGCACCCTGCAACTGTGGGCTTC[C/G]GTCTTCATAGCCCTCACGCACCCTGCAACTGTGGGCTTCCGTCTTCATTCCCTTCCTGCTA	C/G	7	105956466	0.66
7107555394	GAAAACAATGCAGGATATTGGCTATGATATTGATTGCCCTGCACAACTTGATGGTAAGGAA[A/G]TATTACAGAAGATATATTTATATATATCATCAAACATGGAGAAATTTGGCTGGTGTCCAAC	A/G	7	107555394	0.534
7110711496	ACAACTGCACAAATGAACTCATAGCAGCTGTGTTTGCTTGCACAATTTCTATCATGGAGC[A/G]GGGAAGGCTTTATGAATCCCCAACCCCACCCCACACCCCAGCCAGGAGCTACTGTCAAT	A/G	7	110711496	0.954
7115986732	CGTGTGTGTGTGTGTGTGTGTGCATGGAGATGAGTGAGTTAGTATGTGTGTGCACACACATG[T/C]GATATAAGCTAGAGGCTGCCATCAGCCACCTTCCTTCACCACTCTCTATTTTGCTGTTT	T/C	7	115986732	0.805
7117925849	ACTGCAGGTTAGCCAACTCCAAGTCCCACCCTACTCAATCTGCTGCTGTCTTCTCTAGAGG[A/G]AAAAGTACACTTCAGGCACTAAAACTATATGGCCTTTAGTAGTTTATCCTACTTCTGACGA	A/G	7	117925849	0.942
7119813204	CCCCTATGATTCCTTACATAGGCCATTTGGCCATGTGTCATCTTGGCCTCTTGGTCCACT[T/C]CTCTCTTGGTTCTCTTGCCTGTCTTCGTTAAGGTTTTACTGACACGACCCAGGCAACTC	T/C	7	119813204	0.866
7121418726	AACCGGCTTTTGTATAGGGGCCATCTCTGTGGGGATGCTGGGGAGACAGGGAGGCTGGAGA[A/C]AAGGTGCTGCCACTGAGGACTGCTTCAAAGCTGTAGACTGATGCAAGGAAGAAGGTGTTGC	A/C	7	121418726	0.708
7124933938	GCTTCGGGAGCTGTCCAGGGTAGATGCGTACCAGCAGGAAGTCTGCGAAAGAGCATAGTC[C/G]GTTCTGGAGGCTTGAGTGGATCTCTACAGCAGCTGAAGGGGCAGGCAGGACCTGGAGGA	C/G	7	124933938	0.947
7128013636	AGTGAGGTATTCATTTCTTTCATCTTGTCTTTGAAGCCTGAGATTCTTTCTTCCGTGTCTT[T/G]TACTCTCTTGGTGAGAACCACCTGAAGTTTCTGTTTGACTTCCTAAATTTTCTCTTCCAGT	T/G	7	128013636	0.775
7131041216	GAAGCATTTTTTTGTTATGAAACATTTTTCCGTAAAAATAATTTTTATGGTTCAAAACTC[T/C]AATTTTAATTTGTCATAAAGGAAATTATGTGATCTAATGACTATATTTATTATACTTAC	T/C	7	131041216	0.522
7132553862	CACAGTCACCAAAACAGCGTGCAGCTCTGCTCTTGCACCATGGCTACTGAGACCTTAAAC[T/G]TGGGCTGGGCTCTGTACCAAACCCCAGGATGGATGTCAGGGGGCACCTGGCTCTTGCTT	T/G	7	132553862	0.924
7134076525	TCTGAAATCACCTCTGCTCGTTTAGAATAGATTTGATAGCACAGGAAACTTGTACATGAAG[T/C]TTGTATGAAATCTGGGAGCGGAGCACCTCACCGTTTTTCTTTTTTGTTGTTGATGTTTCTA	T/C	7	134076525	0.945
7135617602	ACAAAGTTCCAACTACTGATGGTTTTCTGTGTCGGTAAGCACGGTCACCAGTGAAGGCCA[T/G]TGTGCCAGGGCCCCTTACGGGTCTCAAGGATCCTCTCCCCCAGAGTTCAGAACAACTCC	T/G	7	135617602	0.609
7138802842	CATGAACACTGAGCAGGAATGGAGTGCTTTCCATCTTTATCTATAGATACTTACACAGATC[A/G]GCCCTCATATACAGACTCATGTGTACATGGTAATTTTAAGGTTATGCTGTGTGCTGTGTCT	A/G	7	138802842	0.587
7140344560	TCAACCCCACCTCTTTCTCTTTTCCCCAGCTACTTTATTCACCAATCACAATCAACTGGG[T/G]CACTCAGTGTCTTATGAGCAGACTCACTCGTCTTTGTGGCAAACAGCCTTGGGGGCCCA	T/G	7	140344560	0.5
8000035169	TGTTTTGTTTGTTTGTTTGTTTGATTGTTTTCTGGTTCATAATGTCTGTAAGCTCCAGTA[T/C]GCCTCTATTTGGTTTTTCTTTGTATAGCCTGACTATTGATGAGAGTGGGTTATTGAAGT	T/C	8	35169	0.658
8001536108	TCTAAAAAAAACTGCAAGGACAAAGATGGGTGAGAGACTAAGGGAAAGACAGTCCAATGA[T/C]TGGCCCAACGTGGGATCTATCTCGTGGGGCATGTACTGAGGCCTGACACTAATACTGGT	T/C	8	1536108	0.637
8004634330	TGTCGGGCTCATGTGACTATGGTGAAGTGACTTGATAGCCTACAGAAAAAGGACTCTTCTT[T/G]CCTAAGGGGTTGGGGGGAGGCTCTATCATGATTGGCTGGCTATTTGCCTGCTTTCAGGCTA	T/G	8	4634330	0.94
8006157839	ATACACAACGTGGGATAGTCAACAGTTTAGCTCTGCCAAAACAGAGTAGAGAAGTCCTTAA[C/G]AGTTTCTTCTTCATTTCAAGGTTTGTCAGATGGTTCTGGAGTGGAAAACTAAAGACAGATG	C/G	8	6157839	0.698
8007676104	TCTATTCATACAGAGGATCACGTCCTATTCATACAGAGGATCACATCCTATCCATACAGA[T/G]GATCACATCCTATTCATACAGAGGATCACATTCTACTCATACAGAGGATCACATTCTAT	T/G	8	7676104	0.672
8009250989	TAGAGTATAAGTGTTCTTAACTCTAGGACTTGTTGGTGTGTCAGCTCCATGCGAGCAGTCC[A/G]GGGTACGGAGGTTTAGATTGACCAGCTGCTCTTGCTACAACAACTGACTTCAAATGTAATT	A/G	8	9250989	0.921
8010819817	GGGGGCAGCCGGTCAGCCTCTCATAAGTATTTGTTTTTTTCCAGTACAAACAACCTCAACG[A/C]GGCCAAATGAGGGCTTTACAGCCCCCAATCTCCATGAAAGTTCTCAAAAACAAACTCAAAT	A/C	8	10819817	0.97
8012459253	CAGTCTTGTGGACTGAACATCTACTGACAACTCCTGGATTCTTGGACTTTGCATTGGACAG[T/C]CATTGTTATGGTACCCCAGCCCCTTAGTTCTGTTCCGTTAGAGAACCCTAGTACATATAAT	T/C	8	12459253	0.753
8019389163	TCTTCAGGAATATGAGACCCACAGGTCTCCTTGAGGAACTATAAGCTATAGCTGGTTAAAT[A/G]TTGCTAGAAAAAAGGAAGTCATATTTTTGAGTGATATAATCACTAGCAAATTGCCCTTTCA	A/G	8	19389163	0.652
8021044500	GACACACATCTGTTGTCCCAGGAATGGCAAGATGGGAGGTGGAGACAGGTGGAGTCACAGA[A/G]GCTTCGGGCCACCCAATCTGACCTACTTGGCAAAATTTCAGGCCTGTGAGAAGCTCTATCT	A/G	8	21044500	0.809
8024523304	TGCCAGTCTACTCTACCGTGCTTGACCTTAACAGTCATCCTTCCTTCACAGAATAAGTTGT[T/C]CCCTCCTTCTGTGGCCCTCCCCTACCCCCAGCTCAGTAGAGAGCTCCAGATTCATCTGCCC	T/C	8	24523304	0.95
8029283497	CAAAATGAAGACCATGAAATGTCAAGTCAAAAGGAAGCCAGAGCCCGTCCCAAGTGGCCTC[A/G]TGGGTTTATATCTCCCGTTACACAGATGGTATGCAGGTAAAAGTGTCCTAGACAGGATTG	A/G	8	29283497	0.907
8030788581	GTGTGTGTGTGTGTGTGTGTGTGTGTATTTGTGTATGATTTTTCAGATCTCTGTTAATTAACT[A/G]ATCAGATTAGAAAAACACCATGGTAGATTACTGGAGTGGTTTAAAGGAGAAATACCCACCA	A/G	8	30788581	0.744
8032321266	GCAAGGAGTTGCTGAAGATTGGCAGTTACGGGAAAGGAGAAGAAATCTGGGCGGGACACCG[A/C]GGACCATCCAGCATTCTGTGACTCTGAGCGGGGCAACTAGGACTTGGATTCCTTACTTGGC	A/C	8	32321266	0.758
8038009549	AAATATGTTTGGTAACTGTCAGAGAGACAGAAAGGATGAGGGCTCTAGAAGGCAGCCAAC[A/G]TGTTATCATAATCATGCACATTACCTACAGGTAGGAGGCAGACTAAGAAAGAGGATTTG	A/G	8	38009549	0.992

8039630834	CCACAGCCCATCTACCTAACTATAATATACACTACATAGAGGTGGTGGGATATACATTAA[A/G]AATGTGCTAACATTTTGTATTAAGAATTTTAGTTCCAAGTTTGTAATGGAGTGGTGTGA	A/G	8	39630834	0.639
8041550093	CATGTGATATGCTAAGCAGTCCTAGTATTTCTGTGGAGCCCATGTTGAAAGAAGCCAGTT[C/G]CTTGCAATAGCATTGACATCTTACATTGCCATTTATTTCTATAATTTTTTGATAATGAA	C/G	8	41550093	0.883
8049983558	CCTCTTATCCCACAGGTCTGCCAAAAACCCTTCCCTCTAGAATGAAGGCAGCCACTTTAAT[A/T]TCATTTCTCCTGTTCTTCCTTTAAAGTGGATCTGAGCATGAGTTCCATAGACCATGGCTAC	A/T	8	49983558	0.913
8051530223	GGTCCTCAGGTCTTCTTTGCATTTAGCATATGAGTTCCCTCCTTCCCTTGGCTGAGAAGAG[T/C]TGAGAGGGATGTGACCTAGAGATGTAACATCACATAATGGCCAGGTGTCTGCGCCCATGAA	T/C	8	51530223	0.883
8054887505	TTGAAATCTCAGCACGCCATAGGCTAGAACAGGAGAGTCAGGAGCTCAAATCACTCTCAG[A/G]CACTACTGTGTTTGATGTCATCCAGAGCTACATGAAGCCCTGTCCAACAGGACTTTTTC	A/G	8	54887505	0.928
8059837778	CAGGAAAACATAACATTCAGTCAGTACTAAAAAGACAAGGCGTAGGTATTGTCTATGTGAC[T/C]GCTGTACTTACCTAAGTTGCTGTATGTTGCGCTGCACGGACTCACGTCTGTAGGATCCGAA	T/C	8	59837778	0.871
8064417055	ATTATATGAGCACATAATATTAAATCTGGGGCACAATTATGAAGATTTGATTTCAAATGT[A/G]CTGAGCCTCTAGCTAGACTACTGCAACCCAGCAGGATTTCCCACTGGGGCCACATGATC	A/G	8	64417055	0.792
8066103802	ACTCAAGGACCCACCAGTTACCTCAGTTTCTCAACAAGACTCAAAGGATTGGTTCAGCGTG[T/C]GCCACCAGCTGCACCTATGTCAGCCCGCAGAGGTCAGAGTAGGACAGTGACCCCCTGCCTC	T/C	8	66103802	0.777
8067960675	ATGCCTGTCTGGATGCTGCCATGCTCTCACCGTGATGAAAAGATGCTGTAAGCCAACCCCG[A/G]TTAAATGTTCTTACAAGAGTTGTCTGAGGTGGGAGTGTTGGAGAGGTGGCTCAGCTGTTAA	A/G	8	67960675	0.957
8072132143	CCAATCTAGAGCCAGTCCGGTGGAGGCATTTTCTAAATTGAGCTTCCCTCTTCCCAAGTGA[T/C]TCTCCACCATGTCAAGCTGTCATAAAATGAGCCATCAGGCCAACATACGGAGAAGACCGTT	T/C	8	72132143	0.542
8073971857	CCTCCATGTTTCTTCATCGGTTCCTGCCCCCAGGTTCCTGCCATGACTCCTTTGATGATGG[A/G]CTGCAGTGTGGAACTGTAAGGTGAAATGAACCCTTTTTCCTCCACATTCCTTTTGGCCATG	A/G	8	73971857	0.755
8075515604	GTGTGTGTGTGTGCGCGCGCGCGCATGCACGCGCACGCTTGCATACTTGTGCACATATGTG[T/C]ACACGTGCTCATGTCCAAGCACATACCTGGTATAGAAGATTCACAGAGAATGTCACTGTGC	T/C	8	75515604	0.972
8077062114	CTTGGACTTTAAGTCCTGAATTTCATCCTCCTACAATCAATGATATTATCAATTATGTGAA[A/T]AATGTACAAGATCTGCTAATAGAATGCACAGCAGAAAACAAGAGTCTCAGATGCTCTCTGA	A/T	8	77062114	0.716
8080228166	GGGGAGATGAGCCCTACTAGGTCTCAGATTTTCTAGAGTCAGAGGACTGGCCTGGCCACTG[A/G]AGAGTATCTGTGAAGACACTTTCCGGGCACTCCCTCGGGAGCCTCACCCACCATGCTCAGG	A/G	8	80228166	0.946
8081834042	TAAAAAATGACCTTATCTATGATAAGAACACATACCATGATTGTTGGACATAAACAAATCA[A/T]GATTCAGTTGAACTGGAAGGTCTCTTTCTGGTGCTATGAAGGCTGCTGGAGAAGAAATGTC	A/T	8	81834042	0.91
8083476331	TCTATGGGCACTTTACACACATGATACACAGATATACATATGGGAGAAGGCCCATGCATGT[T/C]AAGTAAAAATAAGTATTGGGGGAAAAAGAAAAACTCTCATCTCAGTGTCTGACAAGTGCT	T/C	8	83476331	0.961
8085387993	AAGGCAACTGGTAGTTCTACAGGAATCAGAACAACCAGCATGGCAGGATACCCATAATGGC[A/G]CCAAAGCGGCGTTAATACCTTGGTGGTAACCAACAGTTCTCTAACCGGACTCAAAGGCTGC	A/G	8	85387993	0.819
8086960225	CTGCTATGAGGTCTAAAACATCTTTAGCCATCTAAAGATGTTCAAAGAAAACTGTTGAGGT[A/T]ACGGAAATTAGAAAGTCTCCAATTTATATGGTAAATAGTTAAAACTGAATCTGAGCTTTCC	A/T	8	86960225	0.852
8088545231	CACCCAAAATTTATATTAAAACTGCAGCAGGGTTACAGTCTCTAAGCCAAATCAGAGACA[T/C]ACCCACACCAAAGTGTGTGGAAAAAGGCTTTCAAAGTCAAAAAAAGCATTTACTCCACT	T/C	8	88545231	0.899
8091656605	TTTTTAAACTGATTAGGGGAAAAGCATCTACATAAATGGCAATGCTACAACTACTCTGAAA[A/T]ATTCAGAGCTCCCCAGACATCAGTGAGGGGTCAGTGTAAGCAAAGACAGGCAAAGAACAGA	A/T	8	91656605	0.979
8096468057	GAATTTCTTATAAAAACAATAGATGATGTTGTGTGGATGGAGCAGAAGTGGGCAAAAGAA[A/G]AATAGGGGAAGTGGAACGAATATGATTAGAATACATTGTATAAAAGTCTCAAAAAACTA	A/G	8	96468057	0.948
8101253044	GAACTCACAGCTTTACCAGGTTGGTTCATTATACTTTGCTTCTCTTTCTGAATATACTTA[T/C]TGTATTGTATATTCTACTAGTGGCTGTATATTAGCAATAATGGCACTGAAAATGAATCG	T/C	8	101253044	0.505
8104337188	GTCCCCCGTCCCCACATCTGCTCCACCATTTGTTTTTAGTATTCTCGAAAGCCATTTTGTT[T/C]GTGTGAGATAAAATCTTAAAGTGGTTTTAATCTGCATTTCTTTAATGGCTAAGGTTGCCGA	T/C	8	104337188	0.704
8109079791	AGTCCTTTTTTTAGCCAGATCACCTACAGATCGCCTCACAGAATGTAGTTATGTGGGAGC[T/C]CTCGGCCCATGGTTGATCCACTCCCACTGTTCCTCAGCTTCACTACTTTGGCTCCTTCT	T/C	8	109079791	0.951
8110621346	CAAATTACCTTTAGAAGCTTAAAAACTCTTCATATGTCTTAGATCACGCAGTAGTAGAAT[A/G]TGTGCCGTTTTACCTAGGGAGGCTGAGCGCATACCAGCAACACTGGAAACCTTATTGGG	A/G	8	110621346	0.987
8112131352	TTCTTCCAAAGCAGGCACACTGTTATCCTGTTTCTGTAATGTGCACAATATGGGCCTGGA[T/C]GCATATGGATGAGCACTCTGTGCCTTTGCTGCAGTGCTCTGACTGCAGAGATGTGACAG	T/C	8	112131352	0.932
8113672002	CACCACACTTGGCTACTTTGGTAATTTCTTAAAATGTTAAACATACATTTACCGTATGGCC[A/T]GGCAACCCTGCCCCTAAGTATCTACATCTACACAAGAAAAAATTAAAGCAATGTCCACATC	A/T	8	113672002	1
8116787492	TGGATTTTCTAGTTTCTGCAAAGTGTATGCTGATTGGCGTCATGACGCCTTAGGGAAGTT[T/G]CGGGCTGGCTTTGTGTGTGTGGTATGTGAGGGACCACCGGATGACATTGCTTCATGGTG	T/G	8	116787492	0.786
8118338566	GACACACGAGCAGCAAGTGTGCAGTAAGATACTGCTGAAGAGCACTCGATTCTTCCAGAC[A/G]TTCCACTACCAACCTGTTTCAACCCCACTTAGTCAACCCAACTCAGGCTGCACAACCGC	A/G	8	118338566	0.882
8120093696	TGGACCACAAGCAACTTATATAGATTGAAAAATACCAACCTGAAAAGTTGTGACGTACAGC[A/G]CCCATTTACATAAAATTCCTGTAATAAATTATAGTGGTGAAGGGTCACAGAGATACTGATC	A/G	8	120093696	0.904
8121605051	CACTCCACTACTTATGTCTGGCTCAGTTCCCACAAGCAGGCAGAACCCCAAGATCAAAAC[T/C]ACAGAGGAGTTCACGTGACCTGTGCACCTACAACTTACCACGGGACTCCAAATAGTTCT	T/C	8	121605051	0.869
8123189500	ATTTAGAAGTTTCTATAGAACACTTTGTAATGTTGAGACTGGTCCCTCAAGGAAGGCATG[A/G]TGGGAGGACCCTGAGGCAGGTAGTCACAGGTGTCCACAGTATCCTAGGCTCTTAGGATG	A/G	8	123189500	0.625
8124822775	GGACCCCCTGCCATGAGCTATCGAGAGCCCCCAGCCAGGTGGGTTTAATCTGCAGCCCTC[C/G]CCTGTACACTCTTCCTACATAAAGCTTCCAGAAGCAGCCCTGGAGGAGAGTTCAGCCAT	C/G	8	124822775	0.974
8126447368	ACTTTAATGGGAAAGGATGTCCTTGTCTAAGACCCTAAGATGTAGTCAGTCTTACTTTTTC[T/C]CCCTCATTAAAACTATTTCTGATTTGGGCTTAATGGTTTTTTTATTAAGATTTATTTATTT	T/C	8	126447368	0.764
8127969315	AGCCTTTCATTACTGCAAAGCAACCCTAGAGTTCGCTGGGCCACTTGCAGATGCAGCTGAC[T/C]CAGCACGGGAGCTGAGCTGTGGTCAGCTTGCATTCATGTCAGGGCACTGCACAGCCAAACC	T/C	8	127969315	0.738
9000007599	TAATATACAAATTGATAGTACATTTGCCTAAGAACTTCTAAGGAATATACTTTGCAATGTT[A/G]GGTTTAGCAATTTTGCAAACAAGAGGAGAAAGTTAAAGTTGGAGTGTACTGTTCATCCATC	A/G	9	7599	0.924
9004638687	AAACTCTGATAGCTGTAAGAGTATGGGATTATATGTTCTACTCCTCTATTCTGTCCCACTC[A/G]TCAGTGTGTCTGTTTGTATCAGTGCCATGCTGTGTTATGATAGTGCTGTGGCACGACTTGC	A/G	9	4638687	0.949
9006153383	ACTTCCGGAAAGGCGGTGTGCTGGTGAACGCCGTCTTCCAGCTTGGCATGGCCAACGGTAC[A/G]GAATGCTTTACCTCTTTTCTTGTTCCGGTGATCTTGGCTCTTCTTCCCAGCGTCCTCCATC	A/G	9	6153383	0.977
9007696760	CATACATCAGAACTCGTAGAAGACTGTAGACCATCTCAATCACCCTCCTTGTTATACAAA[A/G]AGGAAATCTAGCCCAGAAAGGAGTGACCTTGTGCAGTTGAGAAAGCTGAGAGGGTCCCA	A/G	9	7696760	0.956
9009290725	TAGACAGGAAAAAAACTGGTACTGAAAGTGACTTGTCCTCAAACACACAAAGGCCCAGGGT[T/C]AAGCAGGAAGGGAAACTGATTGTATACTAATCCTCTTGTATTCCCTTCCCCCTTTTCCTGT	T/C	9	9290725	0.872
9010824989	TGACCGGGTGTCTCTGCCAGCATCCCTGACCTAGTCTCTGTACCCTCACCTGGAAGCAGGC[A/G]GCAGCATGATCCTCTCCGGGTGACTTCTGCTTCACACCTCCAGTCCCCTGACAATGTCACA	A/G	9	10824989	0.664
9012529501	GTGGGTGCAGGGAATTCCAACCCAAGTCCTCATGCTTTCAAAGAAAGCGCTCTTAGCCAC[A/G]GAGCTACCCTCCTGCCCCAGAGGTATTTCATTTAATAAAAGTTGTGTAGACATAGGGAG	A/G	9	12529501	0.716
9014275270	CATTGAAACAAATATATATTTTAGTAACCCCTTTTTTTCCATCAGAGTGAATCTAAAATC[T/C]GAAACTGCAGGTGGCTAACGATCTGTTTGAGATTACAACATGTGAATAATAACATAATA	T/C	9	14275270	0.989
9018112709	GAATCATGGTTCTTACAGCTGGGGAACATCTGGAGGAAACAAAGTGTGCCCCCAAAGCTCA[A/G]GAAGTATCAAATGCTGAAGTAGTCAGTTGAAGGGCCTGTTTTTTGTTTTGTCTTGTTTTGT	A/G	9	18112709	0.977
9019698843	GGTGCTGGGAATTGAACCTAGGTCTTTGACTGTTCTAAGTGCACCATCACCAAGGAGTACT[T/C]CCAGCCTCTAATGATACTGCCCCTGACTTTAGAGGTAGCAGAGAGGAACTGGAAGCTATCG	T/C	9	19698843	0.888
9022924893	CCATATAATTAATGCTTATCGGGGCGAATCACTTTTCAAAACGAAAGTCTTATCCACCCAC[A/G]GTGACTACCTGGCTCGAACGACCAGCTCACACAAGAGTTAGTGAACAAGATTGGGTGGAGT	A/G	9	22924893	0.886
9024499905	CAGTTTTATTACTGTTTCCCAGCATAATGACACAAGAAGTTTCAATAGCCGGTAGTCATT[A/G]CCTGTCAATTTCTAGTCTCATTCATTGCATTCGGGGAGACTTCCTGTCACATAGATGTA	A/G	9	24499905	0.876
9026201841	AAACTGCCATTTAGATGTAATCTAAAATAAAAGAATAAAGAAAAGATCAGAAACACAAAA[T/G]TACTGTGATTATTGTGCTAGTGTTCACTCTAACTTAACTTTAGAGATTGCCTAGAAACC	T/G	9	26201841	0.763

9028315173	TTACAACAATAGTTTGTTCTCTTTGTTTTTTATATATAAATTAGTGTCTTTAACCGCTAA[C/G]GATCCTGTAAGACAGCACTTTTTCCTTTATTCTTCTGAGAGCTTATCTCTTAATTGTCC	C/G	9	28315173	0.876
9035235062	CTTTGCCGCTGGGAAGCTGTGTGACCCTGAGGCAGGTTGTCTGACTTCTCTGAACCTCGGG[T/C]GTGACATCCGTAAGGCGTGTGCAAATGTTCAGCCCCAGGTAGCGCAGGAGAGATGGGCTTC	T/C	9	35235062	0.872
9038814990	GTCTTTTTTGCCAGTGAGGATGCATCTGTCTCTTTGAGGACTTCCTGTGTTCCAAGCAAGC[A/G]CTCTGCCACTGAATTACACCCAGCCTGAGTCTTAGGTCTGAGGCTGCTTACCTCAGCCTCA	A/G	9	38814990	0.842
9041986839	GGGGAAACTTGGGAAGATCTGGCTTTGGACCTTGGCTGGGAAAGTGTTCCTTCAGTCCTTC[A/G]GGCCTCGCACTTTGCAGACTGCTGGATAGCTAGAATGGCTGACTTGTTCAAGGTTCTCTAC	A/G	9	41986839	0.938
9046897982	CACAGGGAAAGGTTAACAACCTGACCTGAGTGTTTTCGAAAGTCTTTGCATTTTCCTCAG[A/T]GTTGCCCATCTGAACCCCAACGTTCATTCCAAACCAAAAAATTGGAAGGGCATCGTTTT	A/T	9	46897982	0.941
9053618889	TTCTAAGACAGCCAGGGCTTCATAACAAAATGAAAATAATAATCTGGAGGGTGACAGAAG[A/T]AATCACAGGACATCGACCTCTCTTCCACAGGCCTACAGTACAAACATGAGCACATACGT	A/T	9	53618889	0.926
9055129164	AAACTATAGTTTAAATTAGCTCTCACTCTTCATATGATATTTCAGTCCTCGGCTTCGCCC[A/G]TGTTTTCCAACCAACCAGGATTAGTACCATTTTCATGCTTATATTGTAACAGGTTTGGC	A/G	9	55129164	0.774
9056661667	TCTTTATGATGCAAAGTCATAATGCTCAAATGTCAAAAACCATGTCAAAACATGAAGTTGA[T/C]CTCATTACACTATGTTCAATTAAGACGAGGTTCTTTTCTTAAAGGTTTATTGTTTTATATG	T/C	9	56661667	0.858
9059744869	GACAGGTGGAAGACAGCATCTCTGGACATTACCCTTTACCACTCTCCAACTTACTGCCTTG[A/G]GACGGTCTCATTCGCACGGACCTGGAAGCACGTTTTTAGCCAGATTGCCTGGCCATCCTGC	A/G	9	59744869	0.92
9062839938	TTTTTATTTGGCTGCTTGTTTTTTAACCCTGATGTATGCCTACGAACACCTGGGGACTCTC[A/T]TTCTTTTGGGGAGAAATAAGCTGGCATATATTTTATGTTCAGAAGGTACTTTTGCTTAAAT	A/T	9	62839938	0.94
9067629682	ATTTGTAATGAGTTGTCATTTTGAACTGTGAAATACAGAACCACTGTGGAGAGCTAATGC[A/G]CAAAAATAGCAAGATAAAATTACAAGCAGGACTATGTCTTAAATCTTTGAAAAAAACCC	A/G	9	67629682	0.908
9069300117	GCCAGTGTGATACTCCTCTTATTTTCAAGGATACAGATATCAGTGTGAATCTTCACTTTTC[C/G]CTCTGAGTGTTTTGAGCGTCTTTGGAGAATTAGTCGCGTGTGAGATGCTCACAGACATCGT	C/G	9	69300117	0.944
9070857309	TCCCCTGAGTTGTGAGCCATCATAAATGCCTCCTCCCGTGTGTATTTTAGTTCTAGTGAT[A/G]TAAAAATAGTTATCCTCAAGTTAGTGTGTAGGCTGTGTCTTCAAGAAGTCATTCTACTG	A/G	9	70857309	0.732
9072613889	ATATGAACACTGCAAAATGAATCAAGACACAAACTAGGTTGTACCATCCCATTCCCGCCTC[T/G]CTGGAGAGATGAAGAATGCTAGTTTCTTAAAAGAGAAAAGTGTTTTGAAGTTGTAGAGAGC	T/G	9	72613889	0.963
9074172087	TCCCTGTATTATTTTACAGAAGTTTCACACAAGGTAGAGGGTGGAGCTCAGGCCTCCACGT[T/C]ATAAAAATATCTCCCTGTATTATTTTACAGAAGTTTCACACAAGGTAGAGGGTGGAGCTCA	T/C	9	74172087	0.741
9079064675	TGTCCTACTACTGATGGCCAGCCTTGAGCTCTCGGTTGACATTTTATCCCAGAGACCATA[A/G]GAGCACAACCTTTAGAATCAGATCTGCTGTTAAGTGACAGAGATGTCTTTGGCAAGCTA	A/G	9	79064675	0.908
9086630595	ACACTGACTTTACAATGGAGGCCGAGGTGACTCCAGCCCCTCTATACTTAGAGCTCTGGGT[C/G]ACCTTCGACTGAGAGTTTGGACTTGGCATGAGGGTCAGACCTATTTGTTATCACTAAGCTA	C/G	9	86630595	0.94
9090103431	CTGCATGTGAGCCTAGCAAATGCCTTAAAACACACAGCCACCACCGCAGCAAGGGGCAAGG[T/C]GCTTCCCTCAGCCTAAGAGATGCTTGCACTTCTGTGGCAGTCATTCTTGACCCTGACCCCA	T/C	9	90103431	0.811
9091773328	TGTGTAAACATTGTTCCCTAATTGTTCCTCAATTGGTCAATAAAGCAAGTTGATCAGACA[A/G]TGACTGAACAGGAGAGCAAATAGGGCTGGACTTTCTCCTAGACAGAGGAGGGTGCAGGT	A/G	9	91773328	0.969
9098131050	TCCAATGTATTCTACAGATGTGGTAGAAATGAGCACGCTTGTTTTCAGGAAACACTTCCTA[A/G]TCATGCTAATTTATGCTTTTCACAGTCAACGTTTTGCTTTACAATTTTGTCCTGAACAACA	A/G	9	98131050	0.931
9099767344	TCCTACATATTTCAGTAAGTAAACAGGGTGATTATAATGTCCTTCAACCACCGAGTCCTT[T/C]CTAATTTTGACTTGTATCCTTGTCCTGCTATTGGTTTCGTTATTTTCGCACATACATGA	T/C	9	99767344	0.985
9104399314	TCCGGGCTTTTAGAGAGAAAGAGCAGAGCCAGATGTGAACAATGCCCTCCTTCTTAAGCAG[A/G]TTCATGGAGAATCTAAGTTGTACCCAGATCCCCAGCTGTGAGGCGCACCACGACTGCGATG	A/G	9	104399314	0.981
9105972844	TTATCAGGCAGATCTCTGTGGATCGCAGAACCAACACCAAGGAGAAAGAAATCAGTAGAA[T/C]GGGTTGTCTTTCCAGGAGATCTCGGGCATTGACTGCATCTCAGCCTGTTTAGGAGGTCA	T/C	9	105972844	0.968
9107605440	CTATGAAATAAAAAAGATATTTTTATATAAGAGATATTTTTAGATTTTCAGCAGCAGAAAG[A/G]AAATGTTTCTATCCCACAAGGAAGGAAACAGGAGGGACGTGATGTAGGATCAGACCAAAGC	A/G	9	107605440	0.919
9109154182	AATAATCCAAAAAGAGACATCTGTTTCCAAGTGGTATATTTTGATCTTCAATTCAAAATAC[T/C]TTCCTCATATATACGGGATTTCAGAAAACATCATACCAACATAATTGTGAGTTAAACTGTG	T/C	9	109154182	0.76
9110891808	CCAAAGGTCCCAGGGTTCTGATCCTAACACCTCAACCTGTTATGATCTGCATCTATTGCT[T/C]CTTTCCCCAATATATTCACTTTTAGGCTGTGCCTCATAATTTTTGATGTATTATGCTGC	T/C	9	110891808	0.883
9112476703	TGAGAGAAAGGATGACTGATTCTTCCCTAAAGCCATAGAATGGTAAGAGCCCAGGTGCAA[A/C]CTGAAGCCTGTGTATGATTTAGCAAGAAGATCATTTGTTCCTCTCCACGGGAACAGGGC	A/C	9	112476703	0.971
10000138483	GATCTATCAAGATATAAGTCTAATAGGCTTCCAGGAGGCATTCCAGGGAGATTCCTTGGGT[T/C]TCAGAATAACCTGACTACATTTTGACAAATTTGTAAGCCAGCTGGCAGCCACTTAGAAACA	T/C	10	138483	0.991
10006775353	CCTCTTCAGGTTTCCCAAGCCAAAATGGCCCCAAATCCAAACCTCCCAGTGGGTTTTGTG[C/G]GGTCAAGGTGCTCTGAACACATGAGCATGGAATGGAGGCACAAACCGGTAGTCTCAGGC	C/G	10	6775353	0.918
10008363675	AGGCTGACGGGCCAGCTTGGGGCACCGGGAGCCTGCTTGTCCCTGCCTCCTCAGCACTGGG[A/G]TTACAAAAGCATACCACTATGTCCCCACTTTCTGTGTGGATCCTGTGATCAAGTTCAAGCC	A/G	10	8363675	0.783
10009957612	AAGGCACCTGGGAGAACTTTGTACCATGCAGGGGTCTAGAAGTCAAACATCACAGAAAGA[A/C]TTTGGGGCTGGAAAACCTGCTAAGTGTTGTGCTTCCCGCTCAATTATTAAAGTTGATTT	A/C	10	9957612	0.973
10011516717	GGCCTAGGACGTTTGTAGGCCAAGGTTATTGCTTAGAAAGAGAGCGCTCGTCTCCATGCAC[A/C]AGGCTCTGGGCTCAGTCTATGCCTGTATGAGGACTTCTTGGGAATCTCTAGTTAAATCCTA	A/C	10	11516717	0.804
10014700063	GCAGTCGAGGGAGGCTTCTGTGACTCCCCCCTCCCCAACTCCCAGTTTTCCTTAAGGCACC[A/G]GGCATCTGGGAGAAGGGCCTGACCCACATTCCTCAATGCTGCTGGCCCCAGCAGCTCACTC	A/G	10	14700063	0.811
10017759555	GCCCCTTCAAGGTGCTAATGGCTTCAAGATGGGGTTACCCCTTCTGCCTGATCCATGGCTC[A/G]CGGCCCACTTAATGTTGGGGGGCAGGCCATCTGCCACAGCTGCAGAGGTTCTTTGCTTCTT	A/G	10	17759555	0.61
10020870209	ATGAACACACACATGTGGGAACACATATTACTACCCATACCTTGAAACAAGAAAGAGAATA[A/C]TACTAAAAAAACATAAATACATTCAAATGGGCTTTTTAAAGAAAGAAAGGGGAAAGTTCGG	A/C	10	20870209	0.744
10027170584	CATTTTGCTTCACTGAATTATAAAAGCAATACATTCTTATTAAAAAAAAACAGTAAGGAAG[A/G]GGCAATCAAAAGATAAATGGCAGAAATAGGCTGTAAAGTGGCATCTTCAATCCCATTTCCC	A/G	10	27170584	0.849
10028719102	GCTGCAGTTTTGGTGAAGCGACTGAAACGCGCTCTGCCTTCCACATTCCTCATAGCTTACT[A/G]CAGAGCTCCTCCCCAAATCCACAGTTCACAATGGGAGACCAATTTGTCTCCCTGGGACCTC	A/G	10	28719102	0.976
10030374109	TAAGGCACTTCCAAAGTTAAAGGACCTTGCAGAGAAGCAGGATAAAAATGACAGCAGCAG[A/G]AATGGGAGATGGGCAGTTAAGGGCATTTGCTGTTCTTGCAAAGGACCTGACCCGTGTTA	A/G	10	30374109	0.818
10031907156	AAAAAAAAAGAGCTTCAGTCCAGTTCTCTATTCAAGGACTTCTAGTTTTATGGAATTTATT[A/T]TTATAAGGCAATTCTGTATACATATATCTTCTAAGTCAAAGTTCTCTAAGTTCAAAGTGTG	A/T	10	31907156	0.537
10033524496	TGTAGTAGGATCCATACAATTCCCTGGAGTTACAATATTGGTACCTACAAAACAAGGCAA[A/G]ATCTATATTACCTCGGGTTCCATCTTGAATATACATAGGGCACAGGGAAGGAATTGATT	A/G	10	33524496	0.888
10038246551	GCTAAGCCAGAATAGAAATAACCAATTAATTTCCCCTTTGAAAACAATTCCACGATGTTCC[A/G]TTTTCCGTGCCAGCCTTCCTCTGGCACATTTGTTTTCTGGTCTCAGAGGTAAGCGTGCACG	A/G	10	38246551	0.916
10039818446	ATGTGGTAGAACTGAGTTCAACCTGTCTTGTCTTTTACCAGATTTTTCATTTAAATACAT[A/G]GAACACATTATTTTAAGCATATGTCGGGCTTTGGGTTGTTTTGTCTTATGATGCGGGAG	A/G	10	39818446	0.829
10041439179	TGTGAAACCTTTAGGAGGCAGGCTCTAACTCCCCATTTCTCCCTCCGCCTGACTCTCTGG[A/G]GTTTGCCCTCATGGCTCTTGCTGTGCTGCACCGTCCACCCAGGTGGAATTTGAACTCTT	A/G	10	41439179	0.662
10044705113	GTGTATATATTTTACATAAATCTGACTATTTCATTGGTTTTCCTTTACAGGCACAACAGTT[A/G]TATAAGAATGTTGGTCTGTTTTTATAATGTGACGTAGTTTAATGACAATTTTGAAATAGGG	A/G	10	44705113	0.71
10046241978	CCCAGTGGCCACAGCAGGTTGGAACTCACTCTCCTTCCTCTCCTCCACTGTGGTCTCTCCT[A/G]TCTCACACTCAGCCCTGTAGCAGTCTTCTTGAAGGTACCCCTCCTCTGACTACAACTGTCT	A/G	10	46241978	0.836
10047776989	AGGTTCTTATTGCTCTAAACAGCCACAAGTACAGCGGGCTGAGATTCTAGGAGCAGTATA[C/G]TTAGTTTCCCTTCCTGTGGAATCTGGACCTGGAATGTCTCCCGAAGCCCATGTGTGAAA	C/G	10	47776989	0.943
10055969848	TTTGCTGATTGTAGTGAGGCAGGTGAGTGCCCCGGGACTCCCGTGAGGTTTGGGGCTAATT[T/C]ACAGCTTTTGTAATTAGTGCACTCTTCATGGGCATGCGCTGTATACACACACACCCCTCCC	T/C	10	55969848	0.992
10064286854	GGGACCAGATGCACAGGTACCACCCCCCACCTCCCGACACTGAGCCTGGGTTCTCCACATC[T/C]CCACTCACTTAGCTCCTCGGAGAGAGGCGGGAAGTCATAGATGTGCTCACAGGTGTTCTTG	T/C	10	64286854	0.737

10065987410	CACCATCAGGATTATTGGGAAAGGCAAACAAGACACAAGGTGAAAACTGCAGAAGGTACAG[T/C]TTGGCTTTGAAGAACAGAGAGAGGGGGGGGGTGGGCCTCGAAGACCCAGGCAGGCCTGAGA	T/C	10	65987410	0.935
10067577050	TATATTTTGAGGTAGAGCCTCATTATATACGCAAGGTTGGACTTGAACTCAAGATCCTCC[A/T]GTTTATGCCTCTCTGGTGATGGAACGGCATGAGCTACTGTACCTGACTGATCCTGTATA	A/T	10	67577050	0.642
10069114925	CCTCTTGCTCCCCTCTTGCTATAGCCTTGATGCTTAATTGCAAGGGTCCAAAAGTTTCATC[A/G]TTAGCAAAAATAATTATTCTTTCAGCTCCCTCTTTTGTTCTCACTGGCCTCTGTCTTTTTC	A/G	10	69114925	0.969
10070676395	GGTATCTTGCAGAGCTTCAGGGCCACTGCACCTCACACAGCTAGGATTCCCACTGTACCAT[T/C]GTCTCTTGGGAGTATGGTAGGTGTGTTTGTCTCCGTCTCTCCTGCAGAGGTGCTGGCTCTT	T/C	10	70676395	0.922
10072264375	TCGGTATTACCTGTGGAATGAATGCTATCCACCAGGGTCTTCACTGATGCTAGGAAAACAC[T/G]TATGCATACCTGTTTCATGTAGTATAACTACATGTTTCTACACGTCCTGAAGGAGGTTATC	T/G	10	72264375	0.924
10077287817	GAAACAGATAGATGGGCTGGAGTTGGCAGCTGACTTAGAGGGAAGGAGGAAGGGGGAGAGT[A/G]GAGAGAAAAGTATGAGGAACAGGCAAACTTGTCAAACTCCTTTTTCATCCCTCTTCCGCCT	A/G	10	77287817	0.966
10078860043	GCAAATCCTAGTTATTTTTAGAGTACCTTAACACACCCTTTGAAATCGATTCGTAGTAGA[T/C]GGTAAGCTTAAGAGGTTGATGGTTCTGAAGAATGGAGGAATAAGAGAACAGACTGAGGC	T/C	10	78860043	0.92
10080464456	TTATTCCGATTCAGGTTTTGCTTGAAAGCAGTGATTGACAGGACAAGATGATGTTTGATAA[T/C]GATAAACAATAAGATACTTTCTCTATTTCCTCATGTCTCTCTCCTTACTAGACAGTCTCAT	T/C	10	80464456	0.621
10081985153	GATTTCTCTCATAAGGACACTAATTGTAAAGATTCAGTCTATGTGAGTATGACCTAATGAA[A/C]CCTCAAAGGTGCTACTGTGACCATCATACTAGGCACGAGTACATTAATACAGACATTTGGA	A/C	10	81985153	0.937
10085150354	GCCCCACCCAAACGTCAGATCATCTTGGAATGCAGGCTGAAGTGAACCTTTGACCTCTGAA[T/C]GCTGAACTCTGGAGAAGATAGATAATGGCATGAAGATTTCCCAGCTCCACAACCTCTGCCA	T/C	10	85150354	0.819
10086714378	GGGAACGAGTGTGGAGACACAACACATGCTTCTAACCCACATCTGCCCTCAAGTCAAATGT[A/G]GAAAGGAAGTTCTGGGAAAGGAGGAGTAGTGTGCCTGACCGCCTGAGGCCATCAGGTGCCA	A/G	10	86714378	0.866
10088241830	ATGTCTCACCAGCCCCTGACACTTGTTTGTAGTACGTCAGAAGCGATTAACAGAAGTTCTG[T/G]GCGTATTAGCTTTGCTAAAGTTTATAAATATGTGGTTTTGGCGGGATTTGTATTGAGAGTT	T/G	10	88241830	0.876
10089766892	TTCGATCCCCAGCTCCGAAAAAAAAAAAAAAAAAAAGTTCATCTGCACATACATATTCTCTT[T/C]GGAATACGGAACACTTCCCCACATCAGAAACAGGAGGAAGATTTCTAGGGGAACGCCTGAT	T/C	10	89766892	0.989
10091419662	CTGACCGTTGCTCATCACCAGCTGCTGCTTTGGTCTTCCCGGACTCTCCATTCTTTGTATC[T/C]GCTGCTCGCTCTGCAATCAGCTCAACCTCTAACCTATTAGGATCAGAGTGACTTGCCAACT	T/C	10	91419662	0.941
10094502019	GAGGTTTTTGACTGTTGTTTATCAAAAGGAGGTGCACTGGTTTAGAATTCTATAGACATT[A/G]AGTCTACATTAACCACTTGATATATTATCAAGTTGACAAAAGTATTTTATTTCCTCTGC	A/G	10	94502019	0.578
10099943591	TGCGCGTTGCGTGTTCAGAACCCAGGCTACAGAGACGCCGCCCCGTGCAGCTCAGGTGAGC[A/G]CCACCAGAAACCAATCCGTTTCTTTTCGTTTTCGGCTTTGATTTTGAATTGACTTCATAAT	A/G	10	99943591	0.76
10101611833	TGCATATGTGTACAGGTTTGAGTATATGTTCTCACATGTGTGCGTGCATATGATCTGGGCA[A/G]GCATGTATATATGTTCAGGATCCTCTGATTGTATCCCTCAAGCAACGGAATTAAAGCTATC	A/G	10	101611833	0.961
10104629819	TGAACCCCGATTTTCATGTCTGTTAGACAAGCACTCTATCATTCTACATCTGAGCTGTAGT[T/C]CTAGCCCTTATTTTGCTTTTACTCTTTTTTTAATTTATCTTTGTGTGTGTGTGAGTGCACA	T/C	10	104629819	0.766
10106193291	TGGTTTCATTCTTCTGTCTCCTCCCTTCCCAAATACTCTTTCTTGGTGCAGTAAGTACAGG[T/G]GGCAGCTCAGGTTAAAATGGTCAGCAGGAACATGTGCTGGCCGGCCCCTTCTCAAGACCTA	T/G	10	106193291	0.806
10107819766	TTTCACAGAGCAGGCAAGAGGCAGAGGTTGGCACTGTAGATAGTAAATGAATAACCAGCCA[A/G]TTCCCACCTCAGCAACAGGAAGTGCTGGTCATGGGTATATAGTGCATGTTGTCAGATCTCC	A/G	10	107819766	0.958
10109399893	CTGCAAATGACAGTCTTCTGAGAGTGTGACACTGCCTTTGCTGTTCCTGCTGTGTGACTT[A/G]CCATCTCCCCCCTTATCTGGCCCTCACCCTCCAGAGTATCGAGTGTGAGGAAGGGGGGA	A/G	10	109399893	0.834
11000889960	TTTTAATCTTTCTCTAACACCAACTCATGATAGTGACTTGGGTGCCTAGGGCAAGTGAATT[A/T]TGCCTTCTGGCATATAAATGAATGCATCAGGAAAAAATAAATGTGGATACATTAGGCAATA	A/T	11	889960	0.965
11007329190	AGAAATACCCAATTTAATAAAGATGAAATAAAAAATCACATTTTGGTCTTCTGGAAGTAGA[T/G]CCACAGCAGGTTGTGAACAGCTAGATTTGGGAACTAGAGCCTGATCTCAGATTTTCCTTTT	T/G	11	7329190	0.993
11009165141	GGTTGGAACTTGGATCTCATGCTGTGTTCGTCAGTTACCGTGTATGCTTTTGTATCTGTCT[C/G]TCTTTAATCATTTCTTCACATCACTTGCTGTAGAGCTGTTCCGTCTAAACAGAATAAATAT	C/G	11	9165141	0.846
11010882373	AATTAATTTTGAATGGATAAGTGAAAATGTTATCCAACAAATACACAGTGGAGGTCTTAA[A/G]ATGACTTGATAATTTTCTACTCACTCACTGTTCTTCTTAAGATAGTTTGGGATTTGGTT	A/G	11	10882373	0.733
11012641195	CACAGAGGCAGGCAGGCGGGTAGATAGACAGACAGGATGGAGTCCGGTTTCTCAACTTGG[T/C]CATATGAGATTAATCAAGATAAAGTTATAAATACTTAAAGTCTTAAGAAGAGAACCAGA	T/C	11	12641195	0.69
11017619785	GGTCATTAAAGGAAATTGTAAGCCATCTGACATTACGCTTATCACCCATGCCCTGCCTCG[T/C]GGTGTTGAGAAATACACAGCTTGAGGGTGGGTGAATTGGAGTCAGTCTTTTGATGTGAG	T/C	11	17619785	0.639
11019203719	AGGTTCCCAATATCCATATCATCCTGTCTCTGACTGCAGCCACCATTAAACAAGCAACTTT[T/G]CTTCAACACTAGAGCGTATGTACTTTAATTCAGAATATGGATAACATGGTCAGTTTGGACA	T/G	11	19203719	0.962
11022388395	TAATAGTAATTTGGAAATATTGGAGGGAGGTAGATTAACCACATGATAGTTAGGAAGTAG[T/C]TCAGTCATTGAGCTCTTTCTGCCATATCAAACTATAAAGTCTCTAAGTGAGGTTTGTTG	T/C	11	22388395	0.939
11024052265	NNNNNNNNNNNNNNNNNNNCATCCGTGTGACCATGAGGTAAATGACCATTTCTCTACAGTG[T/C]GCTGTGTGGCTGAGCTGTAGCACCTGGTAAGTATGCAATGCATTTCCAACGTGACTTACGG	T/C	11	24052265	0.985
11025582611	TGATGAAGAGAGACATTATGTTTATGCAAATACTCCTCCCCTGCCTCTCTCTCCTAGACTT[A/T]TTCCCTGCCTACCCTTCCTTTGTATCCAATAAAGGGCTTCGCCTTATCCTGGACATGCCTT	A/T	11	25582611	0.724
11030346235	TGTAACATGTGTCCTTGTGGTATGGTGGTGTAAGGGTTCAAAAATCGCCAAAGGAAGACCC[T/C]AGATTTAGATATTATGCAAAGACAAAGAACATTTATTCTGCAGAAATACCAGCATTCAGGG	T/C	11	30346235	0.795
11031854195	TCGTGGGCGTACTGCATTTTACCTTCACGGTGACTCTGATGTGGCTTTGTCTTATTTCAA[C/G]TGAAATGCTTAATTTACCACAAGGGTAGGCACTCAAGGGGGAGCGTTAATTGACAGTAA	C/G	11	31854195	0.92
11033435569	AATGTTTACCTCAAAATATAAATATCGGAGCTGGTGTTAATGACTGCAGGTTCACTCACAC[T/C]CCTACTTTGAAAAACCTGAGTGTGTCACCAACTCAGGATTAACCATCACTGATTTGGGGTA	T/C	11	33435569	0.946
11034966294	ATGGCTCTAACCTCACTCATTTGCTGTCATAAATTTAGGGCCAGGGAGTTAGTCTTCGGG[T/G]TAGTGAAGCGTGACTTCTGATTTTATGCATATATAGTCAGCCTTCCCACTATGAGAGCT	T/G	11	34966294	0.944
11036509230	CCTTCTAAATTATTTATGTTTGTTTCTATGGGTTTGTGCTGCTCAATTAAATTTTGACTTG[T/C]GAATTAGTTAACTTTCAGCTGTATTCTAGGGAGTGTTCTTTCCCATCATAACAACCACTTG	T/C	11	36509230	0.821
11038526316	TTTTTTTTATAAATCCAATACCCCCACGGAGGCTAGCAGACTCTCACGTTGAGTGAATTAA[T/C]TCCATCTGTTTGCTTTCCTCTTGCTGTGGTCAGCTGTGCTGAAAGAGTCAGCATTTTAACA	T/C	11	38526316	0.985
11040166875	CTTCTAACATTTGCTTCAGTTCCATAGATGTCATTACTATTCATAAAAGGCAATAACAAT[A/G]TAATATATAGAGAAGGGTTACACAATAGATCATTTTTACAGCAAGTGTTCATGATGGAA	A/G	11	40166875	0.546
11045566388	TGTGTTAGCAAAGTGGCAGGGAAAAGCAACAAGACAACTCTTGTTCTATTCGTTGTTCCTG[T/C]GTAAGTAAGGAGACAAGACTGGCACTAGCTTTTATGGTAAAACTCTCACAGGATACTTTTA	T/C	11	45566388	0.894
11049064419	TGTCATTACTGATTTTAGTATCACTAATTACAGCACGGCCATCTGCAATCTATCATCAAC[T/C]TGATTTGGGCTTTAAAACTACATAATTGACAAAACAACAAAAACAACAAAAACAACCTC	T/C	11	49064419	0.979
11051063125	ATCATAATTCACAACATATATGTTCATAACATGAACACATACTCTACACATAATCCCTGAG[A/G]CTGAGCTGATATTAATTCCTGTACAGGTTGTGTTTAAACCAATAGAAGCTTATTAAAAAGA	A/G	11	51063125	0.814
11056214836	GGGTGAGAATCTCAGGTATGCACATTCCATTCCCATTTTCCACACCACTGAAACCAAGGGC[T/C]GAAAATGCTGACCAGCGCATTCTGAACTGAAACAACTCAGACACCCACAACAAAGTAACCT	T/C	11	56214836	0.788
11058986195	AAATGTCTTTAAATAATTAATTACAAGAGTCAATACACTTAGTATTGAAGGGTTATAAGAT[T/C]TCCATGTCACAAATACATTAGATGAATTCAGAGTGGCTATTGGATATCCAGAAAACAAAAT	T/C	11	58986195	0.744
11062684008	TAGCTCCTCTTTGGCTCTGTGCCAAGGTGAGAATTGCATGCTAACCCACTGAAGCCAGGG[T/G]CATCTATAGTAAAGCTACAGCAGTACCTCAAGATAAGAAAGCAAGATGTCTCATGGAAA	T/G	11	62684008	0.903
11064270722	AGTGTGGGTCTGTACCACAGCACCTGAGCTCATGCGCATTTGTGAGCCTGTGGGACTTTG[C/G]TGATACAAGGTACCAGTTCTCTGTTACAGGAAGTGTGGTGGGAGATGCAGGTCAGGGCT	C/G	11	64270722	0.995
11065981687	CTCATGGGATAGATCCCCAGTTGGGCCAGTCACTGGTTGGCCATTCATTGCTTCAGTGTC[T/C]GCTCTATTTTTCCCCTGTAATTCTTTTAGACAGGAGCACTTTGGGGTCAAAATTTTGTG	T/C	11	65981687	0.736
11067508537	GAGTCCTCTGGAAGGGCTCTTGACCATCTAGCCATCTCTCCAGCCTAGGACTACTTAAAAA[A/G]CAAACAAACACAAACAAACACAATGAAGGAAAAGATTGTGTGTATGTGTGTGTGTGTGTGT	A/G	11	67508537	0.742
11075571033	GACAGGAGCCTGATATAGTTGTCTATTAAGAGGCACTGCTAGAGTCTTACAAATAAAGAGG[T/C]TAGTACTTTTAGCCAACCATATAACTGAGAAGAGCATCCCCATTGGAGGAGTTAGAGAAAA	T/C	11	75571033	0.606

11080261983	TCATGGCTGGAAGACACTCATCCTGGGCTTTTGTGGAGAGGACCCAACAGTCAGATTCTA[T/C]CACATCTAAAACTAAGTAGTAGATGGTGGCAGTTTCCTAAGGGTAACAAGGAAGGGACA	T/C	11	80261983	0.724
11081846340	GTGGCCTAGAAAGATGTGCTGGGGTCCAGGCCGTAGAAGGTGCTGTTTTCCATCAGGTTT[A/G]GAGCAGCAGGGCCTCTGTCTGCCTTGTCTGCCACTGTATCTGCAGTAAGGAGGCCTGGC	A/G	11	81846340	0.863
11083407429	GCTGTTACTTATACCTAGGGGTATCTGAACAACCAGTTCACCAGCATGTTAAAAAATATAG[C/G]AGTAACCAGGATTTTCTCATTACAAAGAACTTTTCCCAGTTTCTCCTACCCGCTTGCTTCC	C/G	11	83407429	0.813
12000092958	AAAATTGACAATATGAAAACATCTGGCTAGCAAATAACTTATTGTTGAATAGTAAGTGCTT[T/C]TGATAGAACGTAGCTGATTTGAAGTAATAGTCTATAATAGTGAGGGCATACCAGCCTGAGA	T/C	12	92958	0.597
12001618778	GGGTGACACCAGTCCCTATGCTGGGCTCTGGAATGTTTGAGAGTAAAGAAATCTTGCTGA[C/G]CAAAAGTTAAGCAAGTAGGCAGCATGGACATTCTCTGCTCTTGATAGTCATCTAAGTTC	C/G	12	1618778	0.781
12003229231	GACGGTGGACAGTACACTGCAAATCTGTAAGAAGAACGAACAGAGAGAAGCATAGTGAGG[T/C]GATTTCCTAACTACTGTCAGCACCAGCGCGGAGGTCTTCCACAGTGCTGCTCTGCGCTG	T/C	12	3229231	0.932
12006393572	AGTGTGTTTTGTTAACGTTTCCTCCCTGAAGGCTGAGCGCTTGGTGAACAGCTGTCCGCTC[A/G]CACCTGTTGGGCTGCTTTACACCGCAGCCTGAGAGGAGCGCATGCCAGGCTAATTGCATTT	A/G	12	6393572	0.805
12008120463	AAAAAAAGACCCGCAAAGAGGTCAAGATCCACAGGTTGAGAGTTGATGCACTACAGGCTC[A/G]CAGACTTGAACACTTAGCTCTAGCTAGCTGGTGGTGCCATTTAGGGGTGGGGTTATAAA	A/G	12	8120463	0.965
12012794920	TTAAAATAAAATTTAAAACAGATAAATAAATTTAAGAGAAACAAAACGACACATGAAGGC[T/C]GCTTCTATATAGCTGTGCACAGCACGCAGGAGTATTCATCGTCAGATAAACCGTTAGAC	T/C	12	12794920	0.959
12015944442	CTTGCTGGCCACCAATCTAACGTAACTTGGGGAACACCTACCCAGTAAGAGATCCTGTCT[T/C]AGAAGACAAGGTGGATGATTCCTAAGGAAAGACACTAGTGCAGGCACATGCATGCACAC	T/C	12	15944442	0.757
12017543910	CTGGAAATGCAACATGACTTATTTCTTATTGCATTTATTTTTGTAACAAGGTCTTGTTGT[A/G]TAGCTCTGGCTGGTCTTGAATGGAGATCTGCTTGCCTGTTCCTTACATGCTGGAGTTAA	A/G	12	17543910	0.996
12022724423	TGGCGGTGGAGGGGACGCCCAGAAGATCATAGAGAGCGTTGCGTGAATAAGTCCTCACTCG[T/C]GAGCACAAGCCCGTGGCCGAACTTTGAGGCAAGCCCTGGACCTGCCACAATCTCCGTAGGG	T/C	12	22724423	0.824
12025836114	CCAGCCAGCCAAGGCTTAGTGACAACCTGTCTTAATACAAAACAGAAAGGCAAGCTCGCCT[A/C]GTATGAAGGCACACATCTGCAATCCCAGCATTCCACAGTGGAAGATAAGAGAAACGGAAGG	A/C	12	25836114	0.534
12027351891	TGGATGCCTGTCATCTCCGCCGAGATGAAGGCTGAGGCAAGAGGATCCAGGCCAGCCCAAA[T/C]TACATAGCCAAACCCAATCTCAAACAACCGAACTGATAACAAGTAGTAGTAGTAGTAGTAG	T/C	12	27351891	0.848
12032286665	TTTTTTGTATCTTCTGGAAATGTACTCTCCATATACTATTTTTATTACATTTCTTTGTGTT[T/C]GGATGTATGTGGACCACGATTTGTATGTCCCACAGTGGAGGAGTTAGTTTTAAGGAGCTAG	T/C	12	32286665	0.733
12033820507	CTTGTGAAACAAGAATGACACTGGGACAACTTTCAGAGCTCTGGTAGGAAGACAGTTGCT[T/C]TTTTGGTTAACATCGCCTCTGTCACATCCACAAAATTCACCTTCTCCAAATCCAGCTTC	T/C	12	33820507	0.94
12035529975	CCTGACTCCAGCCTGCTGGCTGCAGGGGAACTTCACACCTTCCCCTGCTGGACTGCTGGTC[C/G]CTACCGTGCTGGTTCTCTTCTTGGTTGACCTCCAGCTGCTCCTGCTGCTCTGCTCTCCCGC	C/G	12	35529975	0.766
12037049230	GACACTACACCGAGAAGCCTTGTACACACATGAATTCATCTACTTACACAGTCACCTTACA[T/C]GAGGTAGGTACTTACCAGTAACCCTGTTCAGATAAACCACTGACACAGAGAGATCAAGGAA	T/C	12	37049230	0.717
12041773251	CACCAGGCCGGGAGAGACTATAGGGTAGGAAAGGAGCTGTGTTTGATGAGGAGGAAAAGTC[A/G]ATGCAGCTGAAATATGGGAAAAGAGGAATGGACTAACCTGGGAGAGGCTGGGGAAATAAAC	A/G	12	41773251	0.937
12043298723	AAAACAAGGCCTCTAATGAATTAGCTGTGAGATGAAATGTATGAGAAGCCCAGAGACATC[A/G]AGGGTCCTTTTCAAGGTCACACAGCAAGGCAAGAATAGGGCTCTATTGGGCACGTAAAA	A/G	12	43298723	0.953
12045115666	ACACATCTATGTCCCTGAACACTGAGTCCAGAGCAGGACACAGTGACGTGCTCCATGTCT[A/G]CCTGTGAGAGGGATAGATAGAAGGGACAGGAGACAGACACACGTAGGACAGTTGTCATC	A/G	12	45115666	0.994
12046643613	TCAAAACGAGGGCTAGCACACTGACAGCTGTCACTACAGACACACAGCCTCTGACTCCATC[T/C]GTCTTCTGTGACTGACGACTACCTATGAATGAAGGAACTCACCAATAATAAAGTCAGCTTA	T/C	12	46643613	0.959
13004993693	GAGAGCAGAGAATAATTATTACAAGGCTTTGAGCATTGCATCCAGTAAAGAGTTTGGAGAC[A/G]AGACCAAAAAAGAACTAGGGAGTGCTAAAGCACACATAGGAGCTCTGTGTATACTCAGATT	A/G	13	4993693	0.94
13008708707	GCTGACAGTTGTTAAATTTACTTAAACAGCCAGAACATACTACAGAAGGGCAGCCTAGTTT[T/C]TTTTGCTAGGAATTTTATATTCTTATAGTAAAAATCAACTTCCAAATATTCTCTTAGTCTC	T/C	13	8708707	0.794
13010273719	CTTGATGTTCATTAAAGAAAAAATCTACAAAGATGACATCTCAATTATAATATCTATGCC[A/C]TCCATACAAAGGTACCCATATCCATAAAAGAAACATTATTAAATTTTAAATTGCCCACT	A/C	13	10273719	0.518
13014933245	TAGCCCCAGATAATTTGTTCTGTTTCTCGTACCAGGGCTTGTTACCCATAGGTTGAGCAGG[A/T]CTTAATTCCAATTATATGATACTGTTTACTGCCAATATATGAGTGCCGCAATTGTACTCAC	A/T	13	14933245	0.83
13016609621	TGGAGGCATAAGTCCTTTCCTGTATTCTCTTGGAATGGTGATTTCATCCCTGGAAGCTCTG[A/G]GGGTTGTGGTTAGTTGATATTGTGTTCTTCTTATGTGGTTGCAAAGCCCTTCAGATATCTT	A/G	13	16609621	0.523
13018411493	TTTAGGGCTACTTCTACGTTGCCTGTCATAGTATCTGGTCTTGTTGAAGTTCATGATGGTA[A/G]AAATATTTGAAATCTTCCTTAATCAGATAACAATACCCTGATTTTACAGAAGAAGAAGGCG	A/G	13	18411493	0.901
13023383719	TCCACTGTCTTTTGAATCAGGCAATTAATACTGAGATACTGTGCATTTAAAGACAGTGATC[A/T]GAATCCTGATTCTTGTAAAAAATGTTATACTTTGGGGTATAACTTTAAAATTGGAGCACTT	A/T	13	23383719	0.781
13026804728	TAACAGATTTTAATATTCATTTTTTGTTCTGTCCATTTAGTGCTTTGGTTATCATGAGGCA[A/T]GTGTATTTTCTTTTTGGTCCAATCTGTTTGGTGTTCTATATGTTCCTTGTGCATTTATAGT	A/T	13	26804728	0.695
13036488082	GAAACAGCCCATCTACTTAAATTATACTTTCTTAAACTTTGATTTATTGTCGATTTTAGGA[A/G]CAGAAGCTACAGATACTTAAGCTTCTTAAGCTTTTCCCTTTCACAAAGCTATGAGATATGG	A/G	13	36488082	0.724
13039921157	TGGAACTGGGTTTGCAGGTCAGAAATATCCAGGTGTTTAAGAACAGTTGGGTTTCTTAAGC[T/C]GAAGAGCTGTGATGAGAAGACAAAAAGTTACATTCCCCAGAGACTCCTCTATACGGTTGCC	T/C	13	39921157	0.905
13041625844	TACAAACAAGTTAATTTCAGTTTCAGTATCTTGTTCTTTGAATGGGGTGATAAAAATCTA[T/C]AGTCTTGCTCCTTGGGTCCTCATAGCCTTTGTGATTTGCTTGTGTGCACAGAATTGGTA	T/C	13	41625844	0.962
13043391300	TTTCCCTTTTCTTTTTACTCAATACTGTACTGTAAGTCTTCCAACACATAAAAGGTTTTAA[A/G]GTATCGTAAAATTGTGTATATCTTACTGGTCAGTGGAACCTGGATCTTTCTATAGTTGAGC	A/G	13	43391300	0.652
13051499112	AAACATTTGAGAACTCCTTCAGAATCTGAATTTGAAATAAAATGTGGGAAAGGATTTCTCA[A/G]TCAATTAAATATTTATCTTAATAAGCCTGGTAAAAGAGATTGTGAATCAAAACTAAAGTAA	A/G	13	51499112	0.739
13053045095	TTGAATCTAAAAAGACAACATTTGAAATTTTGAGAGATCAAGTTAAAGTACTGGTTCAAGA[T/G]GAGCCAATAAAAGTACAGTGCTAAATGCAAAATCAAAACTTTAGTGATTTTTGTCTATAGA	T/G	13	53045095	0.844
13057797660	TTCTGGAGAATCTCACAGCCCAAACAGCTTGCCTTTTGAGGCTTGGGCATATGCTTCAGG[T/G]ACTCTGTAGCCTGCTTTGTATGAATTCTGTGGCTCCCTTCCCTCTGCCAAGACCAGCTG	T/G	13	57797660	0.857
13064090867	AAGAGTGTAATTTTCAAGCTTACATCTGTCGAGAAAAACTGAATGCAGAAATACGTGTAC[T/C]GGGAATTATAAACCGCTCTTAGCATCCCTTTTACGGAGCATGAATGGCGCAGTTTGAAT	T/C	13	64090867	0.928
13067234922	TCCATTCTTGCTCCGGATTGGCTCCCCCAGTCGCATGGCTTTGCGACCCCACACCTGGCT[A/G]CTTTCTCACCACCATCCTGCTAAGAGCCTTTCCTCAGGTAAACTTTTTAAAAACTAAAAGC	A/G	13	67234922	0.708
13068987423	GTGGGGAGGTATTGAAGGACCCCTTTCCTCCCTGAAACTACATTTTATCATAGGAGAGGAA[A/G]CATGATTCTCAAGAATAAATAGCATATGAATTGCCTGGTCCTCATCAGCTTCTGAAGTCAC	A/G	13	68987423	0.712
13070520624	TTTTTTTTTTTTTAAATTAAGGGCTCCATGCAGGCAACATGGAGGGCTGGTGATGAACTCT[T/C]GACTCAGCATCCTGAATCGCTTTCATTTCCAAATAAAAGACCCAGGGGAGTAGGCCACTTA	T/C	13	70520624	0.797
13073623100	GATTTCTGCTCATTTGGAACTTATCTGTATAACACCTGTTACCAAGCTCTAAGGACCATT[T/C]AAAATCTCATCCGAGAGCACAGTGGTGATGTGATTAGTGTCCTACATAGTATTCTCTTT	T/C	13	73623100	0.966
13075145314	ATGTAGTTGTGTCTTTCTTCCTCTTTGTATGATTGCTTCCCTAAAGCCCAATATTACTAG[C/G]ATGAATTTGGGGACCAGGCTTCTAGCACCAGAACTTTTGGGAGACACATTCACTATAAC	C/G	13	75145314	0.99
13076714709	AGCCAGACACGGTGGTGTTTACCCATAGTCTCAGCACTGGGAGATGAGAGAGGAGAATCC[A/G]GAAGGCTTGGTAGGCAGCCAGTCTAGTCAGCCATTGAGCTCTGGGTTCAGTGAGAGACT	A/G	13	76714709	0.549
13078283339	TGTGCTGAGATAGATGTTCTCCGCCCTGTGTTAATTGGATTCACAGGTAACAGTAAAGAGA[T/C]GGATACTTCCCTTTTTGCATTTAACAGATGAGTGTAGTTGATATGGCAAAGTAAATATTAA	T/C	13	78283339	0.912
13079883630	AGCGACATTAACTTTTCTATAGCAGTCCTGGTGTCCAGGACGTGGTCTAACTCCCTGTGG[T/C]GGCTGTACTGGTGTCTAGAGCACTGGTCCTTGCAGAGCTATACTGGCTGTGCTCTGGAT	T/C	13	79883630	0.966
13081709022	TTGTGGAAGTGCAGTCTCACTCTCCAGATTCGTAATCCACCTGTCAACACCCTATCTTCTG[C/G]ATTCTTGCTCCAGCTTGTCACTCAGAATAGACAATCCTAGCTCCATCTTCATGTACTACTA	C/G	13	81709022	0.966
13083620119	CAGTGCCGCAGCCCCCTGAAAAGCCGACAGGGAGTAGGGACAATCTAGCGCCCTCCCTCC[A/G]GAGGAAATCATTTGCTGCAGGGCCAGAAAACTCTGGAAAGAGCTGGTCACATTGTACCC	A/G	13	83620119	0.836

13087054272	AAAGTGTCTATTCTTTATTAAATTCTAGACTGGTAAATTCTTTTACTCTCAAGCCTTGGAC[T/G]AGTTCCTATCAAGCCTTGGACAAGTTCCTACTAGAAACATATGTTTGATGTTCATTCTCTT	T/G	13	87054272	0.902
13088649999	GCTCTCGACACAATGATCGATAGGAAGAGAAAGTTTGAGGCCATTAAATAAAAACACATTC[T/C]GGATTCTCTTTATTTTAATGAAAACGAAATATACCTATATCTTCACATGGGAAGACAAATC	T/C	13	88649999	0.854
13095577311	TAGCATACGCAGTGAGGTTTTTACTATATAGAATTTTCAGACATGTTATTATCTTTTTTTT[A/T]AATTTGTGGTCAAATCTGTGAGACTCAGTTCAGAGGCTTTACTGGAACACTGAGGGTTTTC	A/T	13	95577311	0.849
13097326077	CCTTTCTACAGACTTTCCCAGACAGCCTGGTCTCCACATGGGTCTTCTTGGCAAGAGCAGA[T/C]GAGCCATTCAGTGCCTGCCATGGATGGCACAGCACGGAGCAGATGGGGACAGACTGCAAAC	T/C	13	97326077	0.824
13104581568	CAAAGTCTATGACAAAGTGTTTTACCGTGTTCTTGGGCGGTCCAAGCATCCCTTCCAATAC[T/G]TTGGCAAAAGCAAGCTGTGTTCAACCACAGTGCTGTTGATAGGTATGACATATCCTATGAA	T/G	13	104581568	0.79
14000060332	GAACATATGAGTCTCTGCAATCACACAGATCGGATTTAAAATAAATGGGAAAACAAAACAA[A/T]TGAAAAGACAACAAGCATACTTTGAAATTTTTCTCAGATTTATTTTTTCCGAATTGAAGAT	A/T	14	60332	0.572
14001569186	GGCAGATGGGATGCTTCAGAGCTGGCTGAACAAGGGTATTGCCCAAGCACTTCCACAGAG[C/G]TCTGTGACACTGAGGTACCAGTGAGGTAAGAAGATATCATCAGCAAGGTGAGAGGGCCT	C/G	14	1569186	0.916
14006164102	TGCTCCTTCTCTTTGAGTTGTGGTCTCTTACTGAGACTGAAGATTCCTGATGCCGGTAGG[A/T]CTGCTGCAGAATGAGCTCCATGGATCATCAGGTGTCCCACCCATTATGGACATGGACCC	A/T	14	6164102	0.821
14007721627	CAAAACAGCATCTGCTAGGGGAGGGGTATGTTTGCATGCCGGAAGGGGCGATTCCCCCAGG[A/G]ACTGAGAGTGCTCTCTCTCTGTCATTGTCTAGCGCATCTGCAGCTAGTACTACGGCTTCAT	A/G	14	7721627	0.852
14009423933	TTGGTGGAGAGGAGGAAGCATTCAGAGGCGACCAGTCTGCCAGATAGCCACAGCATTGATG[A/G]AAAAAGAGGAAGAGTGGCTCTGAATGAAAGCCTTCTGCCATACACACTTCCTCATTAAATG	A/G	14	9423933	0.914
14010936357	TTTTTAAAAACAAATCTGGAAAAAACGTGCACTAACATTTGCACAGACAAGTGTTTTTAC[T/G]CTTCCATTAATAAAAAAATGTGGACTTAAGGAAATCTGTTAATTTAAATAGAACTGCAG	T/G	14	10936357	0.866
14012622412	TCAAAAAATATATTGCTCTTTCTAAACCTCTTGGTCTGTAATCATGAATGCTACCATTTC[A/G]TGGCACCTTTCTTTAAGGCTTGTATCAGATTTCAGGGGCTTCAAATAATTTCTGTTAGT	A/G	14	12622412	0.981
14014207213	AAAGATGGCATTCCATTGTGCGATTGTACCATTACTTAATTGTCCATACATGTGTACATAC[A/G]TGTGGAAGCCATAAGACAACATGAGGTGTCATTTTCAGGAACACCTCCTTTGGGACAGATT	A/G	14	14207213	0.932
14015762319	CTAATCATCTGTAGAAAGGAAAGGACACTGTAAGAGTAGTCTTCTTGCAAGGTTCCCACAT[T/G]GGAGTCCCTGTGTAAGGGAATAGAGTGACTTATAGCAGGGACAGAGTTCATTCTGCATGGA	T/G	14	15762319	0.967
14017380668	TTAGACACCACACAGTGAATACTTTTTCCATGCAGCAAGCTGGCTGCTCCAGCACAAGACC[A/G]AGAGAACCAGTCAGGAGCTTCTGTCCTCTGTTCAGAAAAATATGGGCTTCCCCAGATCTCT	A/G	14	17380668	0.903
14020494696	AAAAATGAAGACAGAACCCAAAGACAAACAAAAGCAGGAGAGAAAGTGGGCAAGGAGAGG[A/G]ATAGCTGAGGCCCCAGTCATGATCGGTCATATGTGTCCTAAGGCCCCTCTAAGAAGGTT	A/G	14	20494696	0.751
14022100230	AAGTTCGTGGAAAACTCTGCAAGTGAAAGTGATAAATCATTTATTGATAAAAAATTGGAG[A/C]GGCACAATGATTTTATTTTGCTGTTATTACCATGTGATTATAATCATTAAACTAATAGT	A/C	14	22100230	0.909
14025264109	CTGTATTGGAATTTAATTATATAAATGAATCAGTGGGATAGAAAAAAAAGCCCAGAAGTA[T/G]GTCTGAATGCACTGACTCTGTTAGTGGTTTTTGCCACAAAGCAGTGACTTTTCTTGGTA	T/G	14	25264109	0.913
14028439601	TGATAGTAGAAAAAGTGTAGTCGTCCTGGCTTCTATTAGCACAGTAGAAGAAAGTTCTTT[T/C]TAAACTGTAAGGTGCTGAAGGGTTCCATCATTGCTTTGCTCTGATGTGATTTCTGGGAA	T/C	14	28439601	0.912
14030319736	TTATCCCAAGATCTGTTGCCTATCTGGAGAATATGTTTTCTATCAGGGCTGTCTTGTTTGG[T/C]CTCAGTGATACAGGATGTGCCAAGCCCTGCAGAAACTTGTTGTAGGGGGATAACCAAGGGT	T/C	14	30319736	0.905
14033491220	AGGAATTTACATTGCTGAGATACGATATCCAGTGTACTTGGACTATGCATTTCCTACATGA[T/G]ATAGGCAATAAAAATATATACTGAGTGGTGATAAGTTCTGTGAGAATCTGAGGATTACAAT	T/G	14	33491220	0.806
14035022703	AATGTGCACGTTTCTCGGCACACACAACACATCTACCGTCTTCGTTAGGATTTCTACTGC[T/C]GTGATAAGACACCATGACTATAAGAAACTTGTGGATAAAGGGGTTGATTTTTGTCCTAC	T/C	14	35022703	0.519
14036554401	AAGTGCTGAGAAATGGTTCTCTGACCCGGAGCTGGGGTTGAGGAGTTTGGCGGTCACTGA[A/G]CTCTGCCTCAGCTCAGACCTCATCTGGCTTCATCCCGCTCACCCTTCCTTCGCCTGTGA	A/G	14	36554401	0.77
14038119728	CTCCCTGGGGCTGTTTGACCCCCTGTCAGTTTCTATCTCGGTGTGTTTAGGACAAGGCTC[A/G]GAGTCCATCAAGTCCCGGGCGCTCCTGCTCAGTATGTGGCCTTCAGGAGGAGCTGGGCT	A/G	14	38119728	0.876
14039687344	AGAAAGAAATAGTGAATATTGTTGAGAATAATAGGCTTTGAGATAATATGATATGGTATGG[A/T]AAACCATAAATTTAACAGCCTACCTTCCTCAACTACAGAAATTAAAACATATCATTAAAAG	A/T	14	39687344	0.797
14047726412	ATTTTCAGAGGAAGTTGTTTTGTCATTTCCTACATTGACTTACATTTGGTGCAGAAACAAT[A/T]GAGCCTGAGGAAGCGGGGGTGAATCCTCATAAAGATTCTGTAAGGAATCAGATCCATTGTG	A/T	14	47726412	0.883
14049720566	AAAAACAAAAATACCTTGTGGGTGCTGGTATTAAAGGCATGTATTAACTCTGCCAGCTTT[A/T]AGGTATTTCCAAAAAAAGCAAACAAACAAACAAACAAACAGCCTTATTACAGTTGCAGC	A/T	14	49720566	0.715
14051659098	TGAACACACCTTTTAGGGCCAGAGAGTGGAATGTGGTAGCAATATTTGGAGGTGTGAACT[T/G]GTTATAGTAGATGTAGCTTTGTTAAGTAGGTGTGGCATTGTGGATCTGGGCTTTAAAAC	T/G	14	51659098	0.659
14054243535	CAAACCAAAGCAGGGAATATCATTCTCAGAAACATCGAGGATAATGTTTGACTACATCTAG[A/G]CATCATGGACCAGTTAAGCCAACACATGAAATTAAACATCACAGTGTTGCAACCATTCCAG	A/G	14	54243535	0.859
14057388340	CAGCCCTTAATGCACATATTATATTCATGTATAAAATATTTGAATTTTAATTCATTCATC[A/T]GTGTACCATTATAGGTACTTTTGTGCATTCAGAATGTAAAATATAGAATATATTTATAT	A/T	14	57388340	0.708
14059067499	CTGAGAAGAAAACACTGAGAACTATATCTAAATAAAAATGTGTTACCTCTTTGATACCTGT[A/G]TTATTACTGTTTTTTAATAATCATGAGACTTGTTGCCTAGGTATCACTTATGGACAAAATT	A/G	14	59067499	0.648
14064271419	AGATAATGCTTGAGACACTATTCTCTCTTTTTCCTTTTTTGATTTATGCTCCTGACCTGT[A/T]TGCCAAGAGAGAAGGAAAATCAGGAATAACAAACAGATATCTTATGTAACAGGCTGAAA	A/T	14	64271419	0.819
14065908860	ATAAACTTTTTCTCCCTCCAATTGTTTTTCATCAGGGTTTTTCATTATCACAATAGGAAA[T/C]GTAACTGACACAGCTTTGAGCTGAAATGTAGGATAGCCCTCCTTTTTGGTGGTCATGCT	T/C	14	65908860	0.925
14067446669	GGAGATAGAATACAAGTTCACCCAAAGGGCAACTTTTAGATTTCCCTTGTTTTTTCATCC[A/C]TAACAAGAGACTAACATCCTGGAGTTCAGTCATATTGTGATATTGACTTTACTTTATAT	A/C	14	67446669	0.889
14069100564	TCTAAAACAGAAACATTCTGCAGGAAAACTGCCCATAAATAGCCTATGTGAGTGAGATAGC[T/G]TCCTTTCAGATATGTTTCAAGTTGAATGGTTTTGTCTTCATAGTANCTTGGTGCACNCTGA	T/G	14	69100564	0.823
14074797749	ACATTAGCACAACTTCAAAAGTCCCATAGGCTATACTAGTCTCAGCAATATTAAAAGTTCA[A/G]AGTTCAAAGTCTTCTTAAATTCATCCAATAATTTAAACATAATCCTCTATCAAATCAAACT	A/G	14	74797749	0.615
14078060932	GTTTATGTGATGGTGGATGTGAATGCAAATACCTTATGAACCAGGGATTAAATAACTTTT[T/G]GGGGGAAAGAATGCCCAGAAAGGGAAGCTCATTGGCCTAACACTATCTAGATGCCTCAT	T/G	14	78060932	0.671
14079645175	GACTTGAGGGTGACAGAGCTCTGGGTAGAAGGCAGAGCATAAGCAAATAGTTGGGGGAGCA[A/G]GTGGATGGCACCAGGAAACCTTGGCGCCCAGGCTGAGATGCCCCTACATGGGAGGCTGCTG	A/G	14	79645175	0.804
14084841644	GGGAACTGGTTTTTCACTCACCTCTAAGCAGTATTCTTGCTAGGTTTAGGGATCCGGCTTC[T/C]TAGACAGCTTAGACCTCAAAGGAGTATCGGTCTGGGCTCACATCTTCACTCACTTGCTTAA	T/C	14	84841644	0.962
14086540454	CTGTTGGGAGCAGCGTACCCAAGCTTACCCCAGTGCTGCTGCGCCCGGCTCACCAGGAAGG[A/G]TCTCATCTGGATAAGGCGTGTGGCATAAATATGGGCGTACTGTCGGTTAAAGCTGCGCTCT	A/G	14	86540454	0.809
14088308546	GAAAGAAAGGACCAGGGCTGGAGAAATGCTCTGCAGTTAGGAACACTTACTGGTATTCCA[T/C]GTCAGTTCCCAGCAGCTCACAGCCTTCTGTAGTTCCAGGAGATTGGGTGCCTCTAGACA	T/C	14	88308546	0.763
14089819261	CTCAGTAGTATGTATTGTTATTATATGCCAATAAAATCATAGAATAATGAATTGTGAAATA[A/C]TGACAAGTCAGGTGTGCCAATCACATATAATCACAGTAGCCGCAGGTACCACTATTGGGTG	A/C	14	89819261	0.911
14092879829	AAATAAGACATGCTGTCTTCTCATAACCACTAAAGCAAAGTCCTGCCTTTATGAGGTTTCT[T/G]TTTTAAATGGAGACAATAGATACCCACAGACATATTATACAGTTTGCTCATTTACAATTAA	T/G	14	92879829	0.773
14094448681	GCCAGAGTTAAGTCCTATACATTGGAGACCTTTCATCTTATCCCATTGTTTTTAAGCATTG[A/C]TTTTACAGAGGCCTCTCATATCATGAATTAAGTTGGTTATGCTTATATTTACTTGTGGCTG	A/C	14	94448681	0.874
14096038202	AGGGCCTGCCACTTCTGTGAGCGTGTGAGACGGTGTTCCTAGTTGTATCAGAACTATTTGG[A/G]GTTCAGCTATCTCTGTAATTCTGTGATGCTGTCATCCTGAGGTCCTGTGTGTGTCAGAGCT	A/G	14	96038202	0.781
14104354286	GAAATTACATACAGGTGGTTGTAATGTATTTGCTAGGAAGACTGGAATTTGATTTCAACA[T/G]TCATTCCCAGAAGCCAACAGTTAGTTGCCTATAACTCTAGGTCCAGGGTATATAGTAAA	T/G	14	104354286	0.972
14109129876	AGAAATCATCAAGATGTAAGCTAATGAGTTTTCTTGCAGAGGAAATAAACACTGGAAATA[A/C]GGAACAATGTCACATTGGGTGAAGTATATTTAAAATTAAATGTAATCGATTCCAGTGAG	A/C	14	109129876	0.989
15000027190	AGGGATAATTAAACAACTGATCTACTGCCTCTACCCCATCATGGGCTGAGATTGCAGACG[A/G]GTGTCACCACAGCTAACTTGACACAGGTGCTGTACAGCTCTGAATCTGGGGCCTCGTGT	A/G	15	27190	0.937

15003160721	GAGAGCCGTGTGGGGGAGGGATGACTTAGGAGACATGTAATGGAGGTAAAATAAAGACCCA[A/G]GAAACCAGAGGACCACGGGGAGAGGGACACAGGAAAAGGCTGAAGGTGGACACTGTGGCAG	A/G	15	3160721	0.972
15004801074	ATGTCCTAGAGATTTACCACCACTGCTTTTTTTCTTTAATAAAGTTGGGGAAGTAAAAGG[A/C]TTTGTTGTGCTTTGCTTTGAATTAGTGTATTCCGTTTCTATGATTATTGTGCATCCTTT	A/C	15	4801074	0.91
15006564128	CATGGTCAAGCTTTTTTCTCGAGTGACTATTCAAACTTGATATTTTATAACTCACTTTCA[A/G]ATTTGAGCCTGACATCTTAAGCGAGATTCTTAAACATAAGTCATGTGCTTAGAGAAACA	A/G	15	6564128	0.805
15010453477	GAAATGTAAAAGAAGTCACCTAATTATTTTCCTGGAATGGATCCATAAGAGTGTAAATGTT[T/C]TTTGCAGATGTGAGGACACCCACGCTTGACAAAGTGGTGGATGTCTGCCTGATTGCTCTGA	T/C	15	10453477	0.991
15011989650	AATGTCATACAGAATCAAGTTTGGCCTTGTTACTACCACCAATGTCAACCAGATAACACTT[C/G]ACCAGGATATGTGTAGTGTACACCCTTGAGCAAGAACATGTGACCTCCTAAGCCATACGAA	C/G	15	11989650	0.946
15016761941	GCCAAAACTATGTTACACAACTTTTGGTATATTGATTTTCACTTGACTATCAGACATGTAG[A/G]TAAATAAAGAAGGAAAAAGAGAGCTTTTAGCTGGTTACCTGGAATTTGTAAGCATTGCCAC	A/G	15	16761941	0.763
15018306647	TATTTTTGACTTGAGTTCTCAGTGGCCGGAACAATTTTAAAAATTAACCACCCTGGAAGC[A/G]CCCACTTAGTCAGGAATCACTGGGATCCATTTTGTTCTCCTTTACTAGAAGTCAGGTAG	A/G	15	18306647	0.957
15020021035	GTGTCAAGAAAAACTATGCTTACTCTAAAACAGAAGTCTGAGGATGAACAGTCTACAGATA[T/C]AGTGACCTCCTGACAGAGCAAGAAAGCGATCTTTAAGCTGAACTCAGCTCAGTCCCTGTTC	T/C	15	20021035	0.619
15021614352	ATGGCGGTTTCAGCCAGAGTAGACGCTAGAACTTCTTGTCCAGGAAGAGAATGGACGTTGA[A/G]GGAAAGCAATGCCTAACAGTTGGACATACCCTGTGTCCTGCCCATATCTCTCTTATCTCTC	A/G	15	21614352	0.926
15023183146	GGAGCACACACTGCTGCTCGTTCAGAAGACAAAAATTCGATACCCAGCATCCACATCGGGC[A/G]ACTCACAACTGTGTGCTACACCAGTTCTAGAGCACCCAACGCAAAGCCTCTGGCCTCTGCT	A/G	15	23183146	0.789
15024877509	TTTTTAAAAAAGCATTTTAAAATAATCATTGCATTTAACCTTATAGGAATCTTCTACAAGT[A/G]TACAGATAGCCAGAATATTGTTTAAGGCCTAGACGATGCCATTCTAATTGCTGTAGAAATG	A/G	15	24877509	0.748
15029215322	AGATTTAATATTTTCGTGTTTTTGGAAATCTTGATTTTTTTCCTCCTTCTGTTGTTTTGAT[A/T]AAGCTTTATTGTTCTTGATAACAACACGAACTGGAACATCAGCCTACGGAACTGTGTTTCA	A/T	15	29215322	0.638
15031761885	AATTTATGTTCCTGGCTTTGAAACTGATTATTTGGCTGCCTTCCTGTGCCCCTTTCCATAT[A/T]CTGCCTTTAGGTAGGGATATCTATCCCATGCTGTTAGCCTCACTACCCCCGACCAATCACC	A/T	15	31761885	0.733
15033388004	TGGGCACAGCCACTGCTTTTTAGATCACTTCAGTGGGCCAGTTCAAGGTCAGCCCCTTTGC[A/T]TTGGTAGCACTGTATACATCTCTGAATGCGTCACTGTGATATGTCTCAGCCCTGTCTTGAT	A/T	15	33388004	0.986
15039297389	ACGAGAAAGACTCAGAAGCTACTATTGGAGGTTTTTTAGTCACCTGAATCACATGGTCAC[A/G]CAATTAGCCACTGGGAATTGTCCCTAGCATTGAGAGGAAAAGAAGTAAATATTAAAAAA	A/G	15	39297389	0.859
15040996673	GAGACAGACATTGCACATAGGCAAATGACAGTTTGTAAATGTAAAAGGGGAAACCTGTGTA[A/G]GCATAAACTTTTCAACCTACTTTAATAAGAGTTCAATATCCCCTTCAATCACCACCCATCA	A/G	15	40996673	0.71
15042940968	ATCCCATCCCCAAGTGTTGGCCTCGTGAGAGCACCGCCAGCCCCATGCATCATGCTGATG[A/G]GCTGAAGTGGGTATTTGGGGGCAGAGAGCTCAAGGAAAGAGTTTAGTTTTTCTGTTTCT	A/G	15	42940968	0.794
15044492765	ACAGAGCACAGTGTCGGGGACTTTAACACACCAAGCAGAAGGAATGACTGACTTAACAGT[A/G]ATTCTCTAAGCATCAGCCACATTTAGGAGCCCGGACGTCCAGAGCAGAAGCCACTGCTC	A/G	15	44492765	0.9
15048583778	AATCTAAGTGATACAAATACAAGTGCTATTGTCTGGTCATCCTGATCACACAGTTCCACTG[T/C]GGAAAAGTCTCTGGAATCTACATGCCCCGTATTTAAGTTTTTTGACTCACCACTCTGAGTA	T/C	15	48583778	0.654
15050563324	CTAAGGGTGGCTGATAAATGAGACCAAAACTTAAGGGTTTGCAAAAGACCACTGTTCTCTA[T/C]CTCCTCTCTGTTCAAATGCACCCCACACAGGGCAGGAGGCTGATCACCAGACACACACCTG	T/C	15	50563324	0.923
15054562452	TTTTAGACCATGCCCAGATACAGTTTTGTGCTGCTTAATGTTTAGCTGCTTCTGCTCCTTG[A/G]AGACACCTGGAATGTTCCTGTTCAGTGAAACTTTAAGACAAAAGCAATTTCAACGAAATAC	A/G	15	54562452	0.903
15056136431	CAAGACAATCCACTTCCTCTGGGCTGCAGCTGCCACATTCATAAAACCAGGGGGGTAGCCT[A/G]GGCTACTCTTCAAACCCTTGTTTTGTTTATATCACTACCTCTCAGAGAGATTGTGCAGGTT	A/G	15	56136431	0.977
15059253615	TAGCAGTTATGTCTCCTTTTCCAGTCTTTTAATTTCGTTTTGTTTGTTCACCAGCTTACAG[T/G]GTAGCTGGCGCCCTCGTGGCATTTTCTTGATTGCTGTATTTATGACACCTTTCGTTTTTTT	T/G	15	59253615	0.883
15060888797	TGTACACACACATGTTTTGGTCAAAAATCAACTATTTCCAGTTTGGAATTGGCTTGCATAG[A/G]GACAATAGAGGATTAGTACAAAGACGAGGGAATCTTTTGCTTTTTCTGTATCTTTACTTCC	A/G	15	60888797	0.956
15073521957	TTGGGGCTAAATTTCTATAGCCTGACCAAAAGTGATATGAGTGCATTTGTGATCATGATCA[C/G]ATTAAGAAATTAGGCTACACATGGACAGATCCATGGCTCCAGTTGCATATGTAGCAGAGGT	C/G	15	73521957	0.981
15079820106	GAATAAAGCAGAGAGAGCCTTTGGCCAGAAGGTATCATGATAGTGTTCAAAGGATAGTGTG[T/C]TCTCCTCGCTCTTTTAAGCTTTATAAGACCATGTGGTCTTGAATTACTACCTCTTGCTAGA	T/C	15	79820106	0.817
15081552396	AATATAGGTGAAAATTAAATTTCGCTATGGCTGCCAGCTTTGAGTGTGCTCTTTACGGAAC[A/G]TAAACAAGCAGAGCTGTTTATGACATGGCAGATGAACAAATATGTACAGAAATAATATCAC	A/G	15	81552396	0.947
15084753970	AGTATTTTAAAGACTTTCCAATGCTTATGAGCACAACACTTTGTTTTCTGGTATTTCCCCC[A/C]CAGAAAACATCACTTACAGTTTTGAATGAGCCAGACCATCTCTATTTTGTGTTTGAATTTA	A/C	15	84753970	0.962
15089805308	GTATCAAATTATGCATCTTTGGAATGAGTTGTATGATTCTGTTTGGTTCTAATAATTGCT[C/G]TTGCATTGACTTGAATTCCAGAAAAACTCATCAACTGATCTTTCACTTTGGATTAATAC	C/G	15	89805308	0.967
15091387257	TACACTATCCAAATATGATAAAGGTGTGAAATTACATTACGAAATATATTCACAGCAAAAC[A/G]AAAAAATAAAACGACTAGATTGATTTAAGCGTTTTTATCGGATAACACACTTAACATTTTT	A/G	15	91387257	0.725
15094560146	ATTACTGAGGGACATAAGTTGATGGAGACATCTGTTTAGGTCTTTGTTGTTCCAAGGTCTC[A/G]CAGGCTCTGTACAATGTCTGAATGTATGTCTGCCATACTCTGTGGGAAGATGCTTTTCTGA	A/G	15	94560146	0.934
15100784346	ATATACAGTAATAACTGGCTTGGGAAGATATGATTACTGGTGGAATAGTGGCACAAACAT[A/C]ATAAGATTGTCCTCATTGGTTCATTTCCTCCATGGAAATAACAATGCTTTGTCCATACA	A/C	15	100784346	0.862
15107141928	TTCCCCAGCAGGGAAAGGCAAGAGGCTGGGCTGCCCTGTGGCCACCACTGTTTTCTTCTTT[T/C]CTTTTCACAGAAAATGGTTTTCCCTGCTATAGCACTGGCTAATAAGAGAAGACTTCACACA	T/C	15	107141928	0.942
16002097566	AAGTTCTACATTTGAGAGAAAATTTTCAGTATTTGGCCTCGTTTCCTTTCCCTCTTACTG[T/G]CTTCTTATTCTCCTCCGCCTTTCTGTAGTCTCCTTTCTCCACATTTCTATTGTATATCA	T/G	16	2097566	0.916
16003714855	CCCTGCCAGGTTACTGTAACTGAGGCACAGGGAAGACAGCCTGTCTGTTTCTGAAGGACC[C/G]CTGTCAGCAGTATTAGGAATATCCCCTTGGAGAGAGAGTGACAAAGATTCAGATCTTCC	C/G	16	3714855	0.984
16006905481	GAAAGAAAAGTAAATAAATTAGACTGTGGCTGTAGCTCGGGTGGACTATTCACCCAGCAT[T/G]CATGAGACTTTGAATCCCAGCACTAAAAAAATAAAATTGATGAAGGTGATCTTATGAGC	T/G	16	6905481	0.901
16008429540	TTTCCTCCTTCAACCTGAAACCATGGCGGGGCCAAGCCTGACCATGGATCTTCCATGGAG[T/C]GCTCATCCTCCTGGTCAGAGCCTAATGTGACCTCATGTGGTCCTCACCCTCTCCTGCAG	T/C	16	8429540	0.787
16011475486	TTTGAGCTTTCTGTCTGCATAGATCTATTCATTAAGGAATTCTTGACTAATACTAGACTC[A/G]TTTAGCTTCTTGGCTTAACACAGTAACATTGAAACTTGCCAATTGTAGCTTGAATTAGC	A/G	16	11475486	0.59
16013144600	CCACTTAATTTTTGTCAAATGGAACCAAGCTTATAAAAGTTAGGCTGAGGGAATTTGTGCT[A/C]CAGGAGCACTCATCATCTTAATCAATTTCCACTCAAAGTGCACACTTGCTCAACTTGGAAT	A/C	16	13144600	0.964
16014682846	AAATATCCACCTCTTTAACCTGATTCTGAATTAAGGACTTAGATGGAAGAGAGGAATTGAA[A/G]TTAACTAACCTGTGTCATTTCCATAACCAGCTACTGGACACATTCACAGCAATAAGGAAAA	A/G	16	14682846	0.901
16017852571	GTTGGGCCTCAGCCCCTGCTGCCTGCTCTATGGGAAAACTTTCACCCTCTGATTCCAGCC[T/C]GGGGTTGCCTCTTACTGACTGAAAGCAGTGAGAGACACCAGAAACCCTGCCTGAGAATG	T/C	16	17852571	0.886
16019450680	CTGTAATGGGATCTAATACCCTCTTCTGGTGTGTCTGAAGATAGTGTAGATAATTTTTTTT[A/T]AAAAAAAGGCTAATGGATACAGCCCATGGGATGGTGTTCTCCTGATTGACTCCTAGGAGAA	A/T	16	19450680	0.726
16021150350	GGGATTCCCTGTCACCACCTCCTGCCTCATCATCAGTGCCCCAGGATTCAGATGCACTTG[A/G]GATCATCAGACTTCATGTGGATTCTGAGAATCCAACCTTCATTCTCACAGACTCATGGC	A/G	16	21150350	0.685
16023115819	ATTTCTGCTGGACAGCGAGCTGAGTGTCGCTAATTCTCTTAAGGTACTCTCTTCTTTCTCA[A/G]CCTCCTGTCAGCTGTAGAGCCCGAGAATTTGGTCAAGTCTGGTGTTTGAAGCAGGTTAATA	A/G	16	23115819	0.933
16028357587	GTGGAGAGAAAGAGGAGTCAAGTTTGAAAAGAAGTCAAGGGTCCTAGAAAAGGGAACTAT[C/G]GATCTACTATTAGTATAGGAAAAGGAACTATAGATGGCATAAATTTTGGATAATGAATA	C/G	16	28357587	0.695
16030342160	TTGCATCCAAAACTGTGAGATTGATTCTGACCAGAGTGGAAATTGAGACCTGTATCTCTA[T/C]GTTCAATAACTATATCCTTCCAGAGATTACAAACCTAGGACCTGCAACCCCTTTGCTTT	T/C	16	30342160	0.718
16032474356	AATATTTCTACTGTCTTGCTATTGAAACAACGTTGTGAGCGCTTTAAATTTCTCAAACCAG[A/T]TTCGTCTGTTATACTTGGCGCTTAGTCATCGTCGTACACAGCAGGACCTGATTAAGAAGGC	A/T	16	32474356	0.894
16041701263	GGGCCTAGCTTCAGCTGATTTGGAAGCGTCCTCCCTGAGTACTAGCTTTCATCAAAAGAA[T/C]GATCTGCTTACTTAGCTGCAGTTCTGTTTTAATTAACTAACAGAAACAAGTACAGCATT	T/C	16	41701263	0.902

16043608855	GTAAAATATTAGGCATTTCTTCCTTTGCTTTTGTAACCTTTTTATGTATCCAGATTCAAC[A/G]AGATGCTCCCAAGATATTTTTTAAATATTTTAAAGAAATATGCATTAGCCAACAATTTT	A/G	16	43608855	0.773
16045256020	GACCTTGATGTGTTTTAGATATCTGCTGGGTGTTTTTAAAGTGAAACTAAGTTTGAAAGG[T/C]GTTTGATAGGTAAACCTCAACTTAAGGAACACACACATGGTATTTCAACATCTGTAAAT	T/C	16	45256020	0.726
16048477219	ATAAAAGCATCCGGAGCCTTAATTATCACCTGGATTTGTTATATCCTGCATACTTTTAAAG[A/G]AAGTTCCTTGAAGTATTTTTATTTTCTGTGTGTGTGTGTGTGCATGAACATGTGAGTGTAT	A/G	16	48477219	0.637
16053321660	TGTGTCTTTCTTGTTAAAGCTGGGCTGGTGATCAGGGTGATAATTTCTTGTACCCATTTTT[T/G]CACCCAGCTTCCTAATATGATTTAATAAAAATCGTTAATGAGTAGGGGTTCCCCCTGAGGG	T/G	16	53321660	0.502
16055002540	CAAAATCAAAACCACTTTAGGATTTCCATTTATATTTGTGCAATTAAAACACGACTAGTG[A/G]ATGGTTGTTGGTAATTGCCAACCTGACGACTCCAGAGTCTCCTGGGGATACGGGTCAGA	A/G	16	55002540	0.886
16057142447	AGGTTCCTTTGGCTAAGAGCTCAGTTGAATGCTTTCGATATTGAATTTATGCAAAGATAG[T/C]CTGTGCCTATTTTTGAAACTGGATCATTAAAGCCTCTAAGTACTTATTCCATTCTCTTT	T/C	16	57142447	0.941
16058682220	TCACGTTATCCTCCAATAGTAGTCTCTCCACTAGTACTATTGAATTTGCTAGTCCTTTGT[A/G]TAACAAACTTCTGGAGATCATTTGTTCATTAAAAATGTAGTCTTATCTTTCTTTGGAAT	A/G	16	58682220	0.861
16060330064	AAGGTTGAGCATTTTGAGCCTTTGCAGACGGCTGAACTCGTCGGGCAGAGCCTGTAGCCC[A/G]TTGTTGTCTAACATGAGGCTCTCCAAACTGGCCAGCTCACAGAAGCCGCTGGGCAGAGT	A/G	16	60330064	0.897
16063605256	GTCTCATTTCCCTCTCTTAGACCACTTTGTGTCTGTGTGACACAATGAGTTTTATCAGAGC[A/C]ACTTGCAAAATCATGGGTAAGAATTTGTCTTTAGGACTATGGATAGTTTGCCAGTGACTAA	A/C	16	63605256	0.91
16066926357	CCTTTCCCTCTTACACATTCCATACCCTTCATTTTGTCTGCATTTTTAGTGTTTTCATTA[A/C]TATATGATAAGGAGTTCTTTTTCTTATCCTATTGGGTGTTCTTTAAGCGCCTCATACTG	A/C	16	66926357	0.703
16070106379	TTGGTTTTTATTGGTAGAATAAGTGTGCGACCTCCTGTTAACCGTGTGACCTCATTGTTG[A/G]TGAACTGGACAGGAGTGTCCCCAGCTAGCCTCGATTTCAATTCTGGATCTTCTCTGGCC	A/G	16	70106379	0.908
16071658140	CACACTGGTACAGAAAGACAATACTACTAATGCAGCAGAGAATTGTAATTGTTTAGGGGCA[T/C]GTATGTCTCCACATGACCTCTTTAATGTCTATAATAGACAGGCATGTGCATCCCATAGCAT	T/C	16	71658140	0.932
16073178584	GAAGCAGTTGAGTGCATTTAACCAGAATCATCCCCTCGATGCTTCCTGTCAGTGATTTCTC[A/G]CCTGCTGGCCTCCACTCTGTTCCTTAGTTATAAAGTCCCCATTTGCCTTCATTGTAGTTGG	A/G	16	73178584	0.931
16074693199	AAAAAAGCAGGGAATTGCTCCACGAGCCTGTCTTTCCAGGCCTTCACCATGTAGAGACAC[A/G]CTGGCTCATTGACCTGCCCATTTTCTTACTTAGACCTGGAAGAGCAGAACCTGACATAG	A/G	16	74693199	0.887
16076230619	AGCCATGGATGACCCCAGTTTCTACCTCCTGAGGGCTACAGTCTTTAAGTGAGTGCCACCA[T/G]GCCTAGTTTATGCTGTGCTAGAGAATCAAGCCCAGGGCCTTGTGTGTGCTGGGTTTGCACT	T/G	16	76230619	0.953
16078170106	CCATGGCATTGTGACCATGAGTGACAATATCCTTGCACATTTCTTCTAACAAGGTATCCT[T/C]ACCCATACCCTAAATATCAGGCAGGGTTCCTGAAGATTGAGCAAAAGACAACTGCCAAT	T/C	16	78170106	0.828
16079970671	CATGATTTTCAGTTGTATACACGGGGCCACGTATCAAACAGCTGTCGATGAGCCAGGCATC[A/G]TACCGAGCTCCTCTAACAAGCATTAATTTGAATTTGAACCATTACCTCTCTTTTCTGATCA	A/G	16	79970671	0.827
16083135970	CAGGTGACAGGCGGGCCTCACTGGGAGACCTGTTTCTCCATGAACACCGTATCATCCTACT[T/C]TCTAATACAGTTGCTTCCTGAGCAGATGTGCGAGCTCACTTATGGTTTTTTTGTGGCATAC	T/C	16	83135970	0.939
16084722774	TTTTTGTTTCATCTCCTTTGATACTATCCCTGCCCTGCTGAAGGCCAGTGCCCCCTGAGAG[A/G]CCTCCCATTCTGTAGACTCTCTCTTTTATCTGTACAATGTGGAATTTCCCACAGATAATAT	A/G	16	84722774	0.842
16086483770	AACTAATGAAGTCTCTATTCCAGGTAAGGAGTTGGTGAACCGGGCTTTGTAGCTGACAAAC[A/G]CGTTGACACGAGGTCTCTTGTTGGGTTTGGGGAATACCTGCACATTCGATGTGATGTCAGC	A/G	16	86483770	0.946
16090046133	TTCTGCTAACTCATGCTGAATAATAACTGTCATCAGTGGAATACCCACTGCTTTAGGGTCT[T/C]GGTTAGAGATCACTCACATAGTATCTTACAGATTCTTTTCAGTATCCCTAAAGGCATTTGT	T/C	16	90046133	0.807
17000144919	CCATTTTAACTATGAACTTGAGCTAAGCAGGTCCCCAAATATCCACCATCTTAAACTGAG[A/C]ATGCTCAGGAAAGTGTTTCCCTATATACATATCTATAGGTTCTCCAAGAAAAATCTCCA	A/C	17	144919	0.924
17009834035	GTGTGTGTGTGTGTGTGTGTGCTCATGTATGATATGTATGAATTCAGGTGTGTTCAAGCCA[A/G]GACAAGCATAAGGCAGTCAGAGGACAAGTGGAGACACCCTCCACCCTGTTCGCCCTGGAAT	A/G	17	9834035	0.842
17011614563	AGATATGGGTCTTCGTCTCGGGTTGTTTTTGGACCCTTTCTTCAAATCAATTGTATTGTT[A/G]GTGCATAGCAGACCCTGGACTCATCTGAGTAACCTGAGTCCTTTTTCAAGATTGTTTAG	A/G	17	11614563	0.973
17020929127	GCATGACCACTTGAGTCTGGATCCCGGAGACACGTGCAGAAAGCTGGGAATGGTGATGTG[T/C]GCCTGGAAGCCCAGAGCTGGGGAGGAAGCACAGGGATCCGATCCACGGGGCTTGCAGGT	T/C	17	20929127	0.698
17022486161	TCAGATTTGTGGCAGTCAATTTTTATTGTCAACCAACAGGACTTAGAATCATCTAGGAGA[T/C]ATACCTCTGTGTGTATCTATGAGAGCATTTCTCAAGAGACTTAACTGAGGAGGGAACAC	T/C	17	22486161	0.701
17024009487	TGAATTCATATTGCCTTTTGGAGTAAATGCCAACAAGAGTATGATTCTGGCACACAATTG[A/G]ATAAGACTGTAATTTTTCAGCCCAAACTTCCTATAGTATCAGTGACATACATCTTTCTG	A/G	17	24009487	0.906
17027239725	ACGTGTGCTTCACTTTCTCCGAGAGATGGGGTGATGCAGAGTGTACACATTGGGGTGTTGG[T/C]GTCTCAGAACTAATTAGTGGAGGTTTCACAAGGCCTACACACAGCCCTTATAATATTAGTA	T/C	17	27239725	0.985
17028801656	TCCTCATCCCCAGGAGGCTGAGTTTTCAAGAAGCTTAGTTTTGTTAGAAGTCATAAAGAGA[A/G]TTGAATTTGATTTCATCTAATTTATTTTGGGCACCTAGGAAACAAATTGGAGTTGCCCAGA	A/G	17	28801656	0.601
17031881962	ATGTAGGTGCTAGGGTTTGAAATCCAGTCCTCCGTGAACACAGTCCATGCTAAATCTCTGA[A/G]CCATCTATCCAGCCCATAAAAATAAGATTTAGAACCAACACATGGATTACAAATGAAAAGA	A/G	17	31881962	0.911
17035082716	AAGGCCAAGACTATCTGGTCTTTATTGAAAACTTCAAAAACAAGACTCTTGACCTTGCACA[A/G]ATAAGACCCAGCCTTGACAATAGAGTCCCATCTGTGATACTTTGGCAAGTCCCAGTAACCA	A/G	17	35082716	0.898
17036686836	ACACACACACACACACACGGTCTGGAGAGGATAAGTTCCTTGAATTCATCTACTTCATTTA[T/C]CTTTTAATCCTGTTAAAGGTCCAGTCACTTAAAGGAATTGAGTTACTGTTGTTGTTGTTGT	T/C	17	36686836	0.867
17038219495	AGAGGACAAGACATGGTAGCCTAGAAATCCAGACATTGCAGAACAAATTAGGGCCAAGTTA[A/C]GCCAACTACCTATTCCTATCCATAGCCATTCACAGTATGGAACCTGGGCGATCAGTCTCAC	A/C	17	38219495	0.965
17039826041	AGGTTTGGGAGCTTGAAGGAATAGCTCGGTGGGTTCGCACATAGGTACCATATCTGAAGCC[A/G]CAGTTCCTAGCCACAGATCCCCTCCCTTCCTTCATTGTCCCTCAGAAGCTTGTGAAGATTC	A/G	17	39826041	0.998
17050250384	CCTTCATCTCTATCATGTAACAATAAAAGTGTCTGAAGTTTTGTGTTTGTGGCCAGGTAA[T/C]TAAAGTGATGGACTCTGGGTTATTGGGCTTTTTCAGCAGCAGATTCGATTCCTACACAT	T/C	17	50250384	0.962
17053549720	TGTAGTCATTAACATTAACAGAGAGGACATTCCAGTAAAATGTGTAATATGGACATAATG[A/T]GAAATATGCATTTTATTATACAAACGAGCCTTAGGCCCAGGCAGCCATCAACACTCTTG	A/T	17	53549720	0.548
17057521811	CAAAGGGATAATTCCATCAGAGTAGGAAAGCAGGACATCAAGCAGCAGGTATAGTGTCCA[A/G]TGCAGAAGGCCAAGATGTCACATCCTCATTCACAAGCACAAAGCAGAGAAAACCGAAAG	A/G	17	57521811	0.787
17059039727	ACCAATCAGAATGGCTAAGATCAAAAATTCAAGGGATAGAACTTGCTGGTGAGGATGGGG[A/G]AAAAGGGAAACACTCCTCCATGGCTAGACTGTATAATGACTGTGGAAATTAATCTGGCA	A/G	17	59039727	0.631
17060674272	GAACCTGGAGTTAAGTCTTAATATCTAGGACTACTTAAAGTTACCTATTACTGTTATTCAG[T/C]TTATTTTCCTAAGAATCCTGTGAAGTGTAGCTATTATTTCTGTCATATACAGGAGGAACTG	T/C	17	60674272	0.626
17068613889	TCCTTCATTTTCATGCAAATTTATTATGTTTACTCTAAAAAATTCATATGGGGTGGTGGA[T/C]GGAACTGGGCAATTCGTAGGGAGCCCAGGAGGCTCTAGAATCCCCCAGTTACTTCCTTC	T/C	17	68613889	0.727
17070447867	GAGGCTACATAAACACACATAGGTGCACATGAATAAATAGACAAGGGCTGGAGAGATTGGT[A/C]AGTGGTCAAGATCACTGTCTTGTCTTCCAGAGGTCATGAGTTCAAATCCCAGCAATCACAT	A/C	17	70447867	0.821
17072003151	TGTAATCAACAAAGAGAAAGGAGGGCTCAACAAAGTCTAAGTCTTTACAGACTAATGCCA[T/C]CCCTACCCCAAGTGCTGGGGCCTCATAGCCCAGGCCTTGAACTCTTCTTGATCATTCTG	T/C	17	72003151	0.932
17076739165	TAATGGAAGGTTGCGATTTTGAATTCCCCATTATTTTCTAGTTAAAGTGGAAATATAAAG[T/G]GATGCCAATATACATATTGGAAATTCAGTTGAACATATTGTCACCTATGGATTCCCTTT	T/G	17	76739165	0.663
17078245200	CAGATCCAAGGGGGTGGTGAGGCCTGGCTTTGAACTTTGTCTTAGGCTCTTGGCCTTGCGT[A/G]CTGAGCACATCCGTTGCCTCCCACAGTATAAGATACCCCGTCATAGTGTGCGGCAGGGATA	A/G	17	78245200	0.703
17083276274	TGGATGTGAGGAAGGTAAGGCTTCTGTAGTTTAGGAGTGAGGGGATTTGGGCAAATAGGT[A/G]GGGTTACAGGAGCAGAACGCCATAGCAAGTTGGTCCTAACAACTTCGGGAAACTAAGAC	A/G	17	83276274	0.789
17084867100	AGTTAAGATTGCTTGGTCCTATGATTTCAGGCAAATAATTTCACTCAAACAGTGTGAGAG[A/G]AAGCAACTGATTCCTTCTCTACAATGTCAACCTTTGACTTGGGAATCTCAACTCCGAAA	A/G	17	84867100	0.874
17086412600	CCATGTGTAAAGTTTTCCGTGTAAAAATGATCTTAAATGCCAGAGTCAACCTTCCCTAAC[T/C]TCATGGTGAGCGTCGGCAGACAGAGAGGATAGAGCAAGGGCGGGTCACTCTCTGATGAC	T/C	17	86412600	0.832
17088072070	TGTGTCATCCACCCAGCCAAGAGTATTTATGAACAGCTGTTAACTGACGAGGAAAAGGAC[A/G]CTTGCTTCGGATCACATTGAGGAATAGCTCATGTGAATCGCATTTATTGATCAGGACTT	A/G	17	88072070	0.954

17089629824	GATATCTTTAGCGTGACATGAAAAAGTGAAAATGATGTTCAGAAAGGATACATTGAACTT[T/C]GCTAAAAGTAAAAGACATTTAATTGTTGAAAGATACTACCAACAGGTTGAGAAGGCAAT	T/C	17	89629824	0.731
18003818313	GTGCTCTATGGAGCTGTGGGAACACAAGCAGCTGCTTGCATAAAGGCTTTGATTCAGTCA[T/C]AAATTAAGCAAGAAGGAGGCTGATTTAGTTCTACGGTGTTGAGGTTGAAGTTCAGTGCA	T/C	18	3818313	0.896
18006935933	GCTCAAGGTTTTGGAGGAATAAGCCCATCATGGTTGGGAAGGCATGATGGTGAGAGTGAC[T/C]TGGGGCTGCTTACACTGTATTGGCAGGAAGCAGCAGGACTCCAAGAACCAGGGCAGGGA	T/C	18	6935933	0.794
18010837308	TGAAGGGAAGTAAAATTTTAATTTTCTCAATAGAATTTTATTTGGTGTTTATTGTTGTTC[A/G]GTGAGTAATGTAGCTACATTTAACCAATTTGGTTTCAACTTTAACTAATATTAATTAAT	A/G	18	10837308	0.714
18012366935	AATCTTCCTAAGAAGGGTAGTTACGTTGAATATTGACATAGCATAGTCTTTTACTGTACAT[T/C]CAATTGTTTACACACTGTGCTCTTGATGGATGGAATTCTATCCCAATCAACCCAGGATTAA	T/C	18	12366935	0.837
18013887135	GGACAATTTGAATTTTTTTTCCTCTTTGGTTAATTGGTCTATTAGGAGATAATTCTATGT[C/G]GACCCATATATAACAAAGTGGCTAAAATAAATTCAATGTATAAAGCATAATGTTGCTTA	C/G	18	13887135	0.727
18015474943	CTGTGTACAGGATATCTTGGTTGGTACCTTGAAAGACATGAGGGTGATTCAGATTTAAAG[T/G]TTTACCTTAAGAAACTTGCAGGCAACCATAAGAAACCTGTTATAGATGACATATTGGAA	T/G	18	15474943	0.924
18017045165	AAACTGGAAGGCTTTTTTTTTTAATTACTTATTTTGTTGTTTTTAATTCTCTAGAACACA[T/C]TGTATATATTTGGATGGTAAAGTATAAAAATGCACAACTATTTCTTTTTCAAAGATGTC	T/C	18	17045165	0.543
18020212418	AGCACTTCCCTCCTTACTCCCTGATTTTGGTATTCGGGGTACAGTAGATGCTCAATCTAC[T/C]TACGTGTCCTAAGAGGAAGAAGAAAGAGGTCTTGCTTTACTTTCTTGATAGCCTAGGAA	T/C	18	20212418	0.873
18021940656	CTGGAGGAAAAGAGGTGGTCCCTCGATTTTCCAGCCAGACAGTCTTTACTGAGATTTATTT[A/G]ATCGGGAACATAGATTGAAATATATTCACAGAGAAATACTCAGAAATTAGGGAAATCACAA	A/G	18	21940656	0.752
18023488446	TGAAAGAGAGTGAGGCATGGAGAGGTCCTGGAGAGAGAGCTTAACAGTTCCTTAAGTGAAG[A/G]ACAGGAGGAGAGCTTAATGTGGTTGATCGAAAGTATGGGAAACGGTGACACAACTGGAGTT	A/G	18	23488446	0.96
18024995574	AAATGGCAAACTAGGTCTGGACAGAGATGAACAATGAACCACAATGAGATTTTACAAGGTC[A/G]TGGGATAGAAATTAGTCATCCAGATGGGTCTGCTGAGATCCAAGGCCATGGCATCTTCATG	A/G	18	24995574	0.908
18028487492	TGTTTTAGGAGACCAGGCTGTTTTGTGAATAAAACTGAATGCATGTTTGTGTCATGATAA[A/C]ACTACTGGCTTCTTGCTCTTGGGTTTTGGAGGGCTGGCTTGGGACTAGGGCTGGGCTGG	A/C	18	28487492	0.813
18032253306	AAAGTCGGCTATGGGAAGAGTCCACTTCTCTAACCCTTCAGTTCCCAGCCCTGCAGTAAGC[A/G]GAGGCTCTGCAGCCTTCAGCGTGGGAGTGCGGAGCTCAAGACCAGGCTTCTCTATTGTGCC	A/G	18	32253306	0.673
18033919873	GGCATTGCAATCCTCTGCAGTTCCTTCAGTCGTTCCTCTAGTTTTCCATATTGGCCCCCAA[T/C]CTCAGTGCAGTGGTTGGCTATAAGTTTCTGCATCTGTCTCAGTCAGTTCCTGGTAGAACCT	T/C	18	33919873	0.612
18042542433	GCATCTCTTCATGCATCTTCAACCGACTTTGTCATAAGCAACATCAGACTATTTCCGTTCA[A/G]AGTGTGATTTCTGTCTTTACAGACAGGGTTAAAAAGATGCTTCTGTGATGGAGTACTACCT	A/G	18	42542433	0.862
18044256339	CCCTTTTCTTAATAGCTTCCTGTACTGTTCAATAAAATCTGGAGCATACACAAACAGACA[T/C]GGACATGGGAACAGACAAATTCACAAGATAGCATTCAGGCATGCATGGGTACAGATTCA	T/C	18	44256339	0.943
18047721977	ATTGACTTGATATAAAATGTGTCATTTATTAAGTCCTTCCTGTGTGACTTTTAAATTCCA[T/C]GTTGAAAATGCTATTTAGTGTTAAAACTTTATTAGACACCAAAACACAGCTATTTTCCA	T/C	18	47721977	0.901
18049435104	TCTCTTCATTTTGCCAATTTTCTTTTCTGTGCAGAAGTTTTCCAATTCCATGTAGTCTCCG[T/C]TGTCAGTTTTTGGAGTTTTTTCCAAAATATTCTCAGTGATGTAGGTGTCTTGAAATGTTTT	T/C	18	49435104	0.952
18051039405	CAGCTCTTTCTTGAAAGCCTTTGTCTTACTTCCCAGAAGCACACACGTACGATCTAGTAG[T/C]CTTTGCTATTAGAGTAGAAGTGCCAAGATAAATGCTGTAGGAATGAATATAGATTGCCA	T/C	18	51039405	0.885
18052589887	ATGGGATGCAAGACAGTACAAAGTAGATGAACAGAAAAAAAGATGATTGGGAATGAGGTAA[A/G]TGGGGTTTTGGCTCTCAGGTAGTAGGGCTCAATTATTTAACTCCTTCATATAGCCAAGAAA	A/G	18	52589887	0.959
18055800448	CGCACGGTGTGGTCCGCAGCATTCCCATTCAGCTCACAGCTGAAAACTGCGTGACGTATGA[T/C]TTACACAGAGCCCGAGTGAGCACGTTTGTAGTCCTAGGTAATGTTGTTAATGCCTGGAAGC	T/C	18	55800448	0.853
18060832258	TATATAAACATAATACGCCCTCTTTGAAAAAGCCCCGAACTGCTCTGGGTGAGGTACCTAC[A/G]CCTCTGTCCATTACTAATCAACGTGTACATGGCTATGGCCGAAGTGGAAATGTTCGTTATA	A/G	18	60832258	0.961
18063882170	GTTGGGTCGTTCTCAGAAAATGTTTTGATGTTGCTGTAGTCATTTCTTTGGTCCACGTTGG[T/C]GTGATTTGGGGGGTACATTTTTTTAAAAGTCATCAGTAAAGATCTGCCTCAGCTGAAGTCT	T/C	18	63882170	0.829
18065423483	GTATCTACAACCTTATGCCTCAAACCTTTTGTGGTTCTTTCATGTAACAAGTGGGACTTC[A/G]TAATACGATTAGGGAGCTTGGAAAAAATTATGTTCTGGGCAAGTAGAGTGCATCACAAA	A/G	18	65423483	0.896
18067006147	CCCATTCCACTGTTGAAAAGAAATATGTGCATGAATAGCTTTGCCTTTCTAAAAGTCTTA[T/C]GAAGCTATCTCCACTTTTTTAAAATCTGTTTATTCGTCTTTAAGTGTTTCACATCAGGG	T/C	18	67006147	0.774
18073132804	AGCCTCTGAACCATTTCTCAAACCTCTACCCCCTCTTCTTGTCTGGTACCCCAGCCCATG[A/G]ACAGTGCCCACACTTAGGAGTCTGCTGCTTCAGGTGCTTCTCTCTGGAAACCTTCCAAC	A/G	18	73132804	0.859
18074697767	GGAAGTAGGTCAGGCCAGCATGGTCCGGTTAGAAGAGCTCAGTATCGACCCCGCTAGAGTG[A/C]AGAAAGCTCATCAAGAAAAAATTCCAGATCTCCATGTCAATGTCTCAGAGCAAGGACAGGC	A/C	18	74697767	0.994
18076353386	AGTTTGATCTCAGTAGCATGGTTTAGTTATTAAACGTCTGTGCTCCCACTACTTGACTATC[T/C]CTTCAGACGCCATTCTTCCTATGGTCCTTTAGTCCATAATTCTACTTTACTCTTCTACATA	T/C	18	76353386	0.971
18077866227	ATGAAATCTGACCTACTTTTTGTCTGTTTTCGACATCATTCTAGATACAAAGAAATAATC[A/G]GCTTGCTAAGTAATTCCTGCTGTTTCCCCCTCTGTACCTTCCCCTCTTGCTCTGTCTGG	A/G	18	77866227	0.775
18080901061	TGCGGATTATATTAACCAGCCAACTGAGTTTTATAAACTCTGGGATCCAGTTCTGCATCAC[A/G]TTTTATAAAATGCTTTCTTTTACTATGCATATTTAGAAATACAATGAATGTGACAAACACA	A/G	18	80901061	0.876
18082458087	TTATACTTTCACAGTGCATTCTGGCTGGGAAGGTTGTGTTGGTTTCTCAGGAAGGATGTGA[A/G]TGAATGTTATTTTCAGTTATCGATCAATTTTGCAAGGGCTAATTGTCCATAGAAGTTGCTT	A/G	18	82458087	0.951
18083991780	GCAAGAGGCCTTCAGCATAGGGAGGTGCAGTGCTGCTTTTGTTTGCTAATTCCAGTTCCCA[A/G]TCTGACTCCTCTGTCTGCAGGAAAGGAGGTAGGCCCCTTTCTTTTCTGTTGATTCCATTTC	A/G	18	83991780	0.904
19001746598	AATCAAAATGTTTCTTTATGAGAAGGCAGCTTATCAAGAACCACACTATTTCATCAATCC[A/G]TACTCATCAGGAATAATTCAGTGGGTCCCCAATCCACTGCAAAAGCTACCTGGTACATA	A/G	19	1746598	0.889
19003281882	AGGTTGATGAGTGTAACTTATTTTACAATCATTCATGATTTGAAGTGGAAAGGCCTGACCA[A/G]TGGTAACTAAGGAAAAGTGGAGGGATTCAATGAAAATATGAAATCATAAAATTTTAGATAA	A/G	19	3281882	0.981
19004905218	TGAGAAAGAATGGAAATTAGGTATTTCTCAATAAGTTAGATTCAGCTCCTTCACATGAGTG[A/G]CCTCGGTAACCTATACTTGTATCCAAACCACTTGTTCTTGCAGAAACTACTCCTCATCTAT	A/G	19	4905218	0.937
19010030870	GTGTTGCAGGTGAAGTGAAAGAACCTTATTTGCACATGGGATTTCCTTAGCTAATCTAAG[A/G]GTATCTGATAAAAACTAAAAAGATACATTTACTTAAAATGAAGTAGAAAACTTTATACC	A/G	19	10030870	0.645
19018014929	CTCACTGCTCTGACAAAATATTTGGGAAGAAGTGACTTAAGGTAGAAAGGGTTTGTTTGGG[T/C]GCAGAGTTCGAGAGTACAGTCCACCACGGTATTGAGAAGGCATTAACATTGTGAGGTAATT	T/C	19	18014929	0.556
19026469417	AGAATTGTACACTGGCCTTCATGCAGAAACTGTGGCATGTATGAACACAGGAAAATAAATT[A/T]AAAAAAATCAGAATTCAAAATATCAAGCAAATTTCCCACAGGATTATGGATACTATGAGCT	A/T	19	26469417	0.841
19032800853	CGCCAGAGACCAAGGCCCAAGGCAGGGAGCAAAAGCAGAGAAGGCTGAAGGGCTAAAGCC[A/G]TAATTAGAAAACATCTTTTCACGTGGAAAGCTAACAAGGAGGAGGGAAACCCTGGGGCA	A/G	19	32800853	0.986
19036121822	TCAAGGTCAGCCACACATTTGTCAGGCTTTCCCTTAGTATCGCTTGCCTTGTGCTGACAAA[A/G]GCATCCCTGCTGGCCCGCAGCAGAGAAGACCCCGCCTGGGGTGAGGACACCACAGTAGCAA	A/G	19	36121822	0.631
19037837962	GGGCCATAACATAGGGACATACCTGGCCTTTGTTCCGTTCCTCATCTTCCCAGGTTAACAT[T/G]TCAGAACTTTGTAAGGGGTTTTGAGATTTTCTAATACCTTGAAAATGGGTAATGGAGGCTC	T/G	19	37837962	0.815
19040876227	GGCCTTGAACTCAGAGCTTACTCTGCCTCTGCCTCTCAAGCACAGGGGTCACTGCCCTGCT[A/C]TTTTTTTTGGGGGGGGAGACATCCACTTGGCCTGACTCCTCAGAATCTCGAGTGTTGGGGT	A/C	19	40876227	0.565
19042542340	AGGAGAGGAGACACACAATGTTTGAGAGGCTGTGAAGAAGTGGGTTTTATACTCACTACGG[A/G]GCAGCAATTTAAATTGGCCCAGCCTTGCTGAAGATGATCTTTATATTAGTTTACAAATCTG	A/G	19	42542340	0.688
19045796278	TACTTTATTTTGTTTTGGTTTTGATTATACAAAGTCATCAGAATGGGCTTAGGCTGGCCTC[A/G]GTGCTTCCTCTCACAAATCCTGTGATGACAGACATGTACTAGCATGTCCAGGTCATACAGT	A/G	19	45796278	0.872
19047315592	GAGCTCTGCCATTGGATACCCTCTCCCAGGAGTTAGCTCTCAGAGTAACAGCCATTTCTCA[T/C]GTCATAGTTGGAGCTGGCCTACTTAAATGGAAGACTTGGGTAGTCAATAGTAGTCAAAAGA	T/C	19	47315592	0.918
19049284552	TTCTGTGTGCTGTGTAAACAGCGCACGTTGTTGTCCTGGTGAGCTTGTCTTTTAGCAGGCC[T/C]GCATCACGGGCAGTGTCCTAGTTAGCTTTGTCATTGTGTCAACTTGACACAGCCTAAAGCC	T/C	19	49284552	0.713
19054238555	AGTTGATTATAGAGTCCGTACCACAGTACACCCTTGTGAGGAAGAGTTGTGCGTTCAGATC[A/G]ATCTAACATGGTGACTGTGGAAAGAGAAACCACCCCTGCCACTAATCCTCCACCTGCAGAA	A/G	19	54238555	0.932

20000047516	TCAACGGATTGTTCTACTATATTGTATGTGAACTCCGTCTTCTTATTAGAACATCTTTCCT[C/G]TCCTTTCCTCTGTACTTCTAACTTGTGTGTTTTTAGCCTCTGAAGGTCAGCCTCAGGGTGC	C/G	20	47516	0.749
20001565758	TCAGCGAGTCCCCAGGGAGAGGGTCAGGAGCAGTCACTAAACTCTACTGGGTACTAATTT[T/C]CTGACTTTAAAAGGAATGGGGAGATGAACTCTCAAGCTGTCTCTTTTTAAAAGCGTGTA	T/C	20	1565758	0.862
20003130757	AGTTCAGACTCCTCCAGCCCTGAAGCCCATTTCTACCCTTTCGGATGCTAGGTCCTGAAA[T/C]GCAGGAGCAAGAGAGGTTGACGCCTACAGATTCACAGGTCATGCTCCGGGAAGAAAAGC	T/C	20	3130757	0.873
20004715045	ACCACAGAGGTGGGGCTAAGAGCAATGGCTGCTCTTGCAGGGACCATGAGTTAGGTTTCC[A/G]GCACCCACCTGTGGTTCATAATTGAAATGGAAACTTCTAGTTTCTTTGGAAAGTCGGTT	A/G	20	4715045	0.937
20007904736	AGTGGACGGTTAATGATCCTTCTGAGCAGATTGTTTATGTGAATGAAGCCCTTCAGAGCCC[A/G]CGCCTCCCTCTCTGCTGGTTTTTGCCCGTGTGCTCAGCTCAGTTGAGGAGTGTGTGAACCC	A/G	20	7904736	0.731
20009465495	CACATACACACACAGAGAGGGGGGGGGAAGACCCCTCCTTGCTACATCACACTACAGACA[A/T]GTATGCACACAAACTTTTGAAAGATATTATATACATTCCTATCAAGTGTGATGGGATAG	A/T	20	9465495	0.907
20012527514	AAACATTTACCATAAATTAGTTCAGAAAAAGAGATGGGGGGGGGGATTTCTTTTACTCCTA[A/T]ATATACTCCAAGTTTTTGTAATTGCCAGGTATGATCTGTTGTCTATTCCCACAAAGCCCAA	A/T	20	12527514	0.973
20014310650	AGAAGCTGAAACCCTGGAGCCCTATCAACTCTTTCAAGGGTAAGTGGAGGGAAAGGGAGCC[A/G]CCTTCTTCAGGCAGAATGCAATCCATCATTATGAACTTACCGGCAGCTTGTTCACCAGGGA	A/G	20	14310650	0.726
20016047305	CCCTGAAGCTTGTTGAGCCGAGCTGCTTTATGGAAGGATAGAAGGACGATCCAAAAGGCC[T/C]GGTTGCTAGTGGGAGGTTTTCTAGCCAGAAGGTCGCCCCTCCTGGCTCCATAGAGCCCC	T/C	20	16047305	0.951
20017608327	CGTTATGAATTATTACCACGCAGCACCAAGCCTTCTGCGTACTCTGCATTTGCTGCACTG[T/G]TGGCTCTGCATAAAATACAGAGATATTGGTATGAGCCTGTCTATCGGTCCTCTGGACAC	T/G	20	17608327	0.988
20019324306	TAGGTCTTTCCAATCCCACTTAATTTTGGTTAGAGAGTGTGGCTTGAAAGAGCAGCGGCT[A/G]TGCTCTAAGGTTACAGGGCCAAGTGCTACCTCATGCCGCCTTCCCCTTTACCCAGCATG	A/G	20	19324306	0.82
20020963417	GAAACTAAATGTTCATTTAAAATATTGAAATAGATTTTAAGCGTATTAGAGGGCAGGCTGG[A/G]AGAGGTCAGCTCTCACAACCATGCCCACTTCTAAAAAGGGTTTTGAACTGTCTAGGTTATT	A/G	20	20963417	0.849
20022594060	TTAGCCATTTCCCAGGCAGTGCTGAAAATTTGCCCTTATCTTCCCAGAATTCCATTAGATT[T/C]TGTGCCTCCCAAAATGAAATGGTGTCTGTGGCTGAGAAGGTAAAGCGTTAGATAAAAGCTT	T/C	20	22594060	0.974
20025197576	GCCTCAACCACAAGGCAATCCTCCTGTGTAGCATCTGAAAGCCAGAGTTAAACAAGTGCCC[A/G]TGTCATCCATCTTGGGAGAAACCCTTTAAGTGCCCATCAATACAGGACTGGCTAATTAACC	A/G	20	25197576	0.892
20026707550	TCCCAAAGCTCACCTCAAGTCACATGCTTTCTTCAGCAAGGCTGCGTTTCACAACCTTCCT[A/G]CACAGCTCTACCAACCCCACCAGGGACCGAGTGCTCGAATATCTGAGCCAGTGGGTATCAC	A/G	20	26707550	0.812
20028878254	GGATGACCCTGACCATCTGATCTTCCTGCCTCTGCCTACTGAGGACCCTCAGTTTACTCG[T/C]GCTGAGGAATGCAATCATGGCCCCTTGCACCTTAGCAGGTAGTCTTCCAATCAGCTGCC	T/C	20	28878254	0.954
20031071864	ATGACTCACAACCATCTGCAACTCAGGTTGTGTTTCAGAGGATCTCACCCCCTCCTCTGAC[T/C]TCCGAAGGCACCGAATATACAAGGTGTATACACAGCATACACGCAAAGCACTCACCCACGT	T/C	20	31071864	0.806
20032653341	AGTTCCTGTTGAGTTTCGAAGCCAGTCAGCAGTAACTGCCCATTCTATTCTACAAAGTCTC[T/C]TCAACCTCCCTGCCACTGGCCATCCCCCCATCGGCATACCTTGGGCACAAAGGCTACGGAA	T/C	20	32653341	0.963
20034235827	CCTTAAAGCCATGAACTGCCTCTTTTCACTGGAATCTGCCCTGGGGAGCCATGGACAAGG[T/C]CTTTGCCTATAGAGATGTTGTCTAATCTCCCATAGAAGGACTCTCCACGTTCCCACCAA	T/C	20	34235827	0.558
20035793807	CCTCTTGTTAACTTACAGTAAGGAATAAACTCCAACATGAGTTTGGGAAGAATAGTTATA[A/G]CTCTCAGAGTTGTTCATTTTTCATATAACTACTGGACACCGATCTGCCGCTGGAGTTCC	A/G	20	35793807	0.808
20037370145	GTGGATATGCCACCACTCCAGGTATTATACCTAAGTGCTGGAGATCTAAACTCAGGTTTA[T/C]ACACTGTTCCCTGGTCAGCTAGACACAGACTGAGCAGCAGCTGACTCCCAGACTCAGCA	T/C	20	37370145	0.663
20040509529	TAATAAAGTCTTCATCGCCCACAGCCATCATGGCCAAAGGGTTACATGTGCTTCTCTTCTG[A/G]TCACACTATGCATTGTGAACATTTGTGTTCTTCATTGTTTGCTTTATTTTCACTGAAAACT	A/G	20	40509529	0.918
20042016047	GAGGTACTAAATATACCACATTTGAATTTTTATCGCCTTGTTTTAGTCTTGCCATAAATC[T/C]ATTCAGTCTTTGAAAAGTTTCTTGGTTGAATTACTTCTCTAGTAACCTCTTTTCCTCTT	T/C	20	42016047	0.916
20043563914	TGGTTGGGTTTTAGCACCCAGATACTGGAAGGAGGGAAGTGGGAGTATTGACACCCTTGAG[A/G]GTCTCTCTTAACTGGCAGTGACCTTGGAGGGCAGAACAGTTCTTACTGGGAACCCTCCTCA	A/G	20	43563914	0.919
20045068619	TGCTGTGGTGAAATCAAATCATCCGATACATGAAACATAGTTACAATAGCGCTTGGTACA[T/C]AGTAGACACCTGGTCAACAAGAGCTACTGTAGCATTGTCCTTAACCGGCGCATGCGCGA	T/C	20	45068619	0.915
20046634456	TCACTGGGGAGTTGCCACCACAAAGCAACCTCACACACCCAAAACCGGAAAAGCGTTTCC[A/G]GGAACGCTCCAAGAGCAACACACCGAGCGCCTTTCTGGAGCAGTAAGAACAGAGTCGCC	A/G	20	46634456	0.761
20048250172	TCTCTCTACAACTTTCTGTAAAACTGTACATTCCGAGACGGACACGGTTAATTAGAAAAC[T/C]GAGACTAGGACAACAATCTCCCATGCATGGGGTGGAAAGAAATGAAGATGGCACAACTC	T/C	20	48250172	0.904
20049799754	TTCTTACCATAGAATCATAATGTGGGCATTAATATAATTCTTATAAATGGAGAAATTCAGA[T/C]TTAGGTGTATGAAAGATGGGTATTAAATAATTTGTTCATGAACATGGAGAAAATTGATATG	T/C	20	49799754	0.686
20051345604	CTTTGGACAGTAGGATAGAAGGGAGGTCAAAGAGTTTGAGAACCTTTATTAAAACTAGGAT[T/G]TTAAAAATCTAAGCATACAGAAGCATTTTTTTGACATACTGGTACCAGTTCCTTTATATAT	T/G	20	51345604	0.525
20052872439	AGAATTATAGCAAACGTTATAATGAAGTCTCTAAGCAGTTTCTAAGAAACAGCAGATATCC[A/G]CACAGACTAGAATACCCAGAAAATTTTTCAATAACTTTAGATGGAGAAAAGAAAATATTCA	A/G	20	52872439	0.689
20054465630	TCTCTGGCATGGATTCTCTTTGCTGGTCTTTGTTGGGCTTCACTGATGATGCTTTGGAATT[C/G]ATTCAACTTTCTTTCTTCTCTGGTATTCATTTGTCCTAACTTTGTAGAGAAAAATGCACCA	C/G	20	54465630	0.967

Supplementary table 6. SNPs (n=894) selected for linkage analysis.

Marker Name	Sequence	Nucleotide_Variant	Chromosome
Rn34_1146593376	CTATGCAGTAAGAAAAAAAGAAAGAAGAAAATCCTATTTTTGTCGAAGGGGACTTTGAGAT[A/G]GAAGGTGTTCTTTTCAACTGTATCAAAGCCAGCACAATGTTTTAAGATGCTAAGACAAAAA	A/G	1
Rn34_1148739042	CTGAGACCCAAGAAGCTAGTTGAGTTGAAAATTCTCCATTGCCTTGAAGAACAGTACTAAA[A/G]TATCTTCAGCTTTTCTAATGCTTTAGCCTGTGTTTTACTATATCTGCTTCTGAAAAACTGT	A/G	1
Rn34_1173205180	CCCATTGCTCATTAGAGGACATATTTGTATTAGATGAGGCTGGGTGGGGGCCTTGCTTGT[A/C]CTCCCACTACCTAAACCAGTAAGGAAGCTCAGGAGTCAAAGGAAGTAGCTGAGGCTGCC	A/C	1
Rn34_1211246657	TGAAAGGATTTACAAGCAAGAGGCTGGTGAGACTCTGCAATACCAGGCGTCTACCGTAAGA[A/G]GGAGCTGCGGGAGGCTTTTCGATTCAACAGGGACAATTAAACTTTGTTGGTTTGGAACTGC	A/G	1

Supplementary table 7. SNPs within the QTL region, selected for Taqman genotyping.
